# Structural and spatial chromatin features at developmental gene loci in human pluripotent stem cells

**DOI:** 10.1038/s41467-017-01679-x

**Published:** 2017-11-20

**Authors:** Hiroki Ikeda, Masamitsu Sone, Shinya Yamanaka, Takuya Yamamoto

**Affiliations:** 1 Department of Life Science Frontiers, Center for iPS Cell Research and Application (CiRA), Kyoto University, Sakyo-ku, Kyoto 606-8507 Japan; 2 Institute for Integrated Cell-Material Sciences (WPI-iCeMS), Kyoto University, Sakyo-ku, Kyoto 606-8507 Japan; 3 Gladstone Institute of Cardiovascular Disease, San Francisco, CA 94158 USA; 4 AMED-CREST, AMED 1-7-1 Otemach, Chiyodaku, Tokyo, 100-0004 Japan

## Abstract

Higher-order chromatin organization controls transcriptional programs that govern cell properties and functions. In order for pluripotent stem cells (PSCs) to appropriately respond to differentiation signals, developmental gene loci should be structurally and spatially regulated to be readily available for immediate transcription, even though these genes are hardly expressed in PSCs. Here, we show that both chromatin interaction profiles and nuclear positions at developmental gene loci differ between human somatic cells and hPSCs, and that changes in the chromatin interactions are closely related to the nuclear repositioning. Moreover, we also demonstrate that developmental gene loci, which have bivalent histone modifications, tend to colocalize in PSCs. Furthermore, this colocalization requires PRC1, PRC2, and TrxG complexes, which are essential regulatory factors for the maintenance of transcriptionally poised developmental genes. Our results indicate that higher-order chromatin regulation may be an integral part of the differentiation capacity that defines pluripotency.

## Abstract (web summary)

Higher-order chromatin organization regulates the expression of transcriptional programs that control cell function. Here, the authors show that chromatin interaction profiles and nuclear positions at developmental gene loci differ between human somatic and pluripotent stem cells.

## Introduction

One prominent aspect of stem cells is their ability to differentiate into other cell types. Specifically, pluripotent stem cells (PSCs), including embryonic stem cells (ESCs) and induced pluripotent stem cells (iPSCs), can give rise to almost all cell types within an animal’s body. In the pluripotent state, developmental genes are rarely expressed in PSCs, but should be properly transcribed in response to extracellular differentiation cues. Therefore, in order to understand the differentiation ability of PSCs, it is important to know how developmental genes are regulated in order to promptly undergo transcription upon stimulation.

Epigenetic regulation by histone modification plays critical roles in transcriptional programs that govern various biological processes. In PSCs, distinct histone modification regions, termed as bivalent domains, have been observed in the promoters of many developmental genes^1–5^. Bivalent domains have both transcriptionally active (H3K4me3) and repressive (H3K27me3) histone marks, which are independently catalyzed by the trithorax group (TrxG) and polycomb repressive complex 2 (PRC2) complexes, respectively^6–8^. Moreover, polycomb repressive complex 1 (PRC1), which has ubiquitin ligase activity, binds to bivalent domains by recognizing H3K27me3 and maintains the inactivation state of developmental genes^9^. Notably, bivalent domains are frequently occupied by paused RNA polymerase II^10, 11^, suggesting that bivalency is a mark of developmental genes that are in transcriptionally silent but poised states in PSCs. Most of the bivalent gene loci in PSCs lose either active (H3K4me3) or repressive (H3K27me3) marks upon PSC differentiation^1^. Conversely, during somatic cell reprogramming, bivalency at developmental gene loci is reestablished in their promoters^12^. Furthermore, knockout experiments have implied that epigenetic modifiers that establish bivalency might be required for developmental plasticity^13–15^. Thus, the regulation of bivalent modification is closely related to the cellular differentiation of PSCs.

In addition to histone modifications, higher-order chromatin arrangements through three-dimensional (3D) architecture and subnuclear localization are also key factors for the control of transcription. Previous studies have shown that upon the induction of PSCs, pluripotency gene loci, including *Nanog*, are repositioned from the nuclear periphery to the nuclear interior^16^, and that the promoter–enhancer looping structure of the pluripotency genes is reorganized before transcriptional activation^17^, suggesting a crucial role of chromatin interaction during the induction. However, it remains unclear whether higher-order chromatin structures at developmental gene loci are altered during somatic cell reprogramming and how these structures are regulated in PSCs.

Generally, chromosome conformation capture (3C)-based methods are widely used to determine the physical interactions of distant DNA loci^18^. These methods quantify chimeric DNA fragments that are derived from proximity ligation events. Among them, circular chromosome conformation capture sequencing (4C or 4C-seq) can identify spatial interactions of a single genomic locus on a genome-wide scale^19–21^. Since 4C-seq focuses on one genomic locus as the analysis target, the required total read number for analyzing chromatin interactions with 4C-seq is much smaller than that with the Hi-C method, which detects all combinations of genomic interactions^22^. However, conventional 4C-seq has some disadvantages in the library preparation, which requires inverse PCR using a circularized 3C library as a template, prior to quantification by massively parallel sequencing. This inverse PCR results in the amplification of DNA fragments that have the same 5′ and 3′ sequences derived from the primers, and the artificial PCR duplicates prevent quantitative analysis of the 4C-seq data. Furthermore, the circular 3C library construction process contains DNA that has been digested with a restriction enzyme (typically, a 4-bp recognition enzyme); therefore, it has a potential bias associated with the uneven distribution of the enzyme recognition sequence. In addition, conventional 4C-seq is restricted to one genomic locus and cannot reveal the interaction profiles at multiple regions in parallel.

In this study, to investigate the global chromatin interaction profiles around dozens of developmental gene loci in detail, we develop a multiplexed Splinkerette^23^ chromosome conformation capture combined with high-throughput sequencing (ms4C-seq) by improving upon previously reported 4 C methods^17, 24, 25^. The ms4C-seq allows simultaneous analysis of chromatin interactions from multiple viewpoints while limiting the effects of PCR and sequence bias. Using ms4C-seq, we examine the genome-wide chromatin interaction profiles of multiple developmental gene loci before and after human somatic cellular reprogramming. Our results demonstrate that the global interaction profiles of developmental genes dynamically change during the reprogramming process. Furthermore, we find that the position of pluripotency gene loci and also developmental gene loci change from the nuclear periphery in somatic cells to the nuclear interior in human PSCs (hPSCs), and these changes are accompanied by changes in the nuclear positions of their interaction target loci. Finally, we show that bivalent promoters have a tendency to colocalize with one another in hPSCs, and that the knockdown of components of PRC1, PRC2, or TrxG complexes disrupts bivalent gene colocalization, thereby suggesting that the histone modification machinery plays an instructive role in higher-order chromatin organization. Thus, our findings illuminate the regulatory mechanisms of developmental genes that are crucial for the cellular differentiation of hPSCs.

## Results

### ms4C-seq reveals chromatin structures at bivalent gene loci

**Fig. 1:**
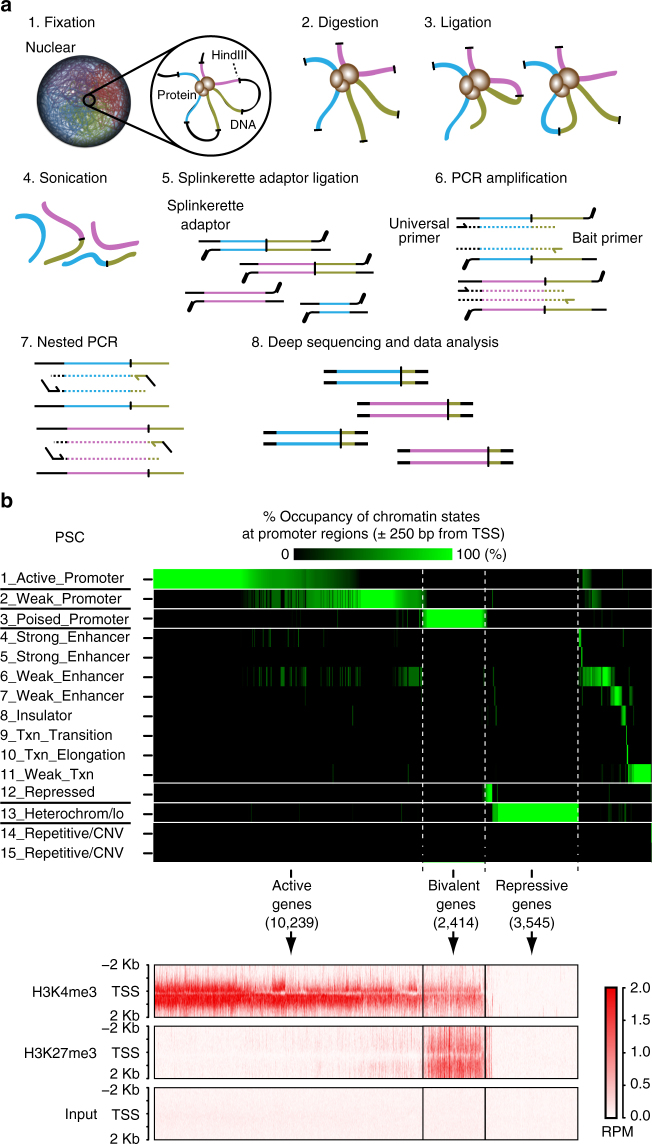
ms4C-seq and definition of genes by chromatin states. **a** Schematic diagram of ms4C-seq. Step 1: fixation of a nucleus with formaldehyde. Step 2: digestion of DNA with *Hin*dIII restriction enzyme. Step 3: preparation of chimeric fragments that are derived from physical proximity ligation of DNA. Step 4: fragmentation of those chimeric fragments. Step 5–7: ligations of Splinkerette adapter^22^ and amplifications of target loci by nested PCR with a universal primer for the adaptor and specific primers designed for bait loci. Step 8: deep sequencing by a next-generation sequencer. See also Supplementary Table 1 and Methods for details. **b** Definition of transcriptional states of 18,984 genes (bivalent, active, repressive, and other gene groups). The top heat map represents the occupancy of each chromatin state (%) within TSS ± 250 bp for all refseq genes in PSCs (ESCs). Bottom heatmaps indicate H3K4me3 and H3K27me3 modification profiles (RPM values) around TSS for active, repressive, and bivalent genes defined by chromatin states in PSCs (ESCs). Histone modification profiles are shown within TSS ± 2 Kb

It has been shown that pluripotency gene loci are dynamically reorganized before their reactivation during somatic cell reprogramming^17, 26^. Here, we focused our attention on developmental genes, which are frequently marked in PSCs by both active (H3K4me3) and repressive (H3K27me3) histone modifications, called bivalent domains^1–5^. To examine the chromatin interaction profiles of the developmental genes (especially bivalent genes) at high resolution, we tried to perform a 4C- (circularized chromosome conformation capture) based massively paralleled sequenced method (4C-seq). Many versions of 4C-seq have been reported to overcome the disadvantages of 4C-seq described above by introducing several modifications, such as sonication-based fragmentation, adaptor ligation, and multiplexed PCR^17, 24, 25^. Thus, we combined the beneficial features of those modified methods and developed a version of 4C-seq, termed Multiplexed Splinkerette^23^ chromosome conformation capture combined with high-throughput sequencing (ms4C-seq) (Fig. 1a; see Methods). To avoid nonspecific priming in the enrichment of bait loci by adaptor ligation-mediated PCR, we employed splinkerette-PCR^23^.

To select the baits for our analysis with ms4C-seq systematically, we used publicly available data sets for chromatin-state segmentation determined by ChromHmm^27^, which integrates multiple genome-wide data sets such as ChIP-seq data across multiple cell types using a multivariate Hidden Markov Model (HMM) to characterize chromatin states. In a previous report that applied ChromHMM to nine ChIP-seq plus input ENCODE data across nine cell types^28^, all human genomic regions (200 base pair interval) were assigned to 15 chromatin states including a “Poised Promoter” state. Comparison with ChIP-seq data sets^29^ showed that virtually all the genes whose promoter regions (±250 bp from transcription start sites (TSS)) are categorized into a “Poised_Promoter” state by ChromHmm have both H3K4me3 and H3K27me3 modifications in their promoters. Therefore, hereafter, we designate these genes as “bivalent genes” in PSCs and human fibroblasts (HFs) (Fig. 1b for PSCs and Supplementary Fig. 1 for HFs). Likewise, we defined genes that have promoters categorized into “Active/Weak Promoter” and “Repressed/Heterochrom” by ChromHmm as “active genes” and “repressive genes”, respectively, in both PSCs and HFs (Fig. 1b for PSCs and Supplementary Fig. 1 for HFs). Consistent with a previous report^1, 30, 31^, the number of bivalent genes in PSCs is much greater than that in somatic cells (HFs) because the gene number of the bivalency lost is greater than that of the bivalency reformation during PSC differentiation.

**Fig. 2:**
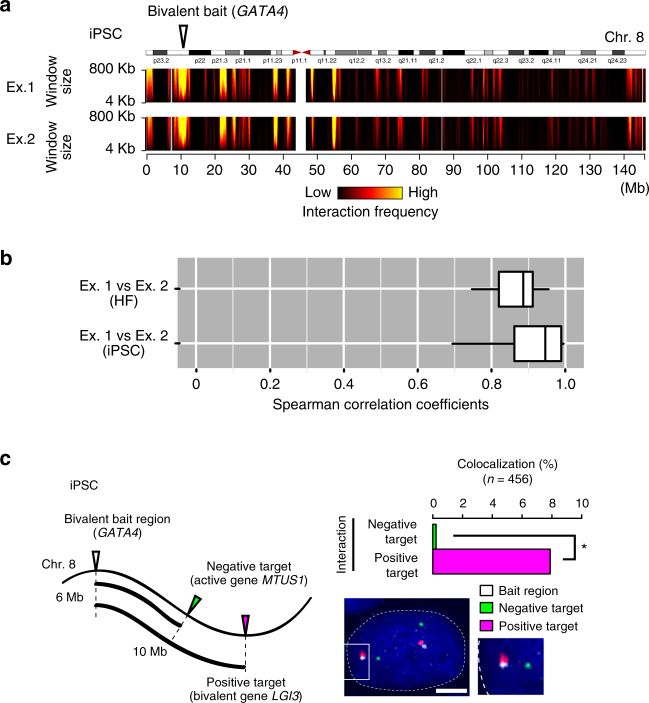
Examination of chromatin interaction profiles at bivalent gene loci. **a** *Cis*-chromosomal interaction profiles at the *GATA4* (bivalent in PSCs) gene locus in hiPSCs. Interaction frequencies of the *GATA4* gene locus, as determined by ms4C-seq, are presented by the domainogram in biological duplicates (Ex. 1 and Ex. 2). The color scale represents the log_10_ (*p*-value) interaction frequencies from low significance (*p* = 1) to high significance (*p* = 10^–10^), with colors from black to yellow, respectively. **b** Analysis of the reproducibility of two independent ms4C-seq experiments in HFs (HDFs) and iPSCs. Boxplots indicate the distribution of the Spearman correlation coefficients of interaction signals among biological replicates (Ex. 1 vs. Ex. 2). **c** Analysis of chromatin interactions with 3D DNA FISH in iPSCs. The left panel illustrates the positions of positive (bivalent gene *LGI3* in PSCs) and negative (active gene *MTUS1* in PSCs) interaction target loci relative to the bait (bivalent gene *GATA4* in PSCs) locus on the genome. The bar graph in the right panel shows the colocalization percentage between the *GATA4* locus and the positive (magenta) or negative (green) interaction loci (*n* = 456, Fisher’s exact test, *p < 1 × 10^–8^). The right lower panel shows a nuclear image of 3D DNA FISH with Z-projection. Nuclei are stained with Hoechst (blue). The bait locus (*GATA4*), interaction-positive locus (*LGI3*), and interaction-negative locus (*MTUS1*) are indicated in white, magenta, and green, respectively. Scale bar, 5 μm. See also Supplementary Fig. 2 and Supplementary Tables 2 and 3

To investigate the chromatin interaction feature in bivalent gene loci, as the bait regions of ms4C-seq, we selected 29 bivalent, 31 active, 17 repressive, and 12 other genes from the gene groups in PSCs that we defined above (Fig. 1b; Supplementary Tables 1, 2). The 29 bivalent genes in PSCs include typical developmental regulators for ectoderm, mesoderm, and endoderm^32–35^. We performed ms4C-seq for all 89 bait loci on human iPSCs (hiPSCs; generated with episomal vectors), and for 42 bait loci out of 89 bait loci on their original HFs. Using the data from each bait that met our strict criteria for quality control (Supplementary Tables 2, 3; see also Methods), we created domainograms to visualize the interaction profiles^36, 37^ (Fig. 2a; Supplementary Fig. 2a). These profiles clearly demonstrated that ms4C-seq achieves high reproducibility between two independent biological replicates in HFs and hiPSCs. Moreover, the Spearman correlation coefficients for the chromatin interaction frequencies between biological replicates also confirmed the high reproducibility of ms4C-seq (Fig. 2b; Supplementary Table 4). We then assessed the results of ms4C-seq by 3D DNA fluorescence in situ hybridization (FISH) and confirmed the physical interactions at the single-cell level in both HFs and hiPSCs (Fig. 2c; Supplementary Fig. 2b). Furthermore, 3C-qPCR assays independently confirmed our ms4C-seq data, showing that the *HOXA13* locus frequently interacts with *HOXA3* and *HOXA5* in hiPSCs but not in HFs (Supplementary Fig. 2c). Taken together, our ms4C-seq data are highly reliable for analyzing the genome-wide interaction profiles of bivalent regions before and after cellular reprogramming.

### Chromatin structures reorganize at bivalent gene loci

**Fig. 3:**
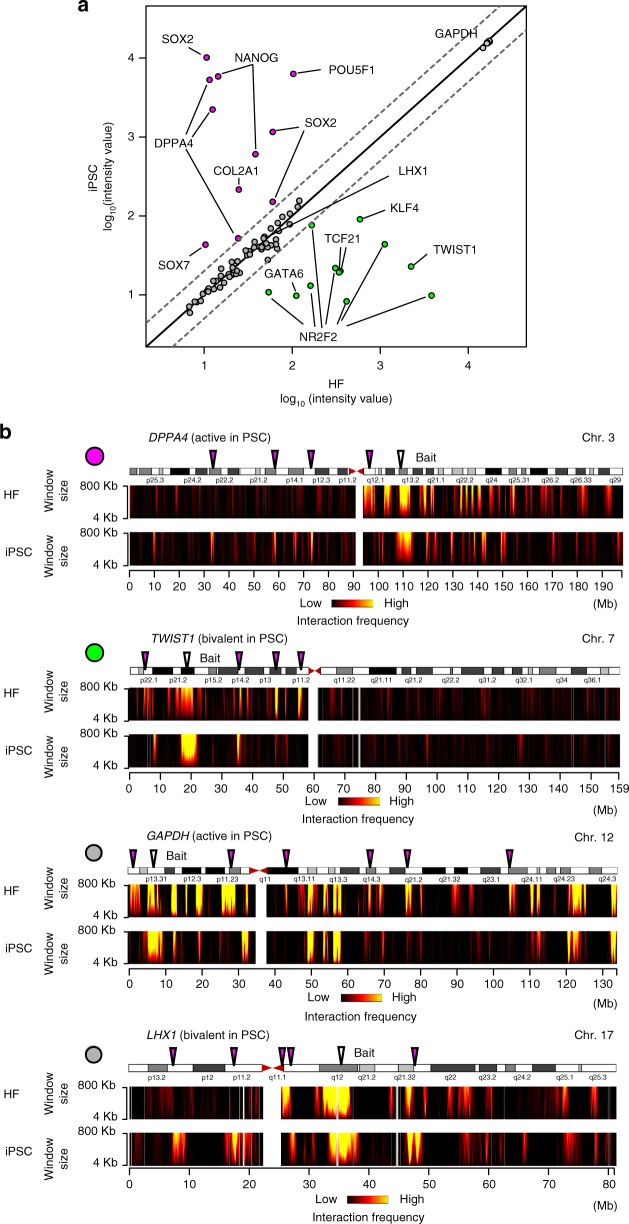
Chromatin interaction profiles at bivalent gene loci in somatic cells and hPSCs. **a** Expression profiles of bait genes in iPSCs and their original HFs (HDFs). The scatter plot represents the log_10_ signal intensity of probe sets for Affymetrix GeneChip Array (HG-U133_Plus_2). A single gene is sometimes represented by multiple probe sets corresponding to different isoforms and ESTs derived from the same gene locus. Thus, some genes have multiple dots (probe sets). Two dashed lines indicate the two-fold changes in gene expression levels between HFs (HDFs) and hiPSCs. The dots are color-coded as category 1 (magenta), category 2 (green), and category 3 (gray) on the basis of the fold change of the signal intensity. **b** *Cis*-chromosomal interaction profiles of the bait loci (*DPPA4*, *TWIST1*, *GAPDH*, and *LHX1*). The interaction frequencies of baits are shown by domainograms^37^. DPPA4 and GAPDH are active genes in PSCs. TWIST1 and LHX1 are bivalent genes in PSCs. The arrowheads indicate bait loci (white) and representative interaction regions that differ between HFs (HDFs) and hiPSCs (magenta). The left upper dots are colored according to the definition in **a**

During mouse iPSC induction, enhancer–promoter looping formation at the *Nanog* locus is reestablished before the genes are expressed^17^, possibly indicating that chromatin remodeling causes changes in gene expression. In order to investigate the relationship between chromatin structure and gene expression, we compared changes in the interaction profiles and gene expression profiles before and after hiPSC induction. The bait genes as viewpoints were divided into three groups: genes with higher (category 1), lower (category 2), and similar (category 3) expression in hiPSCs than HFs (Fig. 3a). We found that the interaction profiles for genes in all three categories dynamically changed before and after reprogramming (Fig. 3b; Supplementary Fig. 3). These results indicate that the chromatin interaction profiles of various bait gene loci are remodeled during somatic cell reprogramming regardless of changes in the expression at the bait genes.

### Reposition of bivalent gene loci by cellular reprogramming

**Fig. 4:**
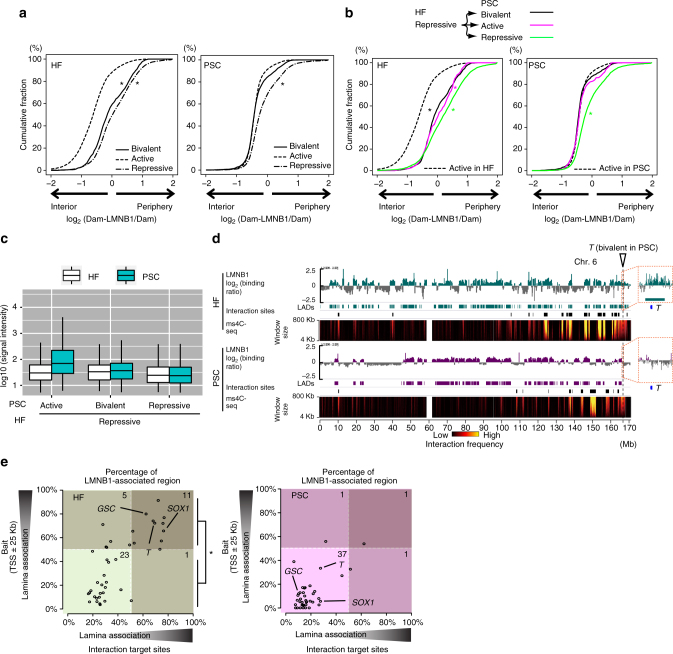
Nuclear positions at bivalent gene loci in somatic cells and hPSCs. **a** Cumulative distribution of the lamina-association signals (log_2_(Dam-LMNB1/Dam) values^39, 40^) at active (dashed line), repressive (dash-dot line), and bivalent (solid line) gene loci in HFs and PSCs (ESCs). *p < 3.0 × 10^−16^ for Mann–Whitney *U*-test using active genes for the comparisons. **b** Cumulative distribution of the lamina-association signals (log_2_ (Dam-LMNB1/Dam) values^40, 41^) for the genes which change their states from repressive in HFs to active (magenta), repressive (green), or bivalent (black) in PSCs (ESCs). *p < 3.0 × 10^−16^ for Mann–Whitney *U*-test using active genes for the comparisons. **c** Comparison of expression values for the indicated gene groups in iPSCs and their original HFs (HDFs). The boxplots indicate the distribution of log_10_ (signal intensity) values. The box plots are colored by cell types. **d** The relationship between chromatin interaction profiles and lamina-association profiles at *T* gene bait loci in HFs and hPSCs. The positive log_2_(Dam-LMNB1/Dam) signals^40, 41^ are indicated by the green (HFs) or magenta (PSCs (ESCs)) bar chart above the HF and PSC tracks. Positive log_2_(Dam-LMNB1/Dam) signals indicate lamina-associated regions. Rectangles below each signal track depict lamina-associated domains. The interaction frequencies determined by ms4C-seq in HFs (HDFs) and iPSCs are shown as described in Fig. 2a. **e** The subnuclear localization of the bait loci and their interaction target loci in HFs (top) and PSCs (iPSCs, bottom). Scatter plots present the strength of the lamina association of the bait gene loci (TSS ± 25 kb) (*y*-axis) and their chromatin interaction target sites (*x*-axis) (*U*-test, *p < 3 × 10^−7^). Bivalent baits indicating *GSC*, *T*, and *SOX1* loci are shown as representative bait loci with interaction target loci located in the nuclear periphery in HFs and the nuclear interior in PSCs (ESCs)

Pluripotency-related gene loci change their nuclear positions between the periphery and interior during the differentiation of mouse ESCs^38^, and this repositioning is important for transcriptional control^39^. However, little is known about the nuclear chromatin repositioning where the bivalency was restored in the reprogramming process. To investigate the subnuclear localization of bivalent gene loci in HFs and PSCs, we examined the distribution of lamina-association signals in active, repressive, and bivalent gene loci in HFs and PSCs using publically available data sets of LaminB1 DamID signals^40, 41^ as a surrogate for peripheral localization, which was confirmed by DNA FISH experiments^38^. Consistent with previous studies that reported the nuclear localizations of gene loci are closely related to transcription (active genes in the nuclear interior and inactive genes in the nuclear periphery)^39, 42, 43^, we observed that active and repressive genes were biased to the nuclear interior and periphery, respectively, in both HFs and PSCs (Fig. 4a). Notably, while bivalent genes in HFs tended to localize to the nuclear periphery, bivalent genes in PSCs were biased to the nuclear interior, suggesting that the regulatory mode of the nuclear localization for bivalent genes in HFs differs from those in PSCs. Next, we examined the relationship between the changes of chromatin states and those of nuclear localization and found that when the gene states are changed from repressive in HFs to active in PSCs, the distribution of the lamina-association signals becomes biased to the nuclear interior in PSCs (Fig. 4b, magenta lines). Moreover, the gene loci whose states changed from repressive in HFs to bivalent in PSCs behaved similarly to those changed to active in PSCs (Fig. 4b, black and magenta lines), even though bivalent genes were not transcriptionally activated in PSCs (Fig. 4c). These results suggested that repressed genes in HF changed their position from the nuclear periphery toward the interior in hPSCs not only when they became active but also when they became bivalent. Our data also showed that when bivalent loci as baits changed their position from the nuclear periphery of HFs to the interior of hPSCs, the interaction target regions of the baits likewise changed (Fig. 4d, e; Supplementary Fig. 3). These results imply that the reestablishment of 3D chromatin structures is accompanied by the reorganization of chromatin states and chromatin repositioning in the nucleus before and after cellular reprogramming.

### Bivalent gene loci colocalize in pluripotent stem cells

**Fig. 5:**
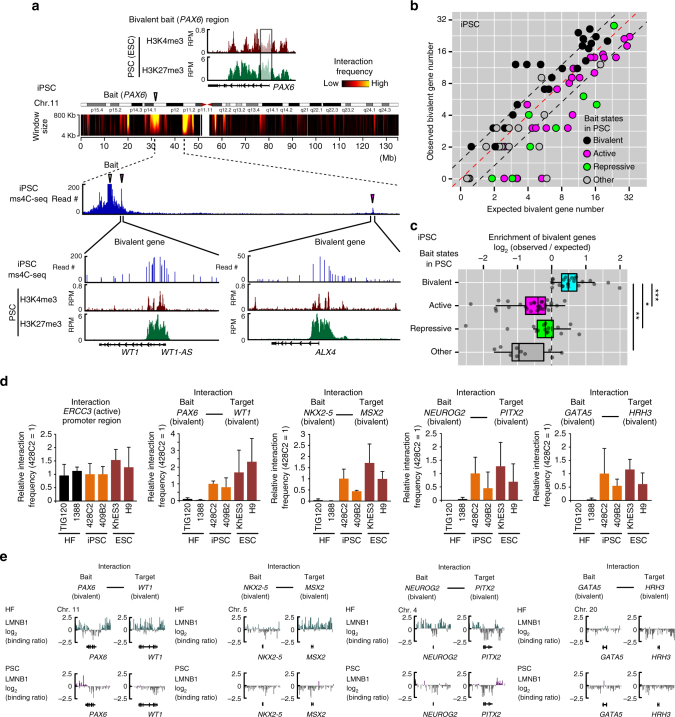
Bivalent gene loci colocalize in hPSCs. **a** The distribution of histone modifications for H3K4me3 and H3K27me3 in the *PAX6* bait locus and their target loci. ChIP-seq data are represented by RPM signals for H3K4me3 and H3K27me3 in ESCs below ms4C-seq interaction signals in iPSCs. **b** Bivalent gene numbers in the interaction target genes of active, repressive, and bivalent bait loci in iPSCs (428C2). The scatter plot shows the expected numbers of bivalent gene (*x*-axis) and observed numbers of bivalent genes (*y*-axis) in the interaction targets of each bait locus. The expected number was calculated by the proportion of bivalent genes present on the same chromosome as bait loci. Bait genes were divided into bivalent (black), active (magenta), repressive (green), and other (gray) in PSCs (ESCs) according to Fig. 1b. **c** The enrichment of bivalent genes in interaction target genes of bivalent bait loci in iPSCs (428C2). Box plots show log_2_(observed frequency for bivalent genes)/(expected frequency for bivalent genes) in interaction targets of bivalent, active, repressive, and other bait gene loci (*U*-test, *p < 3 × 10^−5^, **p < 1 × 10^−5^, and ***p < 1 × 10^−11^). **d** Interaction frequencies of bivalent gene loci in hESCs (KhES3 and H9), hiPSCs (428C2 and 409B2), and their original HFs (TIG120 and 1388). Bar graphs indicate the interaction frequencies of the indicated gene loci determined by enhanced 3C-qPCR analysis. The interaction frequencies in the *ERCC3* promoter region are shown as positive interaction controls. Data are shown as means ± SD from three biological replicates. **e** Lamina-association profiles of colocalized bivalent gene loci are indicated by log_2_(Dam-LMNB1/Dam) in HFs and PSCs (ESCs)^40, 41^. See also Supplementary Figs. 4–6

Transcriptionally active gene loci are known to colocalize^44–46^ with one another. Because many active genes are marked by H3K4me3 in their promoter regions^3, 47^, genomic regions with histone modification H3K4me3 may be expected to colocalize. In fact, our ms4C-seq data also showed that pluripotency-related genes such as *NANOG*, *DPPA4*, and *SOX2* (active genes in PSCs) interact with genomic regions with the H3K4me3 modification (Supplementary Fig. 4). To investigate the histone modification profiles around interaction targets of bivalent bait genes in PSCs, we compared our ms4C-seq data with a genome-wide data set of various histone modifications in hPSCs. Notably, we found that several interaction regions of bivalent bait loci often overlapped with the promoter regions of other bivalent genes (Fig. 5a; Supplementary Fig. 5). For example, in hiPSCs, the *PAX6* bait region interacted with bivalent domains, including the promoters of *WT1* and *ALX4* (Fig. 5a). These observations indicate that bivalent genes localized together in hPSCs. To further support this notion, we calculated the enrichment of bivalent genes in the interaction regions from the perspective of the bivalent bait loci. Our data demonstrated that bivalent genes are enriched in the interaction targets of bivalent genes, but not in those of active or repressed genes (Fig. 5b, c). Importantly, while significant enrichments of active and repressive genes were observed in the interaction targets of active and repressive gene loci (baits), respectively (Supplementary Fig. 6a), no enrichment of active or repressive genes was observed in the interaction targets of bivalent gene loci (baits) (Supplementary Fig. 6a). These data suggested that bivalent genes preferentially interact with bivalent genes, but neither with active nor repressive genes. The colocalization of bivalent gene loci was also observed in single-cell analysis by 3D DNA FISH (Fig. 2c). To examine whether the colocalization of bivalent gene loci is true for other hPSC lines, we investigated the interaction frequencies of bivalent gene loci defined in PSCs by 3C-qPCR. In accordance with the results of the genome-wide analysis by ms4C-seq in hiPSCs, bivalent gene loci colocalized with one another in hPSCs in the nuclear interior (Fig. 5d, e). Together, our results strongly suggest that bivalent gene loci frequently colocalize in the nuclear interior of hPSCs.

Approximately 25% of bivalent genes are DNA methylation valley (DMV) genes^48^, which contain large hypomethylated regions. In addition to the above results, we observed that bivalent genes interacted with DMV genes (Supplementary Fig. 6b, c), suggesting that epigenetic regulation including histone modification and DNA methylation may contribute to the organization of higher-order chromatin structures in hPSCs.

### Bivalent gene colocalization requires PcG and TrxG proteins

**Fig. 6:**
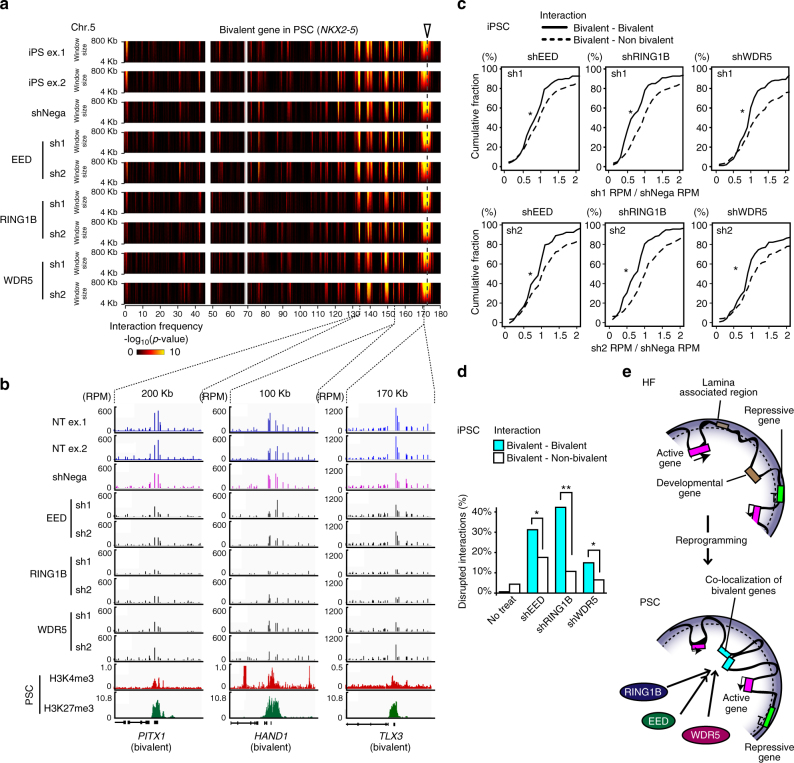
EED, WDR4, and RING1B are important for the colocalization of bivalent gene loci. **a** Interaction profiles determined by ms4C-seq for the *NKX2-5* gene locus in EED, WDR5, and RING1B KD iPSCs. Domainograms indicate *cis*-chromatin interaction frequencies at the *NKX2-5* gene locus on an Mb scale in shRNA-treated iPSCs. **b** The disruption of chromatin interactions between bivalent gene loci by shRNA treatment. Bar plots indicate interaction signals (RPM) at interaction target gene loci of the *NKX2-5* gene bait locus. ChIP-seq signals (RPM) for H3K4me3 and H3K27me3 histone modifications in PSCs (ESCs) are shown under the ms4C-seq interaction signals. Reduced interaction signals of the bait *NKX2-5* gene locus are detected in bivalent gene loci (*PITX1*, *HAND1*, and *TLX3*) after shRNA treatment against EED, WDR5, or RING1B in PSCs (iPSCs). **c** Cumulative distribution of reduction rates for interaction signals (RPM values in TSS ± 25 kb) in bivalent–bivalent (solid line) and bivalent–nonbivalent (dashed line) interactions by two independent knockdown (two different shRNAs) experiments for EED, RING1B, or WDR5 in PSCs (iPSCs). *p < 0.03 was assessed for bivalent–bivalent interactions vs. bivalent–nonbivalent interactions by two-sided Mann–Whitney *U*-test. **d** Disruption of bivalent gene colocalizations by the KD for EED, WDR5, or RING1B. Bar graphs show the percentages of colocalized bivalent genes (light blue) and nonbivalent genes (white) with interaction signals (RPM values in TSS ± 25 kb) that were reduced to at least 30% following both sh1 and sh2 treatments for EED, WDR5, and RING1B compared to shNegative treatment (Fisher’s exact test, *p < 0.01, **p < 1 × 10^−6^). **e** A model for the changes in chromatin interactions and subnuclear localization around bivalent gene loci during somatic cell reprogramming. See also Supplementary Fig. 7

Histone modifiers, such as PRC1, PRC2, and TrxG, contribute to the maintenance of the poised state of bivalent gene loci in PSCs^6–8, 11^. To obtain mechanistic insights into the colocalization of bivalent gene loci, we focused on PRC1, PRC2, and TrxG protein complexes. We introduced small hairpin RNAs (shRNAs) that target RING1B, EED, and WDR5, which are components of the PRC1, PRC2, and TrxG complexes, respectively, into hiPSCs and performed ms4C-seq for eight selected bivalent bait loci to examine the effects of the shRNAs on the interaction profiles (Supplementary Fig. 7a). We confirmed that the expression of each protein targeted by the shRNAs was reduced by 50–90% (Supplementary Figs. 7b, 8). A large reduction in interaction frequency was not observed in any of the shRNA-treated samples at the resolution used in the domainogram analysis, indicating that the chromatin structures were largely maintained after the knockdown of EED, WDR5, or RING1B (Fig. 6a). In contrast, we noticed that normalized signals of ms4C-seq at several gene loci were significantly decreased after knockdown of the target genes (Fig. 6b). We examined the reduction rates of the interaction frequencies by shRNA knockdown experiments (shRNA/shNegative RPM) in the interaction target gene loci of bivalent bait gene loci and found that reduction rates associated with bivalent gene loci were lower than those associated with nonbivalent genes in independent sh1 and sh2 knockdown experiments for EED, WDR5, and RING1B with statistical significance (Fig. 6c). Then, we tried to identify the target genes whose interaction frequencies were reduced by more than 30% in both independent knockdown experiments compared with negative control. Our results showed that many bivalent colocalization events were significantly disrupted by the knockdown of EED, WDR5, or RING1B compared to interactions with nonbivalent genes although the degree of the effects varied in each bait (Fig. 6d), and the knockdown effect was different depending on the bait (Supplementary Fig. 7c). These results led us to conclude that both PcG and TrxG proteins are important for the colocalization of bivalent gene loci, but that the effects are variable.

## Discussion

In this study, we describe ms4C-seq using Splinkerette adaptor^23^, a 4C-based method that integrates the advantages of multiplexed PCR amplification^21^, adaptor-mediated PCR amplifications^17^, and sonication-based random DNA fragmentation^24^. ms4C-seq was able to reliably and simultaneously identify chromatin interactions at multiple bait loci in HFs and hiPSCs, while limiting PCR duplication bias (Fig. 2a, b; Supplementary Fig. 2a–c). Although the *cis* interactions identified in this study by ms4C-seq showed high reproducibility in biological duplicate experiments, the *trans* interactions did not (Supplementary Table 4). This poor reproducibility in *trans* interactions may have been due to spurious random ligation events among intermolecules in the in-solution ligation step of the ms4C-seq preparation. Therefore, ms4C-seq may be improved by performing in-nucleus ligation in the 3C library preparation^49^. We also observed a certain level of nonspecific amplification and unequal distribution of the number of sequenced reads among baits in the ms4C-seq data. These inefficiently sequenced libraries may be amended by optimizing the primer sequences of the first and second PCR used to construct the ms4C-seq library. After the completion of our work, the UMI-4C method, which is similar to ms4C-seq, was reported^50^, supporting the general utility of these methods for examining chromatin interactions.

The subnuclear localization of gene loci affects gene expression. Inactive genes marked by transcriptionally silent histone modifications, such as H3K27me3 and H3K9me3, are enriched in the nuclear periphery, whereas transcriptionally active genes marked by H3K4me3 are localized in the nuclear interior^41, 51, 52^. Furthermore, the artificial tethering of a gene to the nuclear membrane leads to repression of the active gene^39^, strongly supporting a causal relationship between nuclear positioning and gene expression. Our analyses showed that, along with the restoration of bivalency, gene loci at the nuclear periphery in HFs are located in the nuclear interior of hPSCs, and that the bivalent gene repositioning is accompanied by alterations in the nuclear position of the interaction target loci (Fig. 4; Supplementary Fig. 3). These results suggest that developmental gene loci might change their nuclear position from the nuclear periphery to the interior during somatic cell reprogramming, allowing the loci to be easily accessible to transcription factors and RNA polymerase in the nuclear interior of hPSCs.

In the nuclear interior, active genes colocalize in a distinct structure, termed the transcriptional factory, which may be important for the coregulation of gene expression^44–46^. In contrast, our results demonstrated that bivalent gene loci frequently colocalize in hPSCs (Fig. 5; Supplementary Fig. 6a), even though their expression was hardly observed. Similar to the concept of the transcriptional factory, the colocalization of bivalent gene loci may be crucial for the coregulation of developmental genes in hPSCs. By sharing regulatory factors, including transcription factors and RNA polymerase, the colocalization of bivalent gene loci in hPSCs may allow developmental genes to be coordinately transcribed as a rapid response to extracellular differentiation cues.

We also showed that histone modifiers contribute to the colocalization of bivalent genes in hPSCs. RING1B and EED, which are components of PRC1 and PRC2, respectively, are essential for the chromatin organization of polycomb-regulated gene loci in mouse PSCs^53–55^. Our results suggest that PRC1 and PRC2 are involved in broader aspects of chromatin interactions including bivalent gene colocalization. We also revealed that an active histone modifier, WDR5, is required for higher-order chromatin structures in hPSCs (Fig. 6; Supplementary Fig. 7c). Recent studies have indicated that noncoding RNAs are important for the construction of chromatin interactions^56, 57^. Interestingly, several enhancer-like long noncoding RNAs (lncRNAs) also play a role in recruiting WDR5 to target loci for the methylation of H3K4^58–60^. Therefore, in addition to histone modifiers, lncRNAs may also mediate the colocalization of bivalent genes.

Our knockdown experiments showed that the contribution of RING1B, EED, and WDR5 to the colocalization of bivalent genes varies among baits. These different effects on the colocalization of bivalent gene loci may have several causes, including different cofactors, binding strengths, frequencies, and stabilities of the histone modifiers. Knockout of RING1B and EED derepresses developmental genes in ESCs^11, 61^, and WDR5 is required for ESC self-renewal^62^. Therefore, the colocalization of bivalent genes that are mediated by histone modifiers may be a critical regulatory event underlying the differentiation and self-renewal capabilities of PSCs.

In conclusion, our present study shows that bivalent gene loci colocalize to the nuclear interior of hPSCs, and that the organization of chromatin 3D structures around bivalent genes is mediated by histone modifiers related not only to inactive but also to active marks (Fig. 6e). Because bivalent histone modifications increase during the transition from the morula to the inner cell mass or trophectoderm in early embryogenesis^63^, the detailed regulatory mechanisms of higher-order chromatin structures and subnuclear localization at bivalent gene loci should provide insights on early embryogenesis.

## Methods

### Cell culture conditions

hESC lines (H9 and KhES3) and hiPSC lines (428C2 and 409B2)^64, 65^ were cultured in DMEM/F12 (Thermo Fisher 10565-018) supplemented with 20% knockout serum replacement (Thermo Fisher 10828-028), 2 mM l-glutamine (Thermo Fisher 25030-081), 1× nonessential amino acids (Thermo Fisher 11140-050), 110 mM 2-mercaptoethanol (Thermo Fisher 21985-023), penicillin/streptomycin (0.05 U, 50 µg per ml) (Thermo Fisher 15140-122), and 4 ng per ml of hbFGF (Wako 064-054541) on feeder layers of mitomycin-C-treated SNL-STO cells stably expressing the puromycin resistance gene. HFs (1388 and TIG120) were cultured in DMEM (Nacalai Tesque 08459-64) supplemented with 10% FBS (Japan Bio Serum S1560-500). The mouse SNL-STO cells used as feeder cells were cultured in DMEM (Nacalai Tesque 08459-64) supplemented with 10% FBS (Thermo Fisher 10437-028), 0.05 U per ml of penicillin, and 50 µg per ml of penicillin/streptomycin (0.05 U, 50 µg per l) (Thermo Fisher 15140-122). All cells were cultured at 37 °C in a 5% CO_2_ incubator. KhES3 was obtained from Kyoto University. H9 was from WiCell Research Institute. TIG120 was obtained from the Japanese Collection of Research Bioresources. HDF 1388 was purchased from Cell Applications.

### Vector construction

shRNA expression vectors were constructed by cloning the hU6 promoter and puromycin resistance genes between piggyBac-inverted terminal repeats^66^. Target sequences of shRNAs for EED, RING1B, and WDR5 were designed using siDirect (http://sidirect2.rnai.jp/) and cloned downstream of the hU6 promoter of the modified vector (Supplementary Fig. 7a; Supplementary Table 1). The CAG promoter-driven expression vector of piggyBac transposase^67^ was cotransfected with the shRNA expression vector.

### Knockdown experiments

428C2 cells were washed with 1× PBS, and feeder cells were removed by 3–5 min of incubation in dissociation solution (Repro Cell RCHETP002). The hiPSC colonies were dissociated into single cells by incubation in ACCUMAX (Innova Cell Technologies AM105) at 37 °C for 10 min. To prepare the transfection mix, 6 μg of shRNA expression vector and 6 μg of CAG promoter-driven expression vector of piggyBac transposase were mixed with 540 μl of Opti-MEM (Thermo Fisher 31985-062) and 46 μl of FuGENE HD (Promega E2311). After incubation of the transfection mix at RT for 15 min, 5 × 10^6^ cells were resuspended in the transfection mix and incubated at RT for 5 min. shRNA-transfected cells were suspended in 6 ml of mTeSR1 medium (Stemcell Technologies 05850) in the presence of 10 μM Y-27632 (Wako 257-00511) on Matrigel (CORNING 356231)-coated 10-cm dishes. Selection with 2 μg per ml puromycin was performed 2 days after transfection. The cells were harvested 4 days after transfection for ms4C-seq analysis, immunoblotting, and quantitative PCR (qPCR) analysis.

### RNA isolation, cDNA synthesis, and qPCR

RNA was extracted from each cell line with RNeasy Mini Kit (QIAGEN 74104). Complementary DNA was synthesized from 100 ng of total RNAs with QuantiTect Reverse Transcription Kit (Qiagen). qPCR analysis was performed with SYBR premix Ex TaqII (Takara) and primer sets (Supplementary Table 1) on StepOnePlus Real-Time PCR System (Applied Biosystems). Expression levels were normalized to that of GAPDH.

### Microarray experiments

RNA was extracted from hiPSCs (409B2) and the original HFs (1388) with TRIzol and QIAGEN RNeasy Kit. The total RNA was subjected to cRNA synthesis with 3′ IVT Express Kit (Affymetrix), and the resultant cRNA was fragmented and hybridized to the HG-U133 Plus 2 platform (Affymetrix). After hybridization, GeneChip arrays were washed, stained with GeneChip Fluidics Station 450 (Affymetrix), and detected with Scanner 3000 TG System (Affymetrix) following the manufacturer’s standard protocols. Data analyses were performed with GeneSpringGX 13.0 software (Agilent Technologies).

### Immunoblotting

Cell lysates were prepared from an equal number of cells (0.5–1 × 10^6^ cells), which were suspended in 100 μl of CytoBuster Protein Extraction Reagent (BIO RAD 71009-50ML) supplemented with 1× protease inhibitor cocktail (Roche 04 693 159 001) followed by the addition of 100 μl of Laemmli sample buffer (Bio-rad 161-0737) in the presence of 5% 2-mercaptoethanol (Nacalai 21438-82). SDS–PAGE was performed with 10% gels (Bio-rad 456-1036) or gradient gels (Bio-rad 456-9036) in 1× Tris/Glycine/SDS buffer (Bio-rad 161-0732). The separated proteins were transferred to a PVDF membrane (Bio-rad 162-0174). Target proteins were detected with the following antibodies: anti-EED (1:1000; rabbit; Millipore 09-774), anti-WDR5 (1:1000; rabbit; Millipore 07-706), anti-RING1B (1:500; rabbit; Cell Signaling Technology D22F2), and anti-GAPDH (1:4000; Ambion AM4300). Secondary antibodies were Anti-Rabbit IgG, HRP-Linked F(ab′)2 Fragment Donkey (1:4000; NA9340; GE Healthcare Life Science), or Anti-Mouse IgG, HRP-Linked F(ab′)2 Fragment Sheep (1:4000; NA9310; GE Healthcare Life Science).

### 3D DNA FISH

Probes were prepared from BAC clones (BACPAC Resources) by using a Nick Translation Reagent Kit (Abbott Molecular Inc. 32-801300) and labeled with Red 650 dUTP (ENZO ENZ-42522), Red 580 dUTP (ENZO ENZ-42844), or Green 496 dUTP (ENZO ENZ-42837). Nick-translated probes were precipitated with 70% EtOH (1 µg/µl human CotI DNA, 10 µg per μl of salmon sperm DNA, and 3 M sodium acetate). The probes were dissolved in 4 µl of formamide that was preheated at 42 °C, denatured at 75 °C for 10 min, and then placed on ice for 3 min. The denatured probes were dissolved in 1× hybridization buffer (2 mg per ml of BSA (Roche 711454), 2× SSC, and 10% dextran sulfate sodium salt (Sigma D8906)). Cells cultured on gelatin-coated chamber slides (Iwaki) were fixed with 4% PFA in PBS at room temperature for 10 min and permeabilized with 0.5% Triton X-100 in PBS at RT for 5–15 min. Permeabilized cells were washed two times with 70% EtOH that was prechilled at −20 °C and dehydrated in 70, 90, and 100% EtOH (v per v) at RT for 5 min each. DNA in the nuclei was denatured in buffer (70% formamide (pH 7.0), 2× SSC) at 80 °C for 5 min. The denatured samples were washed and dehydrated with a gradient of ice-cold ethanol (70, 90, and 100% EtOH (v per v)) for 5 min each. The dissolved probes were hybridized to the samples in a 37 °C wet box for 1–2 days. The samples were then washed three times with 50% formamide/2× SSC solution at 42 °C for 10 min and then washed two more times with 2× SSC solution at 42 °C for 5 min. The nuclei were counterstained with 2 µg per ml of Hoechst at RT for 10 min. FISH signals were detected by using Zeiss LSM710. Z sections that were captured at 0.2-µm intervals. The following BACs were used in these studies: RP11-235I5 (*GATA4*), RP11-102O8 (*LGI3*), RP11-383B10 (*MTUS1*), RP11-1083M23 (*TWIST1*), RP11-815K24 (*EGFR*), and RP11-614D15 (*INHBA*). Distances between the bait probe and the positive or negative probes were measured in 180–456 signal pairs from three to five visual fields in a single experiment and the number of colocalized signals was counted. The statistical significance was calculated by Fisher’s exact test.

### 3C library preparation and 3C-qPCR assay

The 3C assays in this study were performed according to the following protocol^22^. Briefly, 1–2 × 10^7^ cells were cross-linked in 9.25 ml of ES cell culture medium containing 1% formaldehyde (Wako 064-00406) at RT for 10 min. The formaldehyde was quenched with 500 μl of 2.5 M glycine and washed twice with 5 ml of ice-cold 1× PBS. Cross-linked nuclei were isolated by incubation in 550 μl of lysis buffer (10 mM Tris-HCl, pH 7.5, 10 mM NaCl, 0.2% Np-40, and 1× protease inhibitor (Roche 04 693 159 001)) on ice for 20 min and homogenization with 10 strokes × 2 by a tight pestle in a dounce homogenizer (Wheaton) on ice. The isolated nuclei were washed twice with 500 μl of 1× cutsmart buffer (NEB B7200S). The formaldehyde was quenched with 500 μl of 2.5 M glycine and washed twice with 5 ml of ice-cold 1× PBS. Cross-linked nuclei were isolated by incubation in 550 μl of lysis buffer (10 mM Tris-HCl, pH 7.5, 10 mM NaCl, 0.2% Np-40, and 1× protease inhibitor (Roche 04 693 159 001)) on ice for 20 min and homogenization with 10 strokes × 2 by a tight pestle in a dounce homogenizer (Wheaton) on ice. The isolated nuclei were washed twice with 500 μl of 1× cutsmart buffer (NEB B7200S). Proximity ligation of the *Hin*dIII-digested DNA ends was conducted with 10 U (Weiss unit) of T4 DNA ligase (TAKARA 2011A). 3C DNA libraries were purified by phenol–chloroform extractions (Nacarai 25970-56, 26829-96) and ethanol precipitation. Precipitated 3C libraries were purified again by using an Invitrogen PureLink Genomic DNA Mini Kit (Thermo Fisher K1820-00). The 3C-qPCR experiments were performed by using a SYBR premix Ex TaqII (Takara) and primer sets (Supplementary Table 1) on a StepOnePlus Real-Time PCR System (Applied Biosystems). For qPCR, 100 ng of template was used for each reaction, and the reaction steps were 1 cycle at 95 °C for 30 s and 45 cycles at 95 °C for 5 s, and 1 cycle at 60 °C for 30 s. Interaction frequencies were normalized to the DNA amount based on measurements using the GAPDH locus.

### ms4C-seq and library construction for enhanced 3C-qPCR

The 3C libraries were sonicated into 300–700-bp-long fragments in microtubes (Covaris 520045) with Covaris E210 under the following conditions: duty cycle 5%, intensity 3, cycles per burst 200, and time 150 s. End-repairing and A-tailing were performed by using NEBNext Ultra End Repair/dA-Tailing Module (NEB E7442S) according to the manufacturer’s instructions. Ligation of the Splinkerette adaptor^23^ for sequencing by the next-generation sequencer (Top: 5′-CGAAGAGTAACCGTTGCTAGGAGAGACCGTGACTGGAGTTCAGACGTGTGCTCTTCCGATCT-3′, Bottom: 3′-phosphate-GATCGGAAGAGCTGTTTTTTTTTTCAAAAAAA-5′) to the A-tailed 3C libraries was performed with Ligation high Ver.2 (TOYOBO LGK-201) at 16 °C for 30 min and 10:1 adapter:library molar ratio. The adaptor-ligated 3C libraries were purified with 1.8× AMPureXP beads. A total of 3.7 μg (230 ng/reaction × 16) of adaptor-ligated 3C libraries served as the template to amplify target loci by PCR amplification. Each PCR was performed in a 50-μl reaction mix (1× Phusion High-Fidelity PCR Master Mix with HF Buffer (NEB M0531S), 50 nM each universal and target primers (Supplementary Table 1), and 230 ng/tube template). The reaction steps were 1 cycle at 98 °C for 30 s, 20 cycles at 98 °C for 10 s, 62 °C for 30 s, and 72 °C for 30 s; and 1 cycle at 72 °C for 10 min. Samples after the first PCR amplification were also used for 3C-qPCR assays as templates of enhanced 3C-qPCR (Fig. 3d). To reduce the influence of different amplification efficiencies among primer sets, a second nested PCR was performed in the 13 bait groups, and it had similar amplification efficiencies (data not shown). The reaction steps for the second PCR were 1 cycle at 98 °C for 30 s, 20 cycles at 98 °C for 10 s, 60 °C for 30 s, and 72 °C for 30 s; and 1 cycle at 72 °C for 10 min (second universal and target primers (Supplementary Table 1). Amplified multiplexed splinkerette 3C libraries were electrophoresed on a 2% agarose gel (Bio-Rad 1613107), and libraries between 350 and 700 bp were excised and purified with QIAquick Gel Extraction Kit (QIAGEN 28704). This library was quantified with Library Quantification Kit (KAPA KK4824) and sequenced via HiSeq 2000 sequencing with paired-end reads of 100 bp from both ends or NextSeq 500 with paired-end reads of 80 bp from both ends. For the *POU5F1* locus, we were unable to design a specific second primer (second universal and target primers (Supplementary Table 1)), because its sequence is present in three loci: chr. 6, chr. 12, and chr. 1. However, we used this second primer to amplify the *POU5F1* locus, because the first PCR primer for *POU5F1* is specific to the bait locus.

### Analysis of ms4C-seq data

ms4C-seq data sets were analyzed according to the following procedures. Bases with low-quality scores and the adapters in all sequenced reads were trimmed with Cutadapt-1.4.2^68^. Read pairs with the *Hin*dIII site “AAGCTT” in the read1 (R1) sequence were used for the following analysis. The R1 sequences were separated into bait sequences and target sequences. To divide the reads into each bait, the former parts from *Hin*dIII of R1 were excised as bait sequence and mapped to 47 bait sequences by BWA (bwa-0.6.2)^69^ by using the default setting. The *NANOG* pseudogene gene loci were discriminated from *NANOG* bait loci on the basis of an SNP in the *NANOG* region. The latter parts from *Hin*dIII of the R1 and R2 read pairs were used for the subsequent mapping. These read pair sequences were mapped to the human reference genome (hg19) by BWA (bwa-0.6.2). The read pairs, which were derived from self-ligation and no-digestion events, were removed. PCR duplicates were removed by Picard (Picard-tools-1.97). The total read numbers within 1000 bp from each *Hin*dIII site were counted, and the counted numbers in the *Hin*dIII sites were collected as wiggle (.wig) format files. The total read numbers and the ratio of *cis*:*trans* reads were used for quality control (Supplementary Table 2). To exclude the baits, which have insufficient sequenced reads for further analysis, percentages of the observed *cis* interactions in the expected total number of *cis* interactions were calculated for each bait. The total number of distinct *cis* interactions was estimated by using Preseq software^70^.

### Domainograms and calculation of interaction frequencies

Using the read count data at each *Hin*dIII site (wiggle format data), we analyzed the contact probabilities of the *Hin*dIII site by the following method^53^. In our analysis, instead of running windows for the number of *Hin*dIII sites, running windows for distances from each *Hin*dIII site were used to obtain contact probabilities. The enrichment level of reads in small windows (within 100 Kb from the *Hin*dIII) for reads in large windows (within 3 Mb from the *Hin*dIII) was statistically calculated as contact probabilities in each *Hin*dIII. Two statistical methods, the *U*-test and the binomial test, were used to calculate contact probabilities in proximal regions (within 8-Mb regions) and the distal region (distance >8 Mb) from the baits, respectively. The contact probabilities in each *Hin*dIII site are presented in domainograms by using previously published programs^37^. Gene-to-gene contact probabilities were calculated around the TSS of each gene through the same method described above with the one-sided binomial test, and adjusted by FDR.

### Correlation analysis of chromatin interactions

To compare chromatin interaction profiles between biological duplicates or cell types, the following analysis was performed^53^. Briefly, wiggle-formatted ms4C-seq data in each *Hin*dIII were binarized to 1 (reads >0) and 0 (read = 0), and *cis*-hit coverages were calculated by averaging these binarized values in a sliding window (100 kb from each *Hin*dIII site). Spearman correlation coefficients were calculated by comparing the *cis*-hit coverages between cell types or biological duplicates.

### Definition of transcriptional gene states

Chromatin states data of hESCs and NHLFs were obtained from the ENCODE project data sets of UCSC (http://hgdownload.cse.ucsc.edu/goldenpath/hg19/encodeDCC/wgEncodeBroadHmm/wgEncodeBroadHmmH1hescHMM.CNV.bed, http://hgdownload.cse.ucsc.edu/goldenpath/hg19/encodeDCC/wgEncodeBroadHmm/wgEncodeBroadHmmNhlfHMM.bed.gz)^29^ and used for the gene definitions. We defined the refseq genes that are mainly occupied by “poised promoter”, “Active/Weak Promoter”, and “Repressed/Heterochrom” chromatin states as “bivalent genes”, “active genes”, and “repressive genes”, respectively.

### Analysis of LADs

For nuclear localization analysis of gene loci, we used publically available log_2_(Dam-LMNB1/Dam) signal data of Tig3 HFs^41^ (GSE8854) as HFs and of SHEF-2 human ESCs^40^ (GSE22428) as PSCs, and define the lamina-associated domains (LADs) according to a previously reported method^41^. Briefly, for the identification of LADs, the log_2_(Dam-LMNB1/Dam) signal was binarized with positive values to 1 and negative values to −1. Transition sites of these signals were identified as left and right side edges of LADs by the sliding filter, which compared the average binary values between the right and left 99-probe windows of the probe^40, 41^. Cumulative fraction analysis for log_2_(Dam-LMNB1/Dam) signals in each gene was performed using the average log_2_(Dam-LMNB1/Dam) values for each probe in the gene bodies.

### Data availability

All DNA microarray data are deposited in Gene Expression Omnibus under the accession number for GSE90141.

All ms4C-seq data are deposited in Gene Expression Omnibus under the accession number for GSE90782.

## Electronic supplementary material

Supplementary Information
