# Supplementary material for: Structural and spatial chromatin features at developmental gene loci in human pluripotent stem cells
Source: Nat Commun. 2017 Nov 20;8:1616. doi: 10.1038/s41467-017-01679-x (PMC5696376; doi:10.1038/s41467-017-01679-x)
Supplement: Supplementary file 1 — Supplementary Information [file 41467_2017_1679_MOESM1_ESM.pdf]

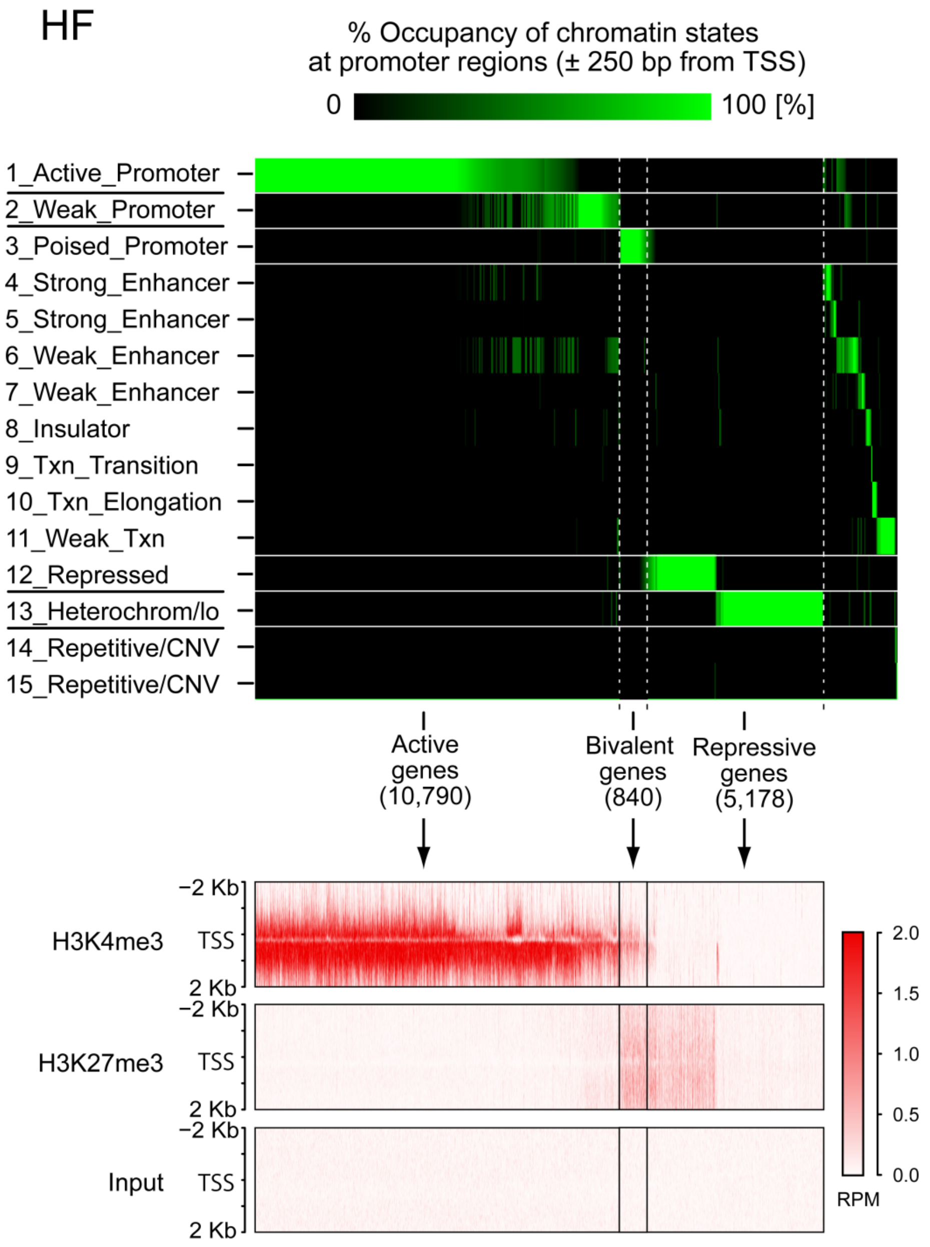

Supplementary Figure 1. Definition of transcriptional states of genes in HFs

Definition of transcriptional states of genes (bivalent, active, repressive and other gene groups). Top heat map represents the occupancy of each chromatin state (%) within TSS ± 250 bp for all refseq genes in HFs (NHLFs). Bottom heatmaps indicate H3K4me3 and H3K27me3 modification profiles (RPM values) around TSS for active, repressive and bivalent genes defined by chromatin states in HFs (NHLFs). Histone modification profiles are shown within TSS ± 2 Kb.

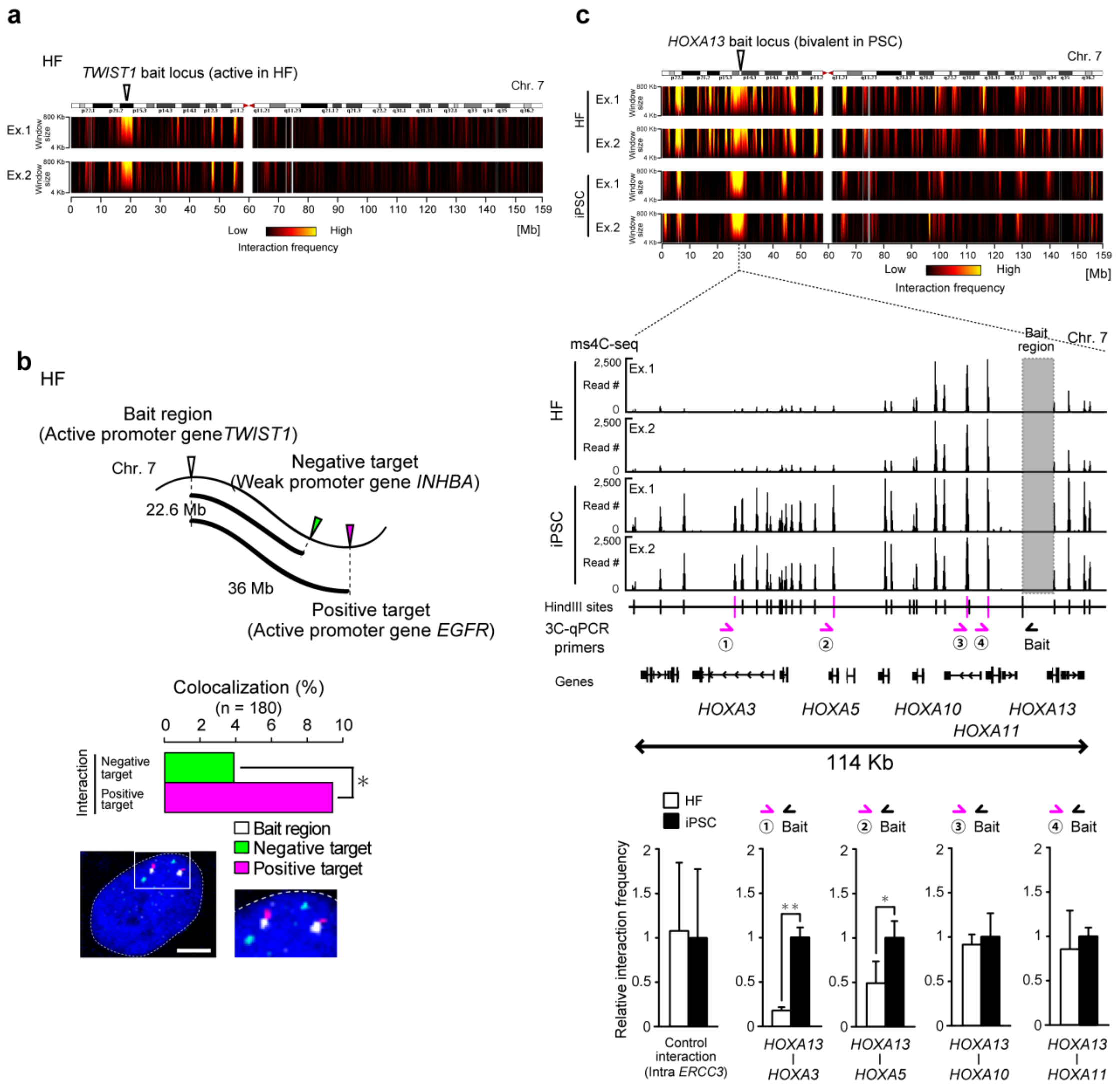

Supplementary Figure 2. Analysis of ms3-seq data

(a) Profile of the cis-chromosomal interaction in the *TWIST1* gene locus in HFs (HDFs). Chromatin interaction frequencies in the *TWIST1* gene locus are shown as described in Fig. 1c.

(b) Analysis of chromatin interactions with 3D DNA FISH in HFs (HDFs). Colocalization percentage of cis-interaction target loci in *TWIST1*. The left panel illustrates the relative positions of the positive (*EGFR*) and negative (*INHBA*) interaction target loci for the bait (*TWIST1*) locus on the genome. The bar graph shows the colocalization percentage between the bait (*TWIST1*) locus and the positive (magenta) or negative (green) interaction loci (n = 180, Fisher's exact one-sided test, *p < 0.03). Nuclei were stained with Hoechst (blue). Bait locus (*TWIST1*), interaction positive locus (*EGFR*) and interaction negative locus (*INHBA*) are indicated as white,

magenta and green. Scale bar, 5 μm.

(c) Validation of the ms3C-seq data by 3C-qPCR. Top: Cis interaction frequencies of the *HOXA13* gene locus are presented with domainograms. The color scale is the same as shown in Fig. 1b. Middle: The bar plots show ms3C-seq read distributions in each HindIII site around the *HOXA* cluster locus in iPSCs and their original cells HFs (HDFs). The locations of primers that were used for 3C-qPCR are represented by interaction target loci (magenta arrows) and bait (black arrow). Bottom: Relative interaction frequencies between *HOXA13* and each *HOXA* locus were determined by 3C-qPCR in HFs (HDFs) and iPSCs (n = 3). Interaction frequency of the *ERCC3* region is a positive control. (Means ± SD, t-test, $*p < 0.05$, $**p < 0.001$).

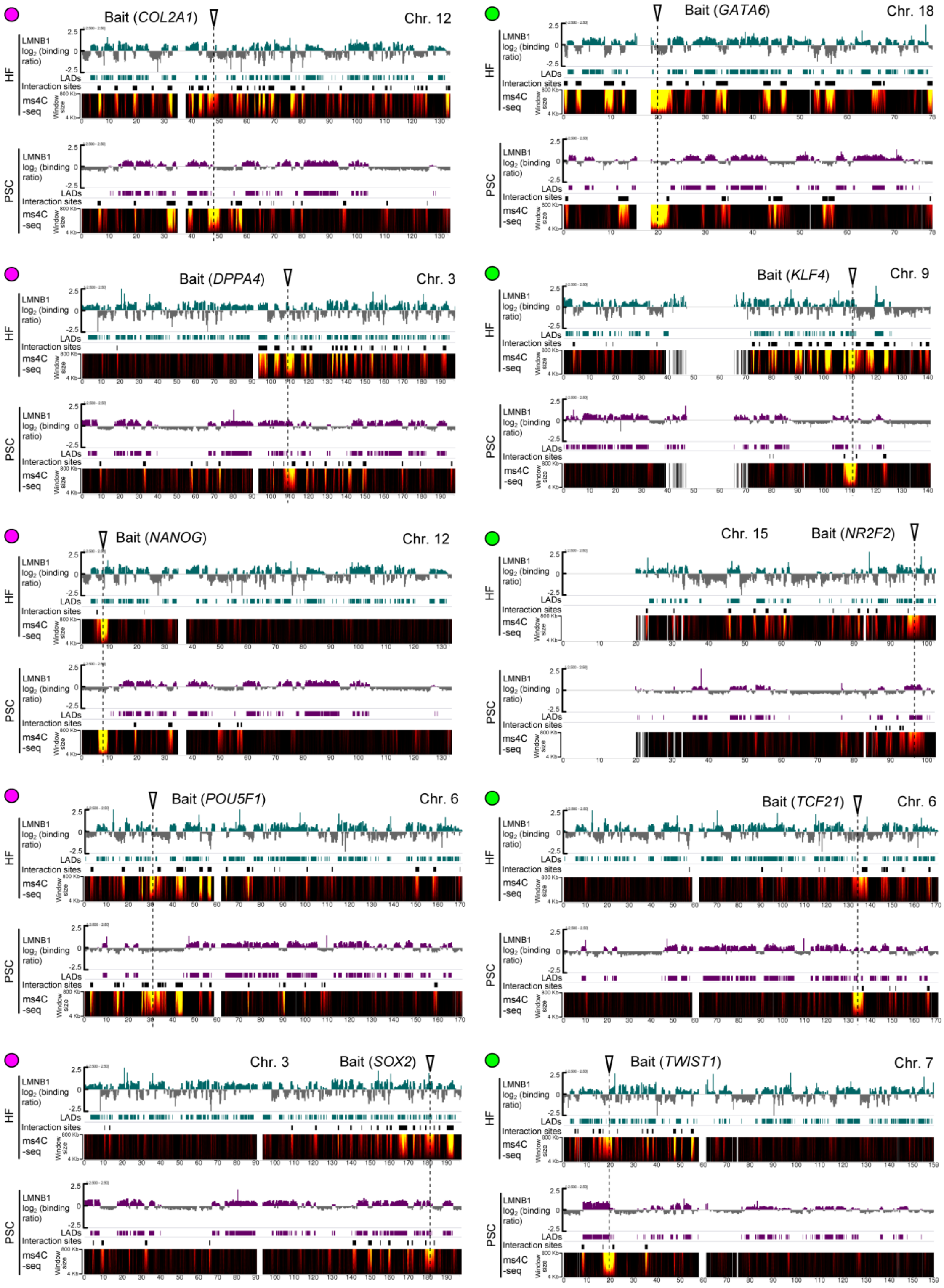
Bait (COL2A1)
Chr. 12
Bait (GATA6)
Chr. 18
Bait (DPPA4)
Chr. 3
Bait (KLF4)
Chr. 9
Bait (NANOG)
Chr. 12
Chr. 15
Bait (NR2F2)
Bait (POU5F1)
Chr. 6
Bait (TCF21)
Chr. 6
Chr. 3
Bait (SOX2)
Bait (TWIST1)
Chr. 7
HF
PSC
LMNB1
log2 (binding ratio)
2.5
0
-2.5
LADs
Interaction sites
ms4C
-seq
Window size
800 Kb
4 Kb

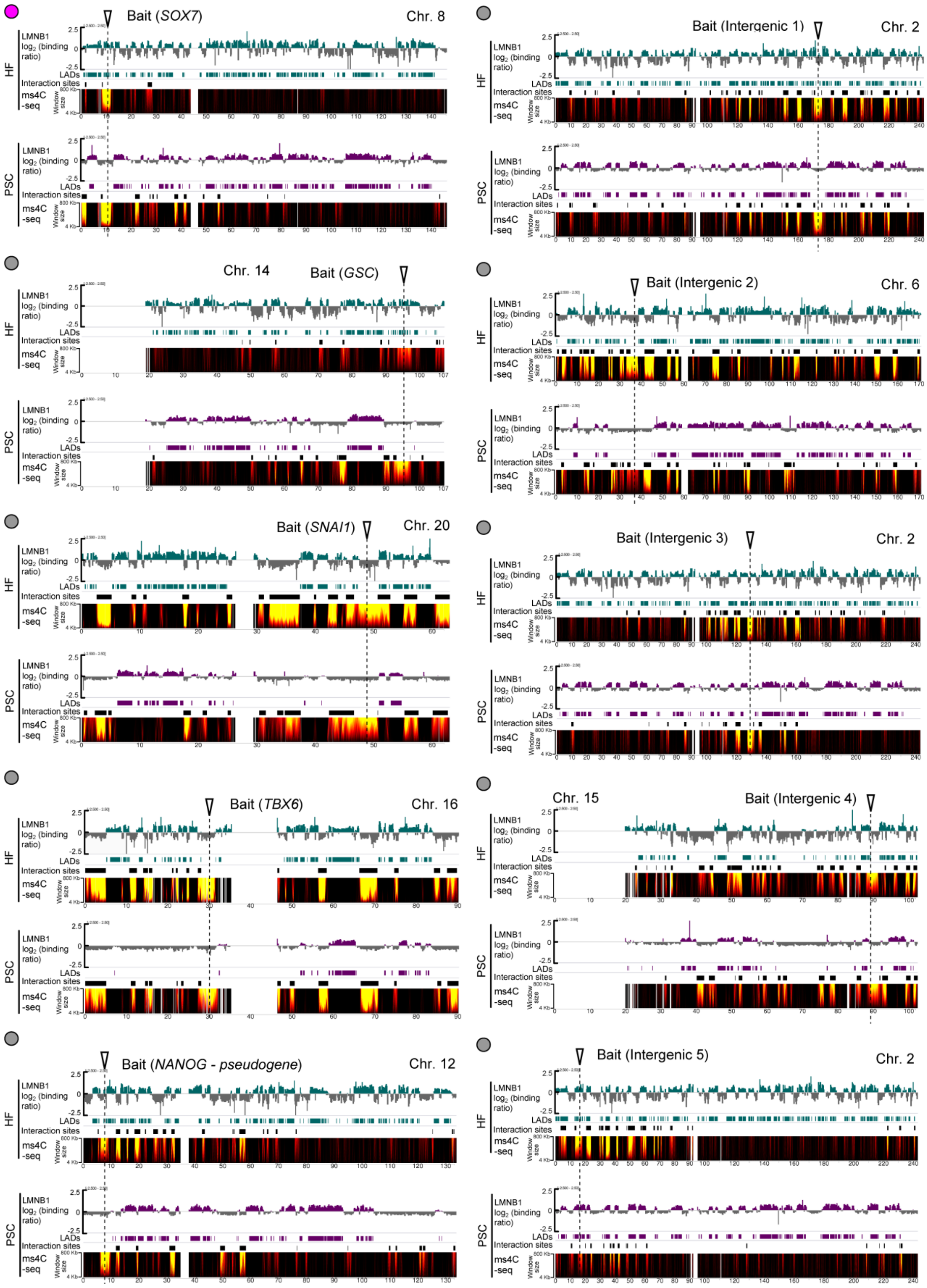

Bait (SOX7)
Chr. 8
Bait (Intergenic 1)
Chr. 2
Chr. 14
Bait (GSC)
Bait (Intergenic 2)
Chr. 6
Bait (SNAI1)
Chr. 20
Bait (Intergenic 3)
Chr. 2
Bait (TBX6)
Chr. 16
Chr. 15
Bait (Intergenic 4)
Bait (NANOG - pseudogene)
Chr. 12
Bait (Intergenic 5)
Chr. 2
HF
PSC
LMNB1
log2 (binding ratio)
LADs
Interaction sites
ms4C-seq
Window size
800 Kb
4 Kb
2.5
0
-2.5

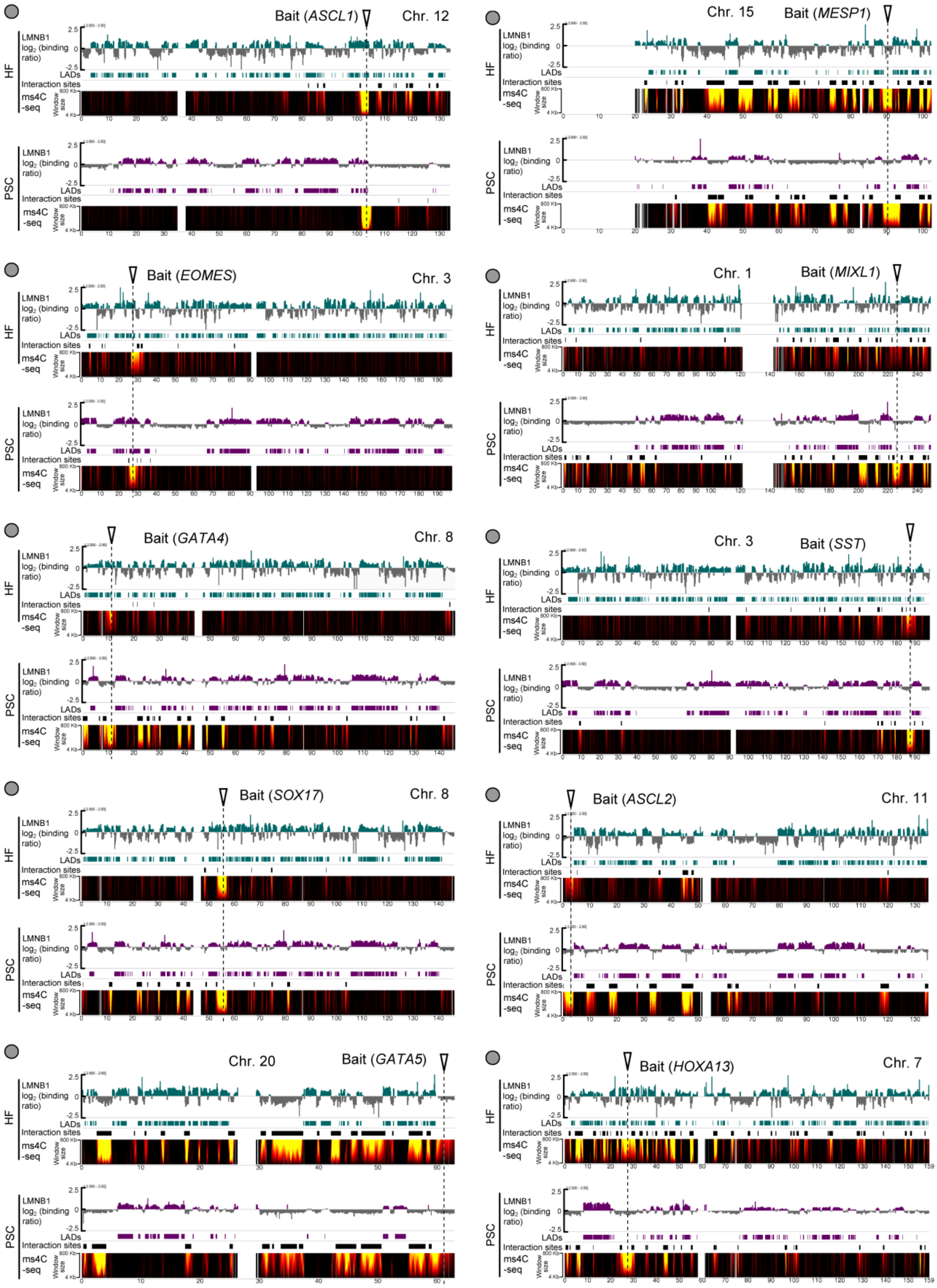
Bait (ASCL1)
Chr. 12
Chr. 15
Bait (MESP1)
Bait (EOMES)
Chr. 3
Chr. 1
Bait (MIXL1)
Bait (GATA4)
Chr. 8
Chr. 3
Bait (SST)
Bait (SOX17)
Chr. 8
Bait (ASCL2)
Chr. 11
Chr. 20
Bait (GATA5)
Bait (HOXA13)
Chr. 7
HF
PSC
LMNB1
log2 (binding ratio)
LADs
Interaction sites
ms4C-seq
Window size

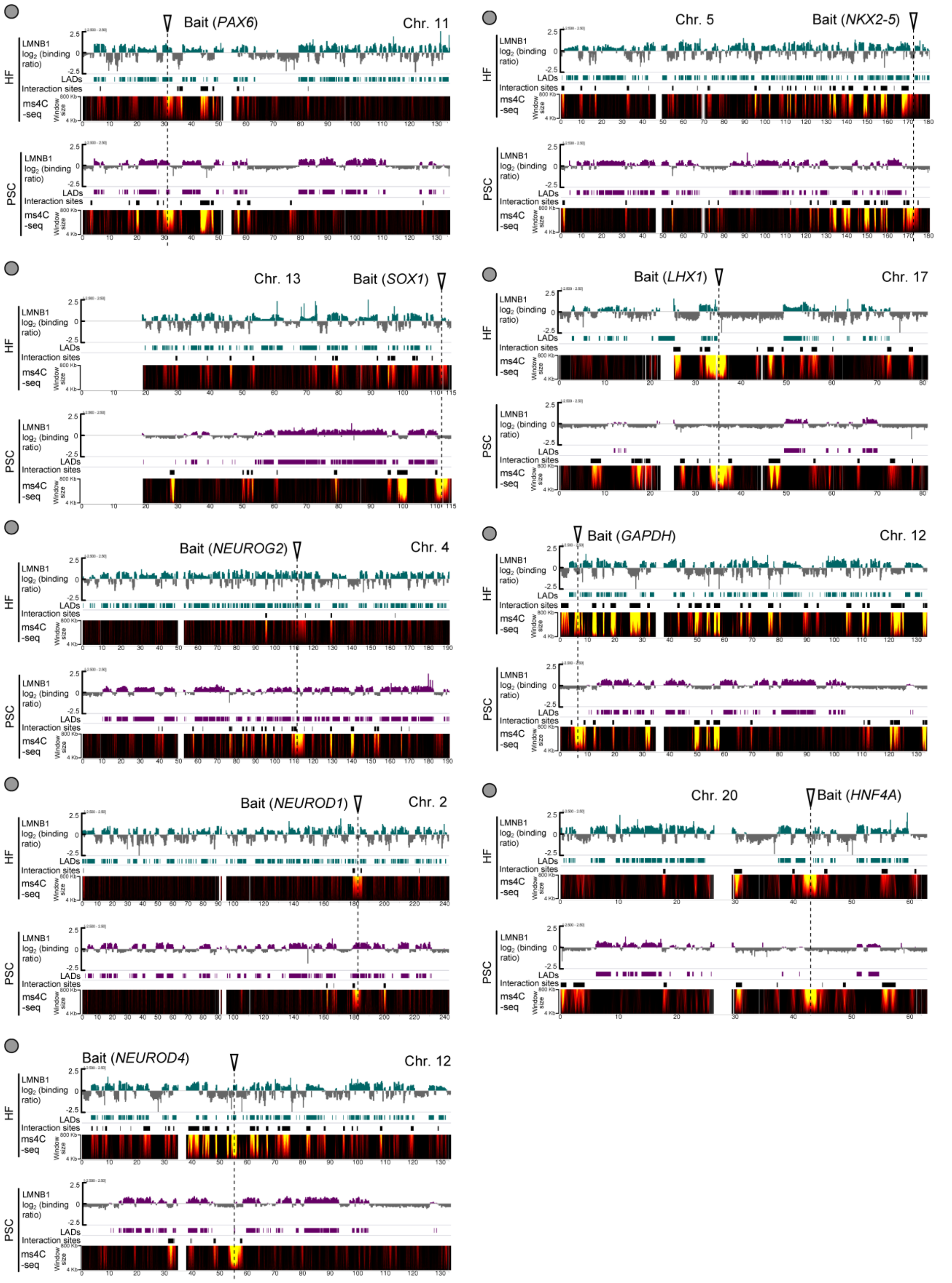

Supplementary Figure 3. LADs and chromatin interaction profiles in HFs and hPSCs

Relationship between chromatin interaction profiles and lamina association profiles at bivalent gene loci. The profiles are shown as described in Fig. 4d.

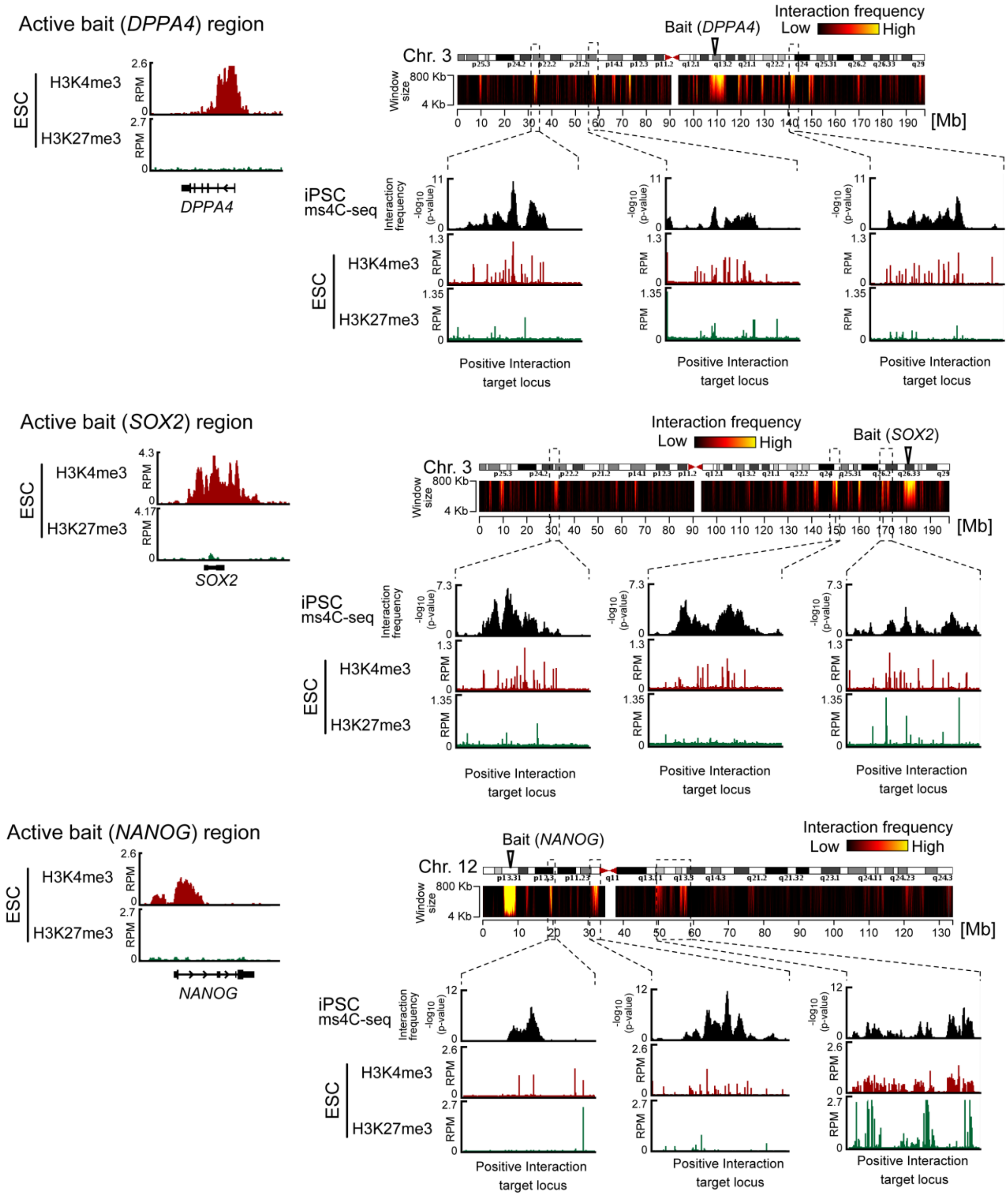

Supplementary Figure 4. Epigenetic states of the interaction target sites of the active gene locus

Histone modification patterns of H3K4me3 and H3K27me3 in the active gene loci (*NANOG*, *DPPA4* and *SOX2*) and their positive interaction target sites in hiPSCs. ChIP-seq data are represented by RPM signals for H3K4me3 (red) and H3K27me3 (green) in ESCs below ms3C-seq interaction signals in iPSCs (black).

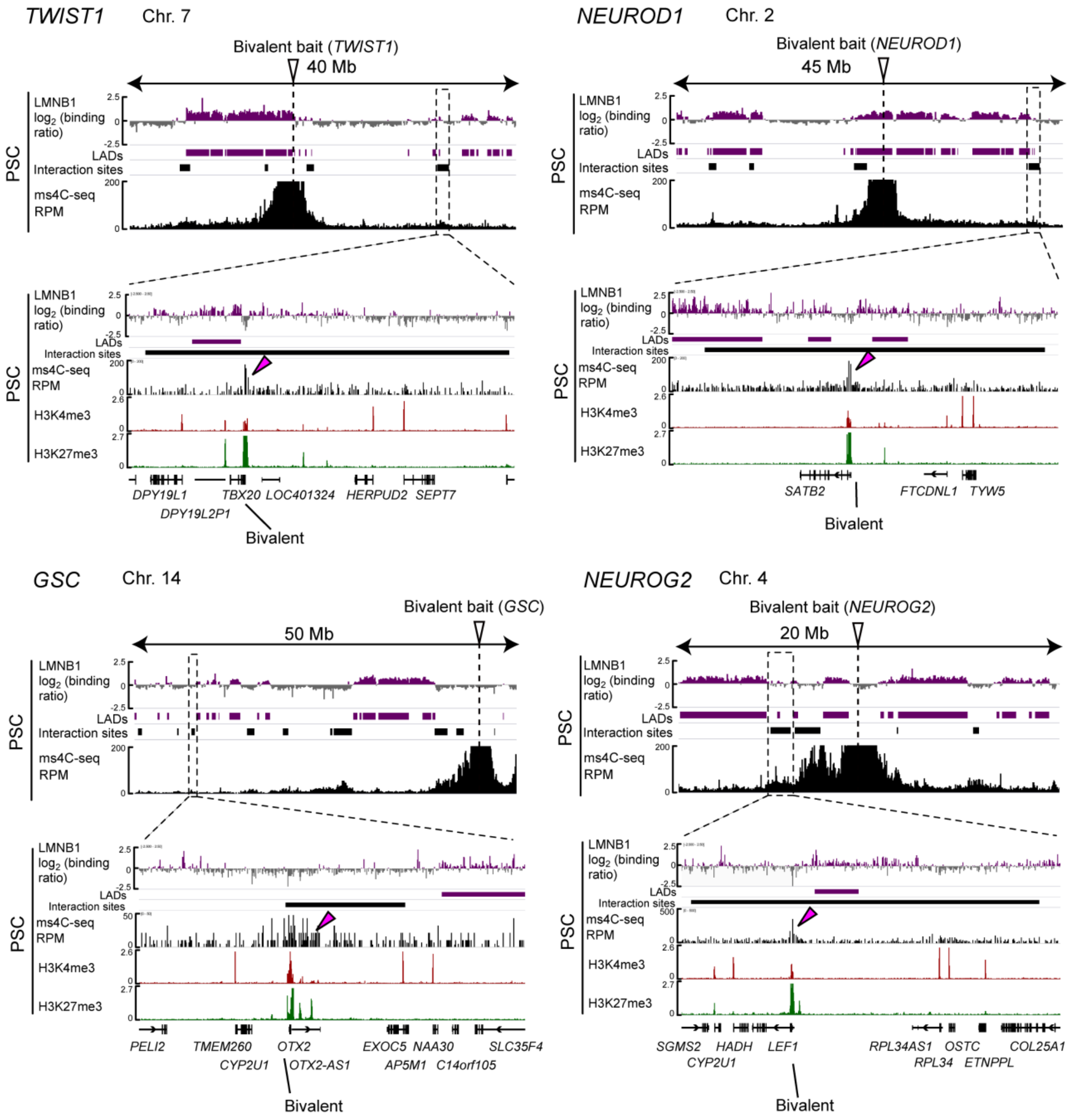

Supplementary Figure 5. Epigenetic states of the interaction target sites of the bivalent bait locus

LADs and histone modifications at interaction target sites of bivalent gene loci (*TWIST1*, *NEUROD1*, *GSC* and *NEUROG2*) in hPSCs. Lamina association signals in PSCs (ESCs) are shown as described in Fig. 2f. ChIP-seq data are represented by RPM signals for H3K4me3 (red) and H3K27me3 (green) in PSCs (ESCs) below ms3C-seq interaction signals in PSCs (iPSCs) (black).

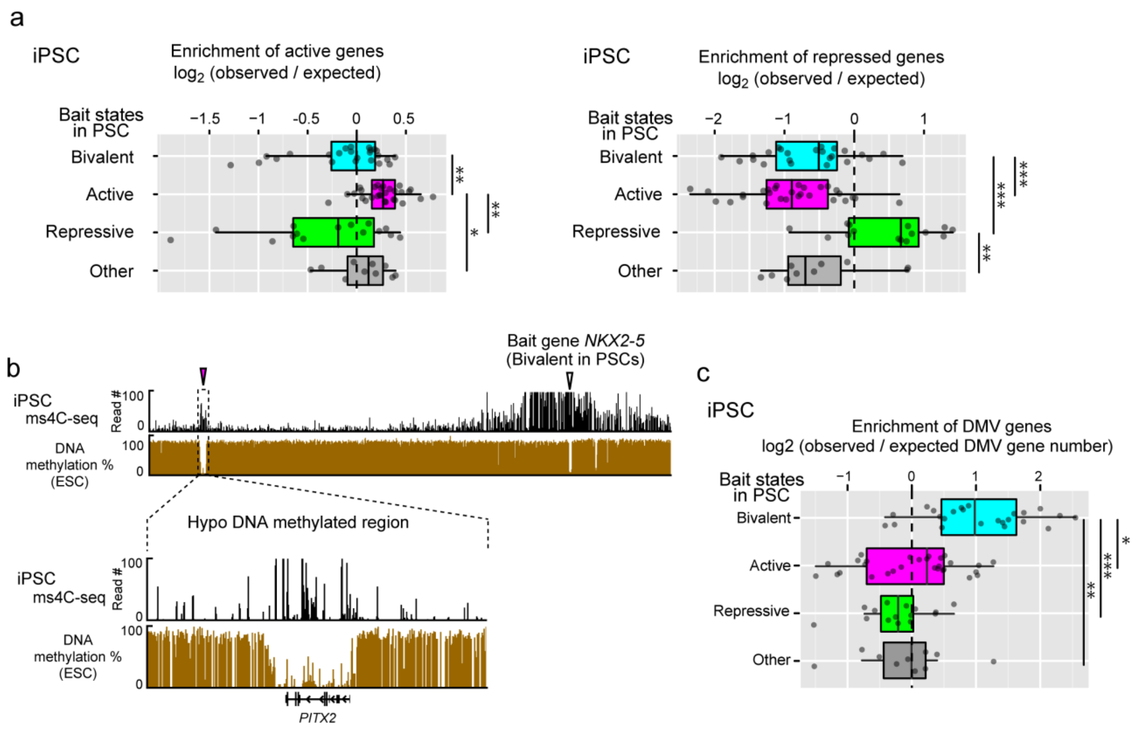

Supplementary Figure 6. Analysis of gene states in the interaction target genes of the bivalent bait locus

(a) The enrichment of active and repressive genes in the interaction target genes of bivalent bait loci in iPSCs (428C2). Box plots show log_2(observed frequency for active (left panel) or repressive genes (right panel))/(expected frequency for active (left panel) or repressive genes (right panel)) in the interaction targets of bivalent, active, repressive and other bait gene loci (U-test, * $p < 5 \times 10^{-2}$, ** $p < 4 \times 10^{-3}$, *** $p < 3 \times 10^{-5}$).

(b) Enrichment of ms3C-seq interaction signals in DNA methylation valleys in iPSCs. DNA methylation percentages at the interaction target sites of the bivalent gene (*NKX2-5*) locus are shown under the ms3C-seq signals.

(c) Enrichment of DMV genes in the interaction sites of bivalent genes in iPSCs (428C2). Box plots show log_2(observed frequency for DMV genes)/(expected frequency for DMV genes) in interaction targets of bivalent, active, repressive and other bait gene loci in PSCs (U-test, *$p < 1 \times 10^{-4}$, **$p < 1 \times 10^{-5}$,***$p < 1 \times 10^{-7}$).

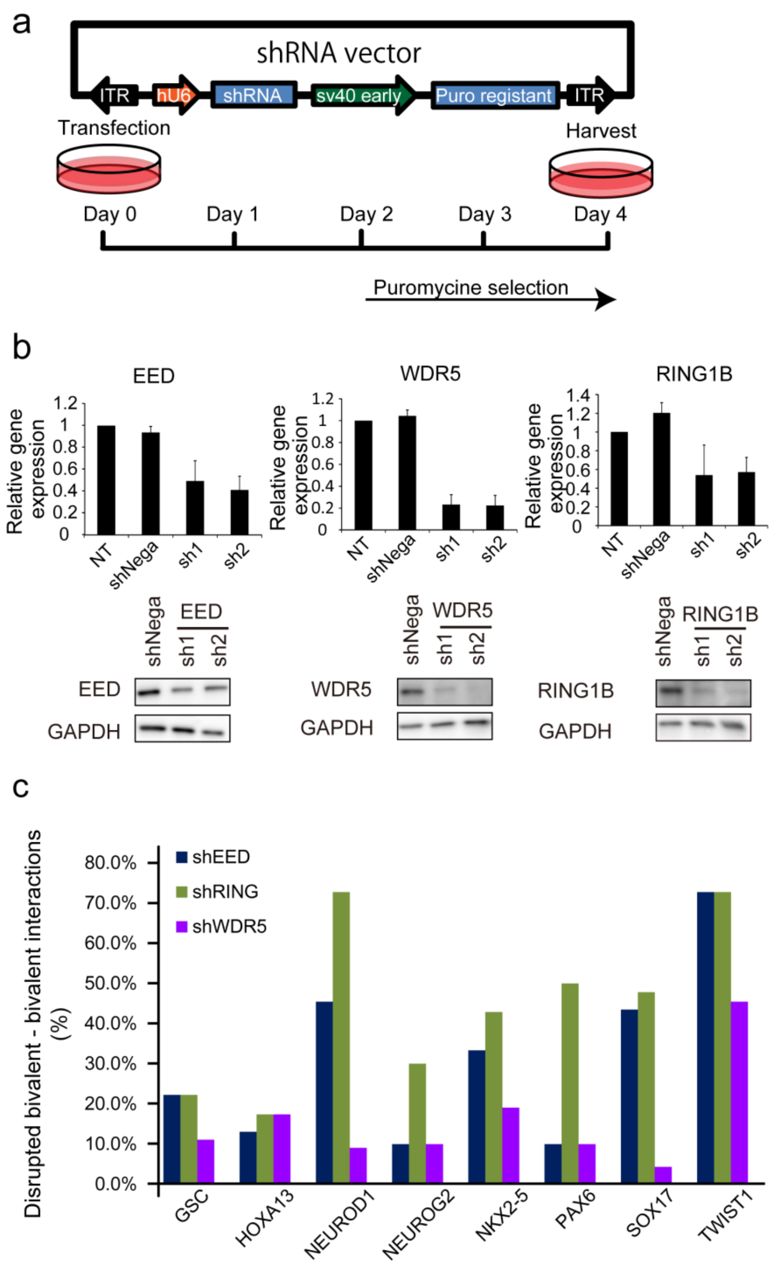

Supplementary Figure 7. shRNA ms3C-seq analysis

(a) Schematic diagram of knockdown experiments by shRNA.

(b) Analysis of the knockdown efficiencies of each shRNA in hiPSCs by qPCR (upper panels) and immunoblotting (lower panels). Error bars indicate standard deviation (n = 3).

(c) Percentages of bivalent genes with interaction signals that were reduced more than 30% in both independent shRNA knockdown experiments (sh1 and sh2) for EED, WDR5 and RING1B compared to shNega treatment in each bivalent bait.

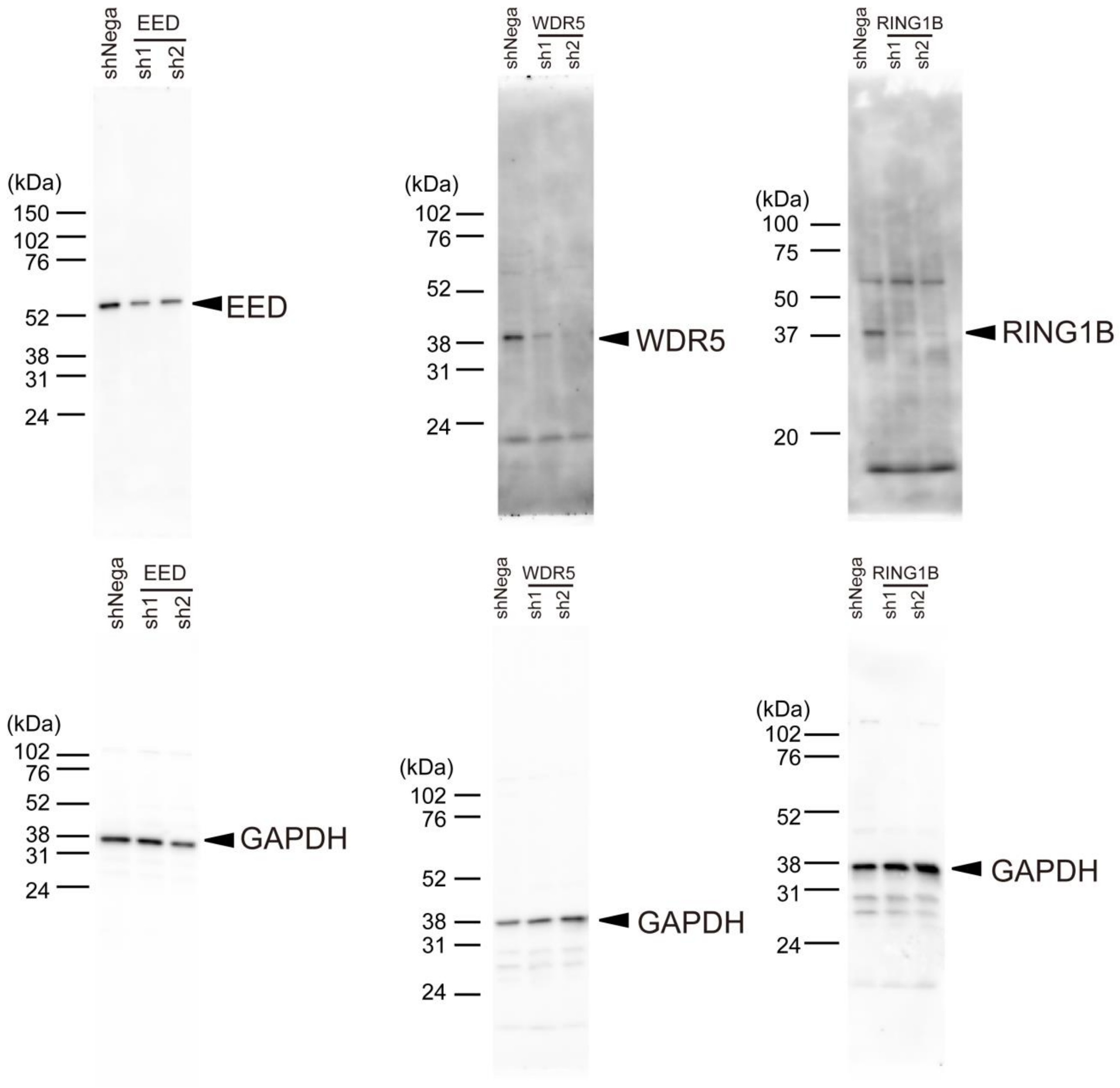

Supplementary Figure 8. Full-length images of immunoblots in Supplementary Figure 7.

Supplementary Table 1. Primers and target sequences for shRNA

List of bait and adapter primers for ms4C-seq

Bait	1st PCR primer sequence (5' to 3')	2nd PCR primer sequence (5' to 3')
ASCL1	TCATTTCTGCCACTTAGCATGT	ACACTCTTTCCCTACACGACGCTCTTCCGATCTATCCACCTCTTCCCTTTGAC
ASCL2	CTCGCTAGCTTTGGGTTTTG	ACACTCTTTCCCTACACGACGCTCTTCCGATCTCGGCTCTTGAAAGGAACAAG
COL2A1	AGCGGGATTCTCTCATCCTG	ACACTCTTTCCCTACACGACGCTCTTCCGATCTCCCCAGCCACTTTCTAGTG
DPPA4	TCGGGTCTTAGAAGCCAGAG	ACACTCTTTCCCTACACGACGCTCTTCCGATCTTTGTGCAAATCCTGCACTTC
EOMES	CTTCAACGCACAGAACCACT	ACACTCTTTCCCTACACGACGCTCTTCCGATCTCCAGTGGGAAAGGTTTGTAG
ERCC3	CCTACCCCATGTAGTGAATC	ACACTCTTTCCCTACACGACGCTCTTCCGATCTCAGTTTGTTTCCTCAAATAATGG
ETS2	ATCATTTCCTCATGTGTAAGC	ACACTCTTTCCCTACACGACGCTCTTCCGATCTCTGGCAGTGGTGGCTATTTC
EVX1	AAATGGAATTCCGCAGAAAG	ACACTCTTTCCCTACACGACGCTCTTCCGATCTGCAGCGGGACTCGGTAAT
FOXA2	ATGCATAGGAACTAGTGGGC	ACACTCTTTCCCTACACGACGCTCTTCCGATCTGCAGAAGACAGGCGAGATG
FOXF1	ACGTGTAAACACACCCATGC	ACACTCTTTCCCTACACGACGCTCTTCCGATCTATGTGCGCACCCACTAATG
GAPDH	CCTGCCCTTTGAGTTTGATG	ACACTCTTTCCCTACACGACGCTCTTCCGATCTCATAGTGGGGTGGTGAATAC
GATA4	CAAAGGGAAAAGTTGGTGACC	ACACTCTTTCCCTACACGACGCTCTTCCGATCTGGAATGAACTATTTGCCAAACC
GATA5	GATGGCGGACAGCTCTTG	ACACTCTTTCCCTACACGACGCTCTTCCGATCTCCCCACTTTCTCTAGGCACA
GATA6	CCAATGACCGACGAAAGATT	ACACTCTTTCCCTACACGACGCTCTTCCGATCTAAGGTGTCTTTCCGTGATCG
GSC	AACGAAGCCCTTCGCTAACT	ACACTCTTTCCCTACACGACGCTCTTCCGATCTAAACGTCAAGGGGATTTGAA
HNF4A	CCTCCTTCCTTTTCTTTGGG	ACACTCTTTCCCTACACGACGCTCTTCCGATCTGAGCATGTGTTCTTGGCTGT
HOXA13	AGGAAGTGTTGGGGACCTTCGG	ACACTCTTTCCCTACACGACGCTCTTCCGATCTCCTGGTAGTGATAGAGGTGATCTC
KLF4	ACCCGAATTGCTTTGAAATG	ACACTCTTTCCCTACACGACGCTCTTCCGATCTAAGGAGCTTGCAGCTTTCAC
LHX1	TCCTACCCTCCCCTCTTTCC	ACACTCTTTCCCTACACGACGCTCTTCCGATCTGATGCCGGCAGTTTAGGAT

MESP1	AGGGACCCTCATTCCCTCTA	ACACTCTTTCCCTACACGACGCTCTTCCGATCTGTAGACCCTTCCCTGGTAAG
MIXL1	CCGGCTGGAAACTGGTAAC	ACACTCTTTCCCTACACGACGCTCTTCCGATCTTCATGAGGATTCATTCTACGACTC
NANOG	TTTGTTAAGTGGGGGCTGTC	ACACTCTTTCCCTACACGACGCTCTTCCGATCTCTCACCGAGGCTGAGCTTG
NANOG-P	TTTGTTAAGTGGGGGCTGTC	ACACTCTTTCCCTACACGACGCTCTTCCGATCTCTCACCGAGGCTGAGCTTG
NEUROD1	AACGAACCCACTGTGCTTAC	ACACTCTTTCCCTACACGACGCTCTTCCGATCTTGGGTACTACTGCTGCAAAGTG
NEUROD4	GGGCAGTTAGTTCCTTGAGTTAC	ACACTCTTTCCCTACACGACGCTCTTCCGATCTTTAAGCATTCACGTAGAAAAAGTCC
NEUROG2	GGAGCTGAGTGCGTGTAGTG	ACACTCTTTCCCTACACGACGCTCTTCCGATCTGAGAGGGTGGGGATCTTTAG
NKX2-5	AGCTACATGGCCAGATCCTG	ACACTCTTTCCCTACACGACGCTCTTCCGATCTCACTCCTTGGGAGGTCTGAT
NR2F2	AAAGGGGTTCCCGATTAAGA	ACACTCTTTCCCTACACGACGCTCTTCCGATCTTAGCAGGGCTTGGAACCTAG
PAX3	TGAAAAGGGGGCTCAGAGAG	ACACTCTTTCCCTACACGACGCTCTTCCGATCTGGCTCGGGAGCATTTATTAG
PAX6	GGCCTGGCTATCTTGGAATGG	ACACTCTTTCCCTACACGACGCTCTTCCGATCTCGTCCCTCATCCCTCAAAAC
POU5F1	TCCAGGTGGTGGAGGTGATG	ACACTCTTTCCCTACACGACGCTCTTCCGATCTTGGGTTGATCCTCGGACCTG
Intergenic 1	AACCAGGAAGGAGGGAGAGA	ACACTCTTTCCCTACACGACGCTCTTCCGATCTAAGAATTTCGGAGAGCCACC
Intergenic 2	GAGAGATGGAGGCATTTTGC	ACACTCTTTCCCTACACGACGCTCTTCCGATCTCAAGTCAGTAGGGGGCTAAG
Intergenic 3	TTACAGGCAGGACAAGAGCC	ACACTCTTTCCCTACACGACGCTCTTCCGATCTACTCAGTGACTGCGCTGG
Intergenic 4	TAGTCAGTGGGGAGGGACAC	ACACTCTTTCCCTACACGACGCTCTTCCGATCTGCTTTTCAAGGTAATGTCAGCC
Intergenic 5	ACCTCCCAGCCAAGAACC	ACACTCTTTCCCTACACGACGCTCTTCCGATCTAGGCGATAACGAGGTGAGAG
SNAI1	TTGGTGTCATAGAGGAGTGGG	ACACTCTTTCCCTACACGACGCTCTTCCGATCTTCTTTGGTAAGGGAGGAGGG
SOX1	TCAGTCCTCCAAGATGCTGG	ACACTCTTTCCCTACACGACGCTCTTCCGATCTGAGACACGTTGGAATTGGAC
SOX17	CCTTGGCTAGGGGACAAGTT	ACACTCTTTCCCTACACGACGCTCTTCCGATCTTGGTAGTTTTGAAAGCGGTTG
SOX2	AGACGCCACAAAGGGCAGAC	ACACTCTTTCCCTACACGACGCTCTTCCGATCTTGATTGTGTATGCATCGGTCTGC
SOX7	CATCTCAGAGGAAGGCTGTC	ACACTCTTTCCCTACACGACGCTCTTCCGATCTAGACCCTGAGGAACGAATCC

SST	TCCTGAGACCTTCAGTGCCT	ACACTCTTTCCCTACACGACGCTCTTCCGATCTGAAGTGGCATGGTCACAGG
T	TTGTTCCCGCTCTGGTTAAC	ACACTCTTTCCCTACACGACGCTCTTCCGATCTCATTTCTACAAGCCCCTCTTC
TBX6	AGAAAAAGAGAAGCCCCTCG	ACACTCTTTCCCTACACGACGCTCTTCCGATCTAGACAGTAGCAGGGTCCCAC
TCF21	AGCTGCCACATGAAATGAAC	ACACTCTTTCCCTACACGACGCTCTTCCGATCTAGAAAAACTGGCTTGAGTGATG
TWIST1	AAGGATGGCTCCCTTTTTAC	ACACTCTTTCCCTACACGACGCTCTTCCGATCTAATTCTGGATTCTCTTATATATGCAAC
ZIC1	TAGATCCAATTGTATTAGGGATTAC	ACACTCTTTCCCTACACGACGCTCTTCCGATCTTTCCACTGCAGATGTAAGAATTT
LIN28B	GGGGCTTTCTGCAAACCTAT	ACACTCTTTCCCTACACGACGCTCTTCCGATCTTACCGCCCACACTGATTCAC
YTHDF2	TAGCTTGAGCATGTGGGAGC	ACACTCTTTCCCTACACGACGCTCTTCCGATCTCTGTGTCTTGGCATAACCTGTG
YBX1	AGTGCCATCTCAGCATTGTC	ACACTCTTTCCCTACACGACGCTCTTCCGATCTGTGGAATGGACAACAAATGGGC
ZZZ3	AGCCAAGTGGAAAGGGATGG	ACACTCTTTCCCTACACGACGCTCTTCCGATCTGGAGCCCCTGAGATCCTCAA
MYCN	AGAAGGTACTGCAGAGTCAAGG	ACACTCTTTCCCTACACGACGCTCTTCCGATCTCGGGGAGAGGACTGGAGTTC
MITD1	CGTTGCGTTAAAGTGGAAGTGC	ACACTCTTTCCCTACACGACGCTCTTCCGATCTCCACAGTGAAACCAGATCCCA
DPH3	ATAGGCAATGACGCAACTCC	ACACTCTTTCCCTACACGACGCTCTTCCGATCTGGTAGCGGTCACCATGGAG
ZFP42	GGTCGTATAAGTACACCCAGACC	ACACTCTTTCCCTACACGACGCTCTTCCGATCTCCAGACCATTAGAACGAAACCC
KIF2A	TGGTGGAAGGCAGGAGATTAG	ACACTCTTTCCCTACACGACGCTCTTCCGATCTTGCATTGGACAGTAGTGTTTCAC
PTPRZ1	AGCATTACAAGAAGTATGCAGGTG	ACACTCTTTCCCTACACGACGCTCTTCCGATCTACTGACTGTGCACTGAGAGTTC
PODXL	AAAAACTGAAGCCCCAGTGC	ACACTCTTTCCCTACACGACGCTCTTCCGATCTAAGATTTGGAAGGGGTGGTCTG
TAF2	AAGTCACGTCTCGCAGCTG	ACACTCTTTCCCTACACGACGCTCTTCCGATCTCAACCTCGCTCCTTCGACTA
FGFR2	GCCCTCCCGCTAAAGAGAC	ACACTCTTTCCCTACACGACGCTCTTCCGATCTCTGCTTGATACTGCGGGGAG
STX5	TCCAAGGATGCCCAAAGTTC	ACACTCTTTCCCTACACGACGCTCTTCCGATCTGGTTCTTGAGGCTCTTGGGG
BCAT1	GAAGAGTTGACCCGGGTGG	ACACTCTTTCCCTACACGACGCTCTTCCGATCTGTTAAAAGCAGAAGGCCCCT
METAP2	TGGATTGTGATTGGCTGCCT	ACACTCTTTCCCTACACGACGCTCTTCCGATCTTGTCCACCTGGAGAGTTTAGG
KDM2B	ATACGACGAGAACGAGGACTTG	ACACTCTTTCCCTACACGACGCTCTTCCGATCTTGTCGGACGTGGAGGAGATC

NCL	TACCAGGTACCCCCACGTG	ACACTCTTTCCCTACACGACGCTCTTCCGATCTGGTTGTCTCGCTGGGAAAGG
LIG1	TCTGTGATATTCTGCCCTTTGT	ACACTCTTTCCCTACACGACGCTCTTCCGATCTGCTAATTTATGTAGACCAGCGGG
DNMT3B	AAGCCCCTGTTCATGCTCTC	ACACTCTTTCCCTACACGACGCTCTTCCGATCTAGTCTGGACCCCGGGAAG
SALL4	CATTTTGGGTGGGCGCAC	ACACTCTTTCCCTACACGACGCTCTTCCGATCTCTTTTGGTGGTGTTTGGCAC
ACO2	AGAGTTTCATGCACCTGCCA	ACACTCTTTCCCTACACGACGCTCTTCCGATCTGAGAGCAAGACGGGCCTG
ARHGEF38	AGCGGTTTAGTTAGAAGGGAGC	ACACTCTTTCCCTACACGACGCTCTTCCGATCTAATCAGCCATTGCCTGCAAG
SLC34A1	TTCTCAGGTTCAAGCTCTGGG	ACACTCTTTCCCTACACGACGCTCTTCCGATCTAGTTTCTGGGTTCGGGTGTC
GIMAP1	GCCCCTGAGGAGACATCTGA	ACACTCTTTCCCTACACGACGCTCTTCCGATCTTCTTCAACCCCTTTCCCTGC
PRSS3	GCACACTATTAAAAGAACACAAGCG	ACACTCTTTCCCTACACGACGCTCTTCCGATCTCCTCCCTCTCAGACCTACTG
HEMGN	TCCTGTTGGGAGTGGGAAAC	ACACTCTTTCCCTACACGACGCTCTTCCGATCTGAAGCCTCCAAGATTGTTCGC
TNFSF8	AGGCGAAAGAGAAGACCATGG	ACACTCTTTCCCTACACGACGCTCTTCCGATCTTGGCTTCTTAGAGCTTTGGACA
OBP2B	TCATGTACCTGCAGGAGCTG	ACACTCTTTCCCTACACGACGCTCTTCCGATCTTTACTGCAAAGACCAGCACC
ANKRD1	AGGCGAAACTCATCAAAAGTACC	ACACTCTTTCCCTACACGACGCTCTTCCGATCTTTCCTAAAAGAAATGTGATTAAGCAG
SOX6	GCCAGTTAAATCAAATCATGGCAC	ACACTCTTTCCCTACACGACGCTCTTCCGATCTTAGGGCAATGTTGGATAAAGTC
FLRT1	CGTTCCCACAGGCTCCAG	ACACTCTTTCCCTACACGACGCTCTTCCGATCTAGGTCTGGTCTCTGTGCTCA
KRTAP5-11	GTTCCCCATGCCCTGGTG	ACACTCTTTCCCTACACGACGCTCTTCCGATCTAGCATTTCTCCAGCCCCTTC
GAST	GGGACAGCCTCACCCTTAAG	ACACTCTTTCCCTACACGACGCTCTTCCGATCTGCTGATCTTTGCACTGGCTC
DEFB132	TCCTCCCTGACCGTGAAGAC	ACACTCTTTCCCTACACGACGCTCTTCCGATCTTCCCCACCCCTGTTTTTACC
MC3R	TGCAATTGTGTGTTTATCTTGCAC	ACACTCTTTCCCTACACGACGCTCTTCCGATCTAACCATTCCTTTCTTGCTTTGG
VPREB1	TATGACCTGTTGCTCCTCCC	ACACTCTTTCCCTACACGACGCTCTTCCGATCTGGCAGTGGGGAACTAACCAG
PRAME	ACAGGGAGATTGCACAGCTC	ACACTCTTTCCCTACACGACGCTCTTCCGATCTAAGCGGGGATCCCATGGAAG
IGLL5	TCAGGCAAGGAAGAGTGGTG	ACACTCTTTCCCTACACGACGCTCTTCCGATCTATCTAGACCTAGGTTACCTCTGGG

COLQ	GAGGGTGGGCAGGAAAGG	ACACTCTTTCCCTACACGACGCTCTTCCGATCTCCACATGCCCCTGCAAATTC
CBLN2	GAACCGGACAGTTCCCCAAC	ACACTCTTTCCCTACACGACGCTCTTCCGATCTGACTCTGGAACCACAGCTCC
TREM1	GAAGAGGACTGAGCAGGGGA	ACACTCTTTCCCTACACGACGCTCTTCCGATCTGTTTGGTCCAGGATCTCGGG

Adaptor primers for ms4C-seq

Name	Primer sequence (5' to 3')
1st PCR adaptor primer	CGAAGAGTAACCGTTGCTAGG
2nd PCR adaptor primer (P5)	AATGATACGGCGACCACCGAGATCTACACTCTTTCCCTACACGAC
2nd PCR adaptor primer (P7) (NNNNNN = Index)	CAAGCAGAAGACGGCATACGAGATNNNNNNGTGACTGGAGTTCAGACGTGTGC

Target genes of shRNA and target sequence for EED, RING1B and WDR5

Target gene		Target sequence (5' to 3')
EED sh1	TOP	CCGGTGCACAGAGATGAAGTTCTAAGTGCCTCGAGGCACTTAGAACTTCATCTCTGTGCTTTTTTC
	BOTTOM	AATTGAAAAAAGCACAGAGATGAAGTTCTAAGTGCCTCGAGGCACTTAGAACTTCATCTCTGTGCA
EED sh2	TOP	CCGGTGGATCATTCTCTTAAACTTTGGAGCTCGAGCTCCAAAGTTTAAGAGAATGATCCTTTTTTC
	BOTTOM	AATTGAAAAAAGGATCATTCTCTTAAACTTTGGAGCTCGAGCTCCAAAGTTTAAGAGAATGATCCA
RING1B sh1	TOP	CCGGTGGCTAGAGCTTGATAATAACAATGCTCGAGCATTGTTATTATCAAGCTCTAGCCTTTTTTC
	BOTTOM	AATTGAAAAAAGGCTAGAGCTTGATAATAACAATGCTCGAGCATTGTTATTATCAAGCTCTAGCCA
RING1B sh2	TOP	CCGGTGCACAGACGAGATACATAAAGACTCTCGAGAGTCTTTATGTATCTCGTCTGTGCTTTTTTC
	BOTTOM	AATTGAAAAAAGCACAGACGAGATACATAAAGACTCTCGAGAGTCTTTATGTATCTCGTCTGTGCA
WDR5 sh1	TOP	CCGGTGGGTGAAGTTCAATTTAACATGCGCTCGAGCGCATGTTAAATTGAACTTCACCCTTTTTTC

	BOTTOM	AATTGAAAAAAGGGTGAAGTTCAATTTAACATGCGCTCGAGCGCATGTTAAATTGAACTTCACCCA
WDR5 sh2	TOP	CCGGTGGGAAGAGTTCCTAGTCTATTGTGCTCGAGCACAATAGACTAGGAACTCTTCCCTTTTTTC
	BOTTOM	AATTGAAAAAAGGGAAGAGTTCCTAGTCTATTGTGCTCGAGCACAATAGACTAGGAACTCTTCCCA
shNega	TOP	CCGGTCCTAAGGTTAAGTCGCCCTCGCTCGAGCGAGGGCGACTTAACCTTAGGTTTTTTC
	BOTTOM	AATTGAAAAAACCTAAGGTTAAGTCGCCCTCGCTCGAGCGAGGGCGACTTAACCTTAGGA

List of primers for 3C qPCR and enhanced 3C qPCR

Detection target of interaction	Primer type (target gene name)	Sequences (5' to 3')
HOXA13 - HOXA (X)	Bait (HOXA13)	CCTCCACCTGGTAGTGATAG
	Interaction Target (HOXA3)	GTCAAAAATGCGCCAGAACT
	Interaction Target (HOXA5)	GGTTTAGCCACCAACTCCTG
	Interaction Target (HOXA10)	AGCTGCACAAAGGTGCTGTA
	Interaction Target (HOXA11)	CCTCACTCTCAGGCTCTTGG
PAX6 - WT1	Bait (PAX6)	GGCCTGGCTATCTTGGAATGG
	Interaction Target (WT1)	AGCAAGAGCCAGACTCAAGG
NKX2-5 - MSX2	Bait (NKX2-5)	CACTCCTTGGGAGGTCTGAT
	Interaction Target (MSX2)	TTTCATGGGAAGGTCACAGC
NEUROG2 - PITX2	Bait (NEUROG2)	AAAGAAAGGGAGCTGAGTGC
	Interaction Target (PITX2)	GGCAGACAGAAAAAGCAAGC
TWIST1 - SP8	Bait (TWIST1)	TCCACATTTGCAAGGATGGC
	Interaction Target (SP8)	TGCAACGTAGTGGGTTCCAG
GATA5 - HRH3	Bait (GATA5)	CCCCACTTTCTCTAGGCACA

	Interaction Target (HRH3)	TCAGGGGAATCTGTGCTTTCC
Intra ERCC3	Bait (ERCC3 forward)	CCCTTTGTTCAAGCCAAAAA
	Interaction Target (ERCC3 reverse)	GCCCTACCCCATGTAGTGAA

List of primers for qPCR analysis

Genes	Directions	Primer sequences (5' to 3')
RING1B	Forward	CACAGCCCTTAGAAGTGGCAA
	Reverse	GTCTGGCCTTAGTGATCTTTTGG
WDR5	Forward	AATTTGGGGCGCGTATGATG
	Reverse	AATCTGACGACCAGGCTACATC
EED	Forward	GTGACGAGAACAGCAATCCAG
	Reverse	TATCAGGGCGTTCAGTGTTTG
GAPDH	Forward	ACGAATTTGGCTACAGCAAC
	Reverse	CAGTGAGGGTCTCTCTCTTC

Supplementary Table 2. Chromatin states of bait genes, observed and expected read numbers of ms4C-seq

*Total expected interaction numbers were calculated by preseq software.

Daley, T. & Smith, A. D. Predicting the molecular complexity of sequencing libraries. Nature methods 10, 325-327, doi:10.1038/nmeth.2375 (2013).

Samples	Baits	Gene states in HFs	Gene group in HFs	Gene states in PSCs	Gene group in PSCs	Total observed interactions	Interaction Variety	*Percentage of observed distinct interactions in expected interactions
HF (TIG120) Ex1	*ERCC3*	1_Active_Promoter	Active	1_Active_Promoter	Active	62,734	5,893	52.82596029
HF (TIG120) Ex1	*GAPDH*	1_Active_Promoter	Active	1_Active_Promoter	Active	993,064	10,834	92.833946
HF (TIG120) Ex1	*TBX6*	1_Active_Promoter	Active	2_Weak_Promoter	Active	206,958	5,496	83.44340697
HF (TIG120) Ex1	*FOXF1*	1_Active_Promoter	Active	3_Poised_Promoter	Bivalent	83,976	4,760	68.06806807
HF (TIG120) Ex1	*KLF4*	1_Active_Promoter	Active	3_Poised_Promoter	Bivalent	188,439	9,486	83.56604854
HF (TIG120) Ex1	*NR2F2*	1_Active_Promoter	Active	3_Poised_Promoter	Bivalent	238,908	9,660	85.57457213
HF (TIG120) Ex1	*TCF21*	1_Active_Promoter	Active	3_Poised_Promoter	Bivalent	1,102,909	21,176	94.76246733
HF (TIG120) Ex1	*TWIST1*	1_Active_Promoter	Active	3_Poised_Promoter	Bivalent	527,644	13,892	85.49396582
HF (TIG120) Ex1	*SNAI1*	2_Weak_Promoter	Active	2_Weak_Promoter	Active	1,240,991	7,719	96.35741749
HF (TIG120) Ex1	*GATA6*	2_Weak_Promoter	Active	3_Poised_Promoter	Bivalent	223,296	6,829	83.57197053
HF (TIG120) Ex1	*MESP1*	2_Weak_Promoter	Active	3_Poised_Promoter	Bivalent	1,103,097	11,255	94.48534659
HF (TIG120) Ex1	*SOX2*	3_Poised_Promoter	Bivalent	1_Active_Promoter	Active	2,523,898	23,056	97.74337277
HF (TIG120) Ex1	*MIXL1*	3_Poised_Promoter	Bivalent	2_Weak_Promoter	Active	1,170,054	23,152	95.19697699
HF (TIG120) Ex1	*COL2A1*	3_Poised_Promoter	Bivalent	3_Poised_Promoter	Bivalent	890,762	17,620	94.48278451

HF (TIG120) Ex1	*EOMES*	3_Poised_Promoter	Bivalent	3_Poised_Promoter	Bivalent	489,686	19,240	87.69011299
HF (TIG120) Ex1	*HOXA13*	3_Poised_Promoter	Bivalent	3_Poised_Promoter	Bivalent	2,297,370	15,214	97.07697118
HF (TIG120) Ex1	*NEUROD1*	3_Poised_Promoter	Bivalent	3_Poised_Promoter	Bivalent	955,650	23,533	90.81366855
HF (TIG120) Ex1	*NEUROG2*	3_Poised_Promoter	Bivalent	3_Poised_Promoter	Bivalent	1,186,819	25,100	94.55819473
HF (TIG120) Ex1	*SOX7*	3_Poised_Promoter	Bivalent	3_Poised_Promoter	Bivalent	195,390	10,031	85.66621689
HF (TIG120) Ex1	*ETS2*	4_Strong_Enhancer	Other	2_Weak_Promoter	Active	11,766	1,597	63.73722861
HF (TIG120) Ex1	*Intergenic 3*	7_Weak_Enhancer	Other	5_Strong_Enhancer	Other	286,979	18,455	77.3525356
HF (TIG120) Ex1	*POU5F1*	11_Weak_Txn	Other	2_Weak_Promoter	Active	1,219,484	13,862	90.0270172
HF (TIG120) Ex1	*NANOG_P*	11_Weak_Txn	Other	11_Weak_Txn	Other	382,846	10,383	82.23441918
HF (TIG120) Ex1	*NANOG*	12_Repressed	Repressive	2_Weak_Promoter	Active	66,600	6,977	73.63510675
HF (TIG120) Ex1	*ASCL1*	12_Repressed	Repressive	3_Poised_Promoter	Bivalent	147,879	8,722	85.40095956
HF (TIG120) Ex1	*ASCL2*	12_Repressed	Repressive	3_Poised_Promoter	Bivalent	358,112	12,775	84.73845501
HF (TIG120) Ex1	*EVX1*	12_Repressed	Repressive	3_Poised_Promoter	Bivalent	33,899	5,582	61.96301312
HF (TIG120) Ex1	*FOXA2*	12_Repressed	Repressive	3_Poised_Promoter	Bivalent	34,872	4,509	70.05033557
HF (TIG120) Ex1	*GATA4*	12_Repressed	Repressive	3_Poised_Promoter	Bivalent	422,872	13,644	88.70396255
HF (TIG120) Ex1	*GATA5*	12_Repressed	Repressive	3_Poised_Promoter	Bivalent	672,942	8,024	89.27856157
HF (TIG120) Ex1	*GSC*	12_Repressed	Repressive	3_Poised_Promoter	Bivalent	401,282	11,482	89.8449115
HF (TIG120) Ex1	*LHX1*	12_Repressed	Repressive	3_Poised_Promoter	Bivalent	96,924	6,986	76.50021901
HF (TIG120) Ex1	*PAX3*	12_Repressed	Repressive	3_Poised_Promoter	Bivalent	19,278	4,456	55.48154143
HF (TIG120) Ex1	*PAX6*	12_Repressed	Repressive	3_Poised_Promoter	Bivalent	1,073,767	15,460	90.65321919
HF (TIG120) Ex1	*SOX1*	12_Repressed	Repressive	3_Poised_Promoter	Bivalent	760,801	12,669	93.15441176
HF (TIG120) Ex1	*SOX17*	12_Repressed	Repressive	3_Poised_Promoter	Bivalent	561,666	13,412	90.76021492

HF (TIG120) Ex1	*SST*	12_Repressed	Repressive	3_Poised_Promoter	Bivalent	998,996	17,179	92.94134832
HF (TIG120) Ex1	*T*	12_Repressed	Repressive	3_Poised_Promoter	Bivalent	124,368	13,178	74.7641282
HF (TIG120) Ex1	*ZIC1*	12_Repressed	Repressive	3_Poised_Promoter	Bivalent	44,422	6,576	55.97977373
HF (TIG120) Ex1	*DPPA4*	13_Heterochrom/lo	Repressive	1_Active_Promoter	Active	685,051	19,614	88.13577601
HF (TIG120) Ex1	*Intergenic 1*	13_Heterochrom/lo	Repressive	4_Strong_Enhancer	Other	1,284,276	23,404	93.02066773
HF (TIG120) Ex1	*Intergenic 2*	13_Heterochrom/lo	Repressive	5_Strong_Enhancer	Other	1,583,584	16,133	94.11222531
HF (TIG120) Ex1	*Intergenic 4*	13_Heterochrom/lo	Repressive	5_Strong_Enhancer	Other	1,332,349	12,728	95.03117184
HF (TIG120) Ex1	*Intergenic 5*	13_Heterochrom/lo	Repressive	5_Strong_Enhancer	Other	1,098,083	21,392	89.86385156
HF (TIG120) Ex1	*NEUROD4*	13_Heterochrom/lo	Repressive	6_Weak_Enhancer	Other	714,361	16,238	94.23111519
HF (TIG120) Ex1	*HNF4A*	13_Heterochrom/lo	Repressive	13_Heterochrom/lo	Repressive	146,816	7,243	78.46132181
HF (TIG120) Ex1	*NKX2-5*	14_Repetitive/CNV	Other	3_Poised_Promoter	Bivalent	928,351	17,519	90.58334454
HF (TIG120) Ex2	*ERCC3*	1_Active_Promoter	Active	1_Active_Promoter	Active	64,406	5,514	57.90861068
HF (TIG120) Ex2	*GAPDH*	1_Active_Promoter	Active	1_Active_Promoter	Active	1,305,704	11,583	94.0751269
HF (TIG120) Ex2	*TBX6*	1_Active_Promoter	Active	2_Weak_Promoter	Active	360,681	6,413	86.83348228
HF (TIG120) Ex2	*FOXF1*	1_Active_Promoter	Active	3_Poised_Promoter	Bivalent	111,397	4,997	67.34682876
HF (TIG120) Ex2	*KLF4*	1_Active_Promoter	Active	3_Poised_Promoter	Bivalent	267,802	10,378	83.52716766
HF (TIG120) Ex2	*NR2F2*	1_Active_Promoter	Active	3_Poised_Promoter	Bivalent	389,266	10,073	87.68508927
HF (TIG120) Ex2	*TCF21*	1_Active_Promoter	Active	3_Poised_Promoter	Bivalent	1,502,189	21,495	96.24684326
HF (TIG120) Ex2	*TWIST1*	1_Active_Promoter	Active	3_Poised_Promoter	Bivalent	747,264	14,526	91.28328233
HF (TIG120) Ex2	*SNAI1*	2_Weak_Promoter	Active	2_Weak_Promoter	Active	1,744,747	8,004	96.64682372
HF (TIG120) Ex2	*GATA6*	2_Weak_Promoter	Active	3_Poised_Promoter	Bivalent	347,045	7,347	87.05388881
HF (TIG120) Ex2	*MESP1*	2_Weak_Promoter	Active	3_Poised_Promoter	Bivalent	1,462,650	11,856	95.37983798

HF (TIG120) Ex2	*SOX2*	3_Poised_Promoter	Bivalent	1_Active_Promoter	Active	3,165,556	24,478	98.73545368
HF (TIG120) Ex2	*MIXL1*	3_Poised_Promoter	Bivalent	2_Weak_Promoter	Active	1,521,050	25,001	96.54647755
HF (TIG120) Ex2	*COL2A1*	3_Poised_Promoter	Bivalent	3_Poised_Promoter	Bivalent	1,110,217	18,727	94.91396569
HF (TIG120) Ex2	*EOMES*	3_Poised_Promoter	Bivalent	3_Poised_Promoter	Bivalent	649,137	19,666	89.09083496
HF (TIG120) Ex2	*HOXA13*	3_Poised_Promoter	Bivalent	3_Poised_Promoter	Bivalent	2,719,822	16,390	98.03393805
HF (TIG120) Ex2	*NEUROD1*	3_Poised_Promoter	Bivalent	3_Poised_Promoter	Bivalent	1,936,121	26,532	95.44193877
HF (TIG120) Ex2	*NEUROG2*	3_Poised_Promoter	Bivalent	3_Poised_Promoter	Bivalent	1,522,296	26,580	95.57231926
HF (TIG120) Ex2	*SOX7*	3_Poised_Promoter	Bivalent	3_Poised_Promoter	Bivalent	337,013	11,012	89.02398603
HF (TIG120) Ex2	*ETS2*	4_Strong_Enhancer	Other	2_Weak_Promoter	Active	20,772	1,876	70.54753309
HF (TIG120) Ex2	*Intergenic 3*	7_Weak_Enhancer	Other	5_Strong_Enhancer	Other	462,219	21,786	85.98594134
HF (TIG120) Ex2	*POU5F1*	11_Weak_Txn	Other	2_Weak_Promoter	Active	1,674,996	15,716	93.24070174
HF (TIG120) Ex2	*NANOG_P*	11_Weak_Txn	Other	11_Weak_Txn	Other	371,934	11,465	81.49180106
HF (TIG120) Ex2	*NANOG*	12_Repressed	Repressive	2_Weak_Promoter	Active	83,769	7,352	76.05805737
HF (TIG120) Ex2	*ASCL1*	12_Repressed	Repressive	3_Poised_Promoter	Bivalent	180,072	8,544	82.70094471
HF (TIG120) Ex2	*ASCL2*	12_Repressed	Repressive	3_Poised_Promoter	Bivalent	581,831	14,415	87.34193322
HF (TIG120) Ex2	*EVX1*	12_Repressed	Repressive	3_Poised_Promoter	Bivalent	53,455	6,309	60.71425134
HF (TIG120) Ex2	*FOXA2*	12_Repressed	Repressive	3_Poised_Promoter	Bivalent	56,121	5,317	77.96644965
HF (TIG120) Ex2	*GATA4*	12_Repressed	Repressive	3_Poised_Promoter	Bivalent	559,262	14,728	92.92817122
HF (TIG120) Ex2	*GATA5*	12_Repressed	Repressive	3_Poised_Promoter	Bivalent	1,005,673	8,792	94.25284892
HF (TIG120) Ex2	*GSC*	12_Repressed	Repressive	3_Poised_Promoter	Bivalent	724,680	12,042	93.79819601
HF (TIG120) Ex2	*LHX1*	12_Repressed	Repressive	3_Poised_Promoter	Bivalent	164,191	7,764	82.09184051
HF (TIG120) Ex2	*PAX3*	12_Repressed	Repressive	3_Poised_Promoter	Bivalent	30,202	5,266	53.95657653

HF (TIG120) Ex2	*PAX6*	12_Repressed	Repressive	3_Poised_Promoter	Bivalent	1,388,857	15,915	92.94027646
HF (TIG120) Ex2	*SOX1*	12_Repressed	Repressive	3_Poised_Promoter	Bivalent	1,112,744	13,361	94.70915973
HF (TIG120) Ex2	*SOX17*	12_Repressed	Repressive	3_Poised_Promoter	Bivalent	691,844	13,248	93.82170477
HF (TIG120) Ex2	*SST*	12_Repressed	Repressive	3_Poised_Promoter	Bivalent	1,234,637	18,414	93.93603942
HF (TIG120) Ex2	*T*	12_Repressed	Repressive	3_Poised_Promoter	Bivalent	198,844	15,793	76.30205817
HF (TIG120) Ex2	*ZIC1*	12_Repressed	Repressive	3_Poised_Promoter	Bivalent	67,138	7,316	59.08958744
HF (TIG120) Ex2	*DPPA4*	13_Heterochrom/lo	Repressive	1_Active_Promoter	Active	846,550	20,694	92.30892757
HF (TIG120) Ex2	*Intergenic 1*	13_Heterochrom/lo	Repressive	4_Strong_Enhancer	Other	1,762,150	23,918	93.49944685
HF (TIG120) Ex2	*Intergenic 2*	13_Heterochrom/lo	Repressive	5_Strong_Enhancer	Other	1,972,301	17,117	95.75247674
HF (TIG120) Ex2	*Intergenic 4*	13_Heterochrom/lo	Repressive	5_Strong_Enhancer	Other	1,691,528	13,232	95.94663186
HF (TIG120) Ex2	*Intergenic 5*	13_Heterochrom/lo	Repressive	5_Strong_Enhancer	Other	1,436,111	25,520	92.67631924
HF (TIG120) Ex2	*NEUROD4*	13_Heterochrom/lo	Repressive	6_Weak_Enhancer	Other	824,843	16,230	93.01339324
HF (TIG120) Ex2	*HNF4A*	13_Heterochrom/lo	Repressive	13_Heterochrom/lo	Repressive	259,364	8,085	87.69836535
HF (TIG120) Ex2	*NKX2-5*	14_Repetitive/CNV	Other	3_Poised_Promoter	Bivalent	1,469,831	19,231	94.60582954
iPS (428C2) Ex1	*ACO2*	1_Active_Promoter	Active	1_Active_Promoter	Active	407,060	2,668	93.68306471
iPS (428C2) Ex1	*DPH3*	1_Active_Promoter	Active	1_Active_Promoter	Active	988,540	11,697	91.9235817
iPS (428C2) Ex1	*ERCC3*	1_Active_Promoter	Active	1_Active_Promoter	Active	52,978	4,415	50.22924559
iPS (428C2) Ex1	*GAPDH*	1_Active_Promoter	Active	1_Active_Promoter	Active	1,443,698	7,936	91.15343089
iPS (428C2) Ex1	*KDM2B*	1_Active_Promoter	Active	1_Active_Promoter	Active	981,800	8,548	93.91033036
iPS (428C2) Ex1	*KIF2A*	1_Active_Promoter	Active	1_Active_Promoter	Active	408,700	9,235	88.27352846
iPS (428C2) Ex1	*LIG1*	1_Active_Promoter	Active	1_Active_Promoter	Active	371,520	4,442	91.87366854
iPS (428C2) Ex1	*METAP2*	1_Active_Promoter	Active	1_Active_Promoter	Active	283,780	5,325	89.41164618

iPS (428C2) Ex1	*MITD1*	1_Active_Promoter	Active	1_Active_Promoter	Active	687,020	14,514	87.37478328
iPS (428C2) Ex1	*NCL*	1_Active_Promoter	Active	1_Active_Promoter	Active	703,380	10,592	88.46128149
iPS (428C2) Ex1	*STX5*	1_Active_Promoter	Active	1_Active_Promoter	Active	519,340	5,867	89.38964561
iPS (428C2) Ex1	*TAF2*	1_Active_Promoter	Active	1_Active_Promoter	Active	208,140	4,091	84.25149824
iPS (428C2) Ex1	*YBX1*	1_Active_Promoter	Active	1_Active_Promoter	Active	866,460	9,556	92.52965384
iPS (428C2) Ex1	*YTHDF2*	1_Active_Promoter	Active	1_Active_Promoter	Active	597,560	8,882	90.18723854
iPS (428C2) Ex1	*ZZZ3*	1_Active_Promoter	Active	1_Active_Promoter	Active	660,780	13,377	87.46052606
iPS (428C2) Ex1	*TBX6*	1_Active_Promoter	Active	2_Weak_Promoter	Active	234,553	7,161	84.56342552
iPS (428C2) Ex1	*FOXF1*	1_Active_Promoter	Active	3_Poised_Promoter	Bivalent	93,857	3,727	61.73493896
iPS (428C2) Ex1	*KLF4*	1_Active_Promoter	Active	3_Poised_Promoter	Bivalent	129,848	8,464	76.33821871
iPS (428C2) Ex1	*NR2F2*	1_Active_Promoter	Active	3_Poised_Promoter	Bivalent	286,098	7,019	84.97990217
iPS (428C2) Ex1	*TCF21*	1_Active_Promoter	Active	3_Poised_Promoter	Bivalent	917,824	15,154	92.46332951
iPS (428C2) Ex1	*TWIST1*	1_Active_Promoter	Active	3_Poised_Promoter	Bivalent	414,711	11,180	78.94532436
iPS (428C2) Ex1	*BCAT1*	1_Active_Promoter	Active	4_Strong_Enhancer	Other	602,460	7,454	90.73203983
iPS (428C2) Ex1	*DNMT3B*	2_Weak_Promoter	Active	1_Active_Promoter	Active	393,640	2,508	93.49487418
iPS (428C2) Ex1	*SNAI1*	2_Weak_Promoter	Active	2_Weak_Promoter	Active	1,403,105	8,735	96.17290203
iPS (428C2) Ex1	*GATA6*	2_Weak_Promoter	Active	3_Poised_Promoter	Bivalent	201,593	5,940	81.73037233
iPS (428C2) Ex1	*MESP1*	2_Weak_Promoter	Active	3_Poised_Promoter	Bivalent	1,341,623	9,570	94.14380294
iPS (428C2) Ex1	*SOX2*	3_Poised_Promoter	Bivalent	1_Active_Promoter	Active	2,473,905	18,118	96.42876151
iPS (428C2) Ex1	*MIXL1*	3_Poised_Promoter	Bivalent	2_Weak_Promoter	Active	1,035,958	18,825	94.10900147
iPS (428C2) Ex1	*PODXL*	3_Poised_Promoter	Bivalent	2_Weak_Promoter	Active	880,860	9,519	92.65496029
iPS (428C2) Ex1	*PTPRZ1*	3_Poised_Promoter	Bivalent	2_Weak_Promoter	Active	343,160	7,722	83.68010403

iPS (428C2) Ex1	*COL2A1*	3_Poised_Promoter	Bivalent	3_Poised_Promoter	Bivalent	701,368	12,594	90.61996316
iPS (428C2) Ex1	*EOMES*	3_Poised_Promoter	Bivalent	3_Poised_Promoter	Bivalent	410,409	12,401	85.64463107
iPS (428C2) Ex1	*HOXA13*	3_Poised_Promoter	Bivalent	3_Poised_Promoter	Bivalent	2,435,683	11,567	96.11852984
iPS (428C2) Ex1	*NEUROD1*	3_Poised_Promoter	Bivalent	3_Poised_Promoter	Bivalent	966,017	20,327	87.96749093
iPS (428C2) Ex1	*NEUROG2*	3_Poised_Promoter	Bivalent	3_Poised_Promoter	Bivalent	1,127,126	19,151	92.77686271
iPS (428C2) Ex1	*SOX7*	3_Poised_Promoter	Bivalent	3_Poised_Promoter	Bivalent	285,626	8,168	84.97622788
iPS (428C2) Ex1	*PRSS3*	3_Poised_Promoter	Bivalent	12_Repressed	Repressive	214,920	4,451	85.84543578
iPS (428C2) Ex1	*ETS2*	4_Strong_Enhancer	Other	2_Weak_Promoter	Active	8,026	1,322	62.48818302
iPS (428C2) Ex1	*Intergenic 3*	7_Weak_Enhancer	Other	5_Strong_Enhancer	Other	256,224	13,808	72.9674743
iPS (428C2) Ex1	*ANKRD1*	7_Weak_Enhancer	Other	12_Repressed	Repressive	375,600	6,948	86.20454348
iPS (428C2) Ex1	*TREM1*	7_Weak_Enhancer	Other	12_Repressed	Repressive	618,660	8,217	89.20564958
iPS (428C2) Ex1	*PRAME*	8_Insulator	Other	8_Insulator	Other	460,080	3,449	86.47361163
iPS (428C2) Ex1	*CBLN2*	8_Insulator	Other	12_Repressed	Repressive	475,720	4,743	89.71909581
iPS (428C2) Ex1	*POU5F1*	11_Weak_Txn	Other	2_Weak_Promoter	Active	1,444,853	8,207	86.24603291
iPS (428C2) Ex1	*NANOG_P*	11_Weak_Txn	Other	11_Weak_Txn	Other	234,335	8,322	80.58409428
iPS (428C2) Ex1	*FGFR2*	12_Repressed	Repressive	1_Active_Promoter	Active	1,011,120	8,043	93.48738275
iPS (428C2) Ex1	*LIN28B*	12_Repressed	Repressive	1_Active_Promoter	Active	692,140	11,281	91.32120682
iPS (428C2) Ex1	*MYCN*	12_Repressed	Repressive	1_Active_Promoter	Active	405,080	12,519	83.7475332
iPS (428C2) Ex1	*SALL4*	12_Repressed	Repressive	1_Active_Promoter	Active	913,620	6,737	95.29130539
iPS (428C2) Ex1	*NANOG*	12_Repressed	Repressive	2_Weak_Promoter	Active	101,789	5,741	70.14564293
iPS (428C2) Ex1	*ASCL1*	12_Repressed	Repressive	3_Poised_Promoter	Bivalent	157,442	5,278	80.51500313
iPS (428C2) Ex1	*ASCL2*	12_Repressed	Repressive	3_Poised_Promoter	Bivalent	401,762	9,010	84.62795634

iPS (428C2) Ex1	*EVX1*	12_Repressed	Repressive	3_Poised_Promoter	Bivalent	36,211	3,803	52.03174169
iPS (428C2) Ex1	*FOXA2*	12_Repressed	Repressive	3_Poised_Promoter	Bivalent	63,113	4,621	69.37292639
iPS (428C2) Ex1	*GATA4*	12_Repressed	Repressive	3_Poised_Promoter	Bivalent	553,413	10,623	90.23418587
iPS (428C2) Ex1	*GATA5*	12_Repressed	Repressive	3_Poised_Promoter	Bivalent	700,007	7,017	89.42840757
iPS (428C2) Ex1	*GSC*	12_Repressed	Repressive	3_Poised_Promoter	Bivalent	431,808	8,288	90.85926023
iPS (428C2) Ex1	*LHX1*	12_Repressed	Repressive	3_Poised_Promoter	Bivalent	157,561	5,764	74.42893483
iPS (428C2) Ex1	*PAX3*	12_Repressed	Repressive	3_Poised_Promoter	Bivalent	21,899	3,013	60.92037689
iPS (428C2) Ex1	*PAX6*	12_Repressed	Repressive	3_Poised_Promoter	Bivalent	1,885,087	14,241	92.85751545
iPS (428C2) Ex1	*SOX1*	12_Repressed	Repressive	3_Poised_Promoter	Bivalent	1,335,342	7,627	91.04691417
iPS (428C2) Ex1	*SOX17*	12_Repressed	Repressive	3_Poised_Promoter	Bivalent	781,046	11,683	92.57381024
iPS (428C2) Ex1	*SST*	12_Repressed	Repressive	3_Poised_Promoter	Bivalent	619,379	12,666	88.52452142
iPS (428C2) Ex1	*T*	12_Repressed	Repressive	3_Poised_Promoter	Bivalent	149,138	10,811	70.31041682
iPS (428C2) Ex1	*ZIC1*	12_Repressed	Repressive	3_Poised_Promoter	Bivalent	50,003	5,037	60.00285898
iPS (428C2) Ex1	*COLQ*	12_Repressed	Repressive	7_Weak_Enhancer	Other	518,140	8,451	87.62779702
iPS (428C2) Ex1	*DEFB132*	12_Repressed	Repressive	12_Repressed	Repressive	48,820	2,252	73.03625868
iPS (428C2) Ex1	*GAST*	12_Repressed	Repressive	12_Repressed	Repressive	611,160	5,374	62.78917605
iPS (428C2) Ex1	*HEMGN*	12_Repressed	Repressive	12_Repressed	Repressive	476,400	6,325	91.22903175
iPS (428C2) Ex1	*SLC34A1*	12_Repressed	Repressive	12_Repressed	Repressive	904,600	7,153	67.41625983
iPS (428C2) Ex1	*TNFSF8*	12_Repressed	Repressive	12_Repressed	Repressive	350,560	4,199	86.55947227
iPS (428C2) Ex1	*VPREB1*	12_Repressed	Repressive	12_Repressed	Repressive	280,000	2,635	91.4771741
iPS (428C2) Ex1	*DPPA4*	13_Heterochrom/lo	Repressive	1_Active_Promoter	Active	1,029,523	19,517	89.9139881
iPS (428C2) Ex1	*ZFP42*	13_Heterochrom/lo	Repressive	1_Active_Promoter	Active	646,220	7,799	89.93519223

iPS (428C2) Ex1	*Intergenic 1*	13_Heterochrom/lo	Repressive	4_Strong_Enhancer	Other	1,199,038	16,568	89.72553777
iPS (428C2) Ex1	*Intergenic 2*	13_Heterochrom/lo	Repressive	5_Strong_Enhancer	Other	1,603,505	13,197	94.11169033
iPS (428C2) Ex1	*Intergenic 4*	13_Heterochrom/lo	Repressive	5_Strong_Enhancer	Other	1,727,815	10,231	94.93365501
iPS (428C2) Ex1	*Intergenic 5*	13_Heterochrom/lo	Repressive	5_Strong_Enhancer	Other	1,002,928	19,607	87.62121822
iPS (428C2) Ex1	*NEUROD4*	13_Heterochrom/lo	Repressive	6_Weak_Enhancer	Other	619,821	11,012	90.29930299
iPS (428C2) Ex1	*ARHGEF38*	13_Heterochrom/lo	Repressive	12_Repressed	Repressive	358,020	7,241	81.69551188
iPS (428C2) Ex1	*FLRT1*	13_Heterochrom/lo	Repressive	12_Repressed	Repressive	709,680	6,646	89.40847268
iPS (428C2) Ex1	*GIMAP1*	13_Heterochrom/lo	Repressive	12_Repressed	Repressive	460,440	6,274	88.28413025
iPS (428C2) Ex1	*IGLL5*	13_Heterochrom/lo	Repressive	12_Repressed	Repressive	457,580	2,985	90.40797165
iPS (428C2) Ex1	*KRTAP5-11*	13_Heterochrom/lo	Repressive	12_Repressed	Repressive	167,840	4,653	82.38897939
iPS (428C2) Ex1	*MC3R*	13_Heterochrom/lo	Repressive	12_Repressed	Repressive	214,160	3,995	89.39560071
iPS (428C2) Ex1	*OBP2B*	13_Heterochrom/lo	Repressive	12_Repressed	Repressive	321,060	3,775	60.68448888
iPS (428C2) Ex1	*HNF4A*	13_Heterochrom/lo	Repressive	13_Heterochrom/lo	Repressive	179,649	6,894	81.07631334
iPS (428C2) Ex1	*SOX6*	13_Heterochrom/lo	Repressive	13_Heterochrom/lo	Repressive	540,320	7,667	90.08977251
iPS (428C2) Ex1	*NKX2-5*	14_Repetitive/CNV	Other	3_Poised_Promoter	Bivalent	1,085,549	12,418	90.22152152
iPS (428C2) Ex2	*ACO2*	1_Active_Promoter	Active	1_Active_Promoter	Active	492,080	3,106	92.68045236
iPS (428C2) Ex2	*DPH3*	1_Active_Promoter	Active	1_Active_Promoter	Active	909,520	13,251	90.1747557
iPS (428C2) Ex2	*ERCC3*	1_Active_Promoter	Active	1_Active_Promoter	Active	67,937	4,074	64.70364018
iPS (428C2) Ex2	*GAPDH*	1_Active_Promoter	Active	1_Active_Promoter	Active	1,517,433	8,089	91.14879712
iPS (428C2) Ex2	*KDM2B*	1_Active_Promoter	Active	1_Active_Promoter	Active	1,236,400	8,634	94.57564737
iPS (428C2) Ex2	*KIF2A*	1_Active_Promoter	Active	1_Active_Promoter	Active	405,960	10,324	86.30230886
iPS (428C2) Ex2	*LIG1*	1_Active_Promoter	Active	1_Active_Promoter	Active	383,000	4,615	93.15892529

iPS (428C2) Ex2	*METAP2*	1_Active_Promoter	Active	1_Active_Promoter	Active	430,460	6,572	88.35233384
iPS (428C2) Ex2	*MITD1*	1_Active_Promoter	Active	1_Active_Promoter	Active	859,880	15,692	87.86311004
iPS (428C2) Ex2	*NCL*	1_Active_Promoter	Active	1_Active_Promoter	Active	960,560	11,191	91.07629705
iPS (428C2) Ex2	*STX5*	1_Active_Promoter	Active	1_Active_Promoter	Active	522,620	5,693	87.71551392
iPS (428C2) Ex2	*TAF2*	1_Active_Promoter	Active	1_Active_Promoter	Active	252,040	4,865	82.75779948
iPS (428C2) Ex2	*YBX1*	1_Active_Promoter	Active	1_Active_Promoter	Active	1,074,520	10,206	92.43225619
iPS (428C2) Ex2	*YTHDF2*	1_Active_Promoter	Active	1_Active_Promoter	Active	698,220	9,072	87.21567421
iPS (428C2) Ex2	*ZZZ3*	1_Active_Promoter	Active	1_Active_Promoter	Active	890,000	14,597	89.89130769
iPS (428C2) Ex2	*TBX6*	1_Active_Promoter	Active	2_Weak_Promoter	Active	387,580	7,620	89.88711028
iPS (428C2) Ex2	*FOXF1*	1_Active_Promoter	Active	3_Poised_Promoter	Bivalent	199,418	4,412	71.18540151
iPS (428C2) Ex2	*KLF4*	1_Active_Promoter	Active	3_Poised_Promoter	Bivalent	224,016	8,726	78.95689312
iPS (428C2) Ex2	*NR2F2*	1_Active_Promoter	Active	3_Poised_Promoter	Bivalent	367,133	7,042	84.4172191
iPS (428C2) Ex2	*TCF21*	1_Active_Promoter	Active	3_Poised_Promoter	Bivalent	1,326,190	15,685	92.29347973
iPS (428C2) Ex2	*TWIST1*	1_Active_Promoter	Active	3_Poised_Promoter	Bivalent	697,331	11,726	87.02363724
iPS (428C2) Ex2	*BCAT1*	1_Active_Promoter	Active	4_Strong_Enhancer	Other	776,720	8,611	91.90066063
iPS (428C2) Ex2	*DNMT3B*	2_Weak_Promoter	Active	1_Active_Promoter	Active	511,980	2,969	93.08377226
iPS (428C2) Ex2	*SNAI1*	2_Weak_Promoter	Active	2_Weak_Promoter	Active	1,503,192	8,919	95.90425704
iPS (428C2) Ex2	*GATA6*	2_Weak_Promoter	Active	3_Poised_Promoter	Bivalent	415,434	6,434	83.78586032
iPS (428C2) Ex2	*MESP1*	2_Weak_Promoter	Active	3_Poised_Promoter	Bivalent	1,409,329	9,464	93.24781019
iPS (428C2) Ex2	*SOX2*	3_Poised_Promoter	Bivalent	1_Active_Promoter	Active	3,384,289	18,210	97.7786369
iPS (428C2) Ex2	*MIXL1*	3_Poised_Promoter	Bivalent	2_Weak_Promoter	Active	1,441,188	19,017	93.00448957
iPS (428C2) Ex2	*PODXL*	3_Poised_Promoter	Bivalent	2_Weak_Promoter	Active	982,540	10,538	93.20220048

iPS (428C2) Ex2	*PTPRZ1*	3_Poised_Promoter	Bivalent	2_Weak_Promoter	Active	393,120	9,015	85.27967761
iPS (428C2) Ex2	*COL2A1*	3_Poised_Promoter	Bivalent	3_Poised_Promoter	Bivalent	1,152,938	13,225	92.17633734
iPS (428C2) Ex2	*EOMES*	3_Poised_Promoter	Bivalent	3_Poised_Promoter	Bivalent	708,450	12,938	88.79768294
iPS (428C2) Ex2	*HOXA13*	3_Poised_Promoter	Bivalent	3_Poised_Promoter	Bivalent	2,518,859	12,096	95.27260125
iPS (428C2) Ex2	*NEUROD1*	3_Poised_Promoter	Bivalent	3_Poised_Promoter	Bivalent	1,617,898	20,637	92.47666462
iPS (428C2) Ex2	*NEUROG2*	3_Poised_Promoter	Bivalent	3_Poised_Promoter	Bivalent	1,566,173	19,374	94.21181368
iPS (428C2) Ex2	*SOX7*	3_Poised_Promoter	Bivalent	3_Poised_Promoter	Bivalent	404,842	8,133	88.99320487
iPS (428C2) Ex2	*PRSS3*	3_Poised_Promoter	Bivalent	12_Repressed	Repressive	204,220	5,610	85.26742967
iPS (428C2) Ex2	*ETS2*	4_Strong_Enhancer	Other	2_Weak_Promoter	Active	19,103	1,673	69.38741653
iPS (428C2) Ex2	*Intergenic 3*	7_Weak_Enhancer	Other	5_Strong_Enhancer	Other	523,746	15,420	81.60154948
iPS (428C2) Ex2	*ANKRD1*	7_Weak_Enhancer	Other	12_Repressed	Repressive	307,540	7,903	83.28942099
iPS (428C2) Ex2	*TREM1*	7_Weak_Enhancer	Other	12_Repressed	Repressive	517,820	9,094	88.40799502
iPS (428C2) Ex2	*PRAME*	8_Insulator	Other	8_Insulator	Other	429,160	3,754	92.26080759
iPS (428C2) Ex2	*CBLN2*	8_Insulator	Other	12_Repressed	Repressive	422,780	5,541	90.8390439
iPS (428C2) Ex2	*POU5F1*	11_Weak_Txn	Other	2_Weak_Promoter	Active	2,366,942	8,543	91.90962883
iPS (428C2) Ex2	*NANOG_P*	11_Weak_Txn	Other	11_Weak_Txn	Other	383,284	8,782	77.65015871
iPS (428C2) Ex2	*FGFR2*	12_Repressed	Repressive	1_Active_Promoter	Active	1,044,160	8,635	92.16663642
iPS (428C2) Ex2	*LIN28B*	12_Repressed	Repressive	1_Active_Promoter	Active	983,820	12,913	92.75647564
iPS (428C2) Ex2	*MYCN*	12_Repressed	Repressive	1_Active_Promoter	Active	516,320	13,498	84.81999786
iPS (428C2) Ex2	*SALL4*	12_Repressed	Repressive	1_Active_Promoter	Active	1,040,160	7,195	94.84701881
iPS (428C2) Ex2	*NANOG*	12_Repressed	Repressive	2_Weak_Promoter	Active	168,906	5,962	78.47937975
iPS (428C2) Ex2	*ASCL1*	12_Repressed	Repressive	3_Poised_Promoter	Bivalent	265,447	5,525	82.15246903

iPS (428C2) Ex2	*ASCL2*	12_Repressed	Repressive	3_Poised_Promoter	Bivalent	800,009	9,825	88.23608654
iPS (428C2) Ex2	*EVX1*	12_Repressed	Repressive	3_Poised_Promoter	Bivalent	75,273	4,877	64.4194064
iPS (428C2) Ex2	*FOXA2*	12_Repressed	Repressive	3_Poised_Promoter	Bivalent	151,812	5,503	73.48895596
iPS (428C2) Ex2	*GATA4*	12_Repressed	Repressive	3_Poised_Promoter	Bivalent	825,219	11,188	90.27895455
iPS (428C2) Ex2	*GATA5*	12_Repressed	Repressive	3_Poised_Promoter	Bivalent	1,344,116	7,239	93.37873921
iPS (428C2) Ex2	*GSC*	12_Repressed	Repressive	3_Poised_Promoter	Bivalent	982,463	9,042	91.17584778
iPS (428C2) Ex2	*LHX1*	12_Repressed	Repressive	3_Poised_Promoter	Bivalent	383,709	6,704	80.36153099
iPS (428C2) Ex2	*PAX3*	12_Repressed	Repressive	3_Poised_Promoter	Bivalent	39,749	3,732	60.76889258
iPS (428C2) Ex2	*PAX6*	12_Repressed	Repressive	3_Poised_Promoter	Bivalent	2,334,526	13,903	94.46192104
iPS (428C2) Ex2	*SOX1*	12_Repressed	Repressive	3_Poised_Promoter	Bivalent	2,227,927	8,065	93.99547796
iPS (428C2) Ex2	*SOX17*	12_Repressed	Repressive	3_Poised_Promoter	Bivalent	953,165	11,730	91.29257207
iPS (428C2) Ex2	*SST*	12_Repressed	Repressive	3_Poised_Promoter	Bivalent	1,130,128	13,361	91.3810084
iPS (428C2) Ex2	*T*	12_Repressed	Repressive	3_Poised_Promoter	Bivalent	285,738	11,840	79.05296682
iPS (428C2) Ex2	*ZIC1*	12_Repressed	Repressive	3_Poised_Promoter	Bivalent	65,987	5,197	58.149553
iPS (428C2) Ex2	*COLQ*	12_Repressed	Repressive	7_Weak_Enhancer	Other	455,040	9,978	87.71097046
iPS (428C2) Ex2	*DEFB132*	12_Repressed	Repressive	12_Repressed	Repressive	47,760	2,646	74.985122
iPS (428C2) Ex2	*GAST*	12_Repressed	Repressive	12_Repressed	Repressive	566,580	5,768	90.6476403
iPS (428C2) Ex2	*HEMGN*	12_Repressed	Repressive	12_Repressed	Repressive	364,500	7,519	88.02903471
iPS (428C2) Ex2	*SLC34A1*	12_Repressed	Repressive	12_Repressed	Repressive	798,180	6,931	90.18750569
iPS (428C2) Ex2	*TNFSF8*	12_Repressed	Repressive	12_Repressed	Repressive	285,540	4,936	83.95414498
iPS (428C2) Ex2	*VPREB1*	12_Repressed	Repressive	12_Repressed	Repressive	300,200	3,069	4.456583881
iPS (428C2) Ex2	*DPPA4*	13_Heterochrom/lo	Repressive	1_Active_Promoter	Active	1,477,838	19,689	92.34340923

iPS (428C2) Ex2	*ZFP42*	13_Heterochrom/lo	Repressive	1_Active_Promoter	Active	665,900	8,303	86.90327915
iPS (428C2) Ex2	*Intergenic 1*	13_Heterochrom/lo	Repressive	4_Strong_Enhancer	Other	1,841,248	17,539	91.94468326
iPS (428C2) Ex2	*Intergenic 2*	13_Heterochrom/lo	Repressive	5_Strong_Enhancer	Other	2,603,335	13,383	95.30693633
iPS (428C2) Ex2	*Intergenic 4*	13_Heterochrom/lo	Repressive	5_Strong_Enhancer	Other	1,909,362	10,536	94.07562838
iPS (428C2) Ex2	*Intergenic 5*	13_Heterochrom/lo	Repressive	5_Strong_Enhancer	Other	1,606,334	19,651	91.04429207
iPS (428C2) Ex2	*NEUROD4*	13_Heterochrom/lo	Repressive	6_Weak_Enhancer	Other	1,214,590	12,182	91.37413741
iPS (428C2) Ex2	*ARHGEF38*	13_Heterochrom/lo	Repressive	12_Repressed	Repressive	348,680	8,523	89.85198617
iPS (428C2) Ex2	*FLRT1*	13_Heterochrom/lo	Repressive	12_Repressed	Repressive	640,540	7,305	90.3703887
iPS (428C2) Ex2	*GIMAP1*	13_Heterochrom/lo	Repressive	12_Repressed	Repressive	376,160	7,052	88.88552774
iPS (428C2) Ex2	*IGLL5*	13_Heterochrom/lo	Repressive	12_Repressed	Repressive	472,440	3,263	94.17842815
iPS (428C2) Ex2	*KRTAP5-11*	13_Heterochrom/lo	Repressive	12_Repressed	Repressive	154,160	5,178	79.36726905
iPS (428C2) Ex2	*MC3R*	13_Heterochrom/lo	Repressive	12_Repressed	Repressive	190,100	4,213	84.56273459
iPS (428C2) Ex2	*OBP2B*	13_Heterochrom/lo	Repressive	12_Repressed	Repressive	248,620	3,989	88.35581545
iPS (428C2) Ex2	*HNF4A*	13_Heterochrom/lo	Repressive	13_Heterochrom/lo	Repressive	242,056	6,923	80.94709149
iPS (428C2) Ex2	*SOX6*	13_Heterochrom/lo	Repressive	13_Heterochrom/lo	Repressive	477,040	8,759	89.43504498
iPS (428C2) Ex2	*NKX2-5*	14_Repetitive/CNV	Other	3_Poised_Promoter	Bivalent	1,709,065	12,395	91.44024846
EED-sh1	*TWIST1*	1_Active_Promoter	Active	3_Poised_Promoter	Bivalent	*370,004*	5,250	88.07690372
EED-sh1	*HOXA13*	3_Poised_Promoter	Bivalent	3_Poised_Promoter	Bivalent	*482,561*	5,499	85.2835807
EED-sh1	*NEUROD1*	3_Poised_Promoter	Bivalent	3_Poised_Promoter	Bivalent	*616,371*	10,549	86.19168233
EED-sh1	*NEUROG2*	3_Poised_Promoter	Bivalent	3_Poised_Promoter	Bivalent	*595,542*	9,862	88.75569235
EED-sh1	*GSC*	12_Repressed	Repressive	3_Poised_Promoter	Bivalent	*342,858*	5,185	85.72373316
EED-sh1	*PAX6*	12_Repressed	Repressive	3_Poised_Promoter	Bivalent	*670,882*	7,671	86.77307331

EED-sh1	*SOX17*	12_Repressed	Repressive	3_Poised_Promoter	Bivalent	*807,112*	5,465	92.19738507
EED-sh1	*NKX2-5*	14_Repetitive/CNV	Other	3_Poised_Promoter	Bivalent	*663,297*	7,512	88.07494343
EED-sh2	*TWIST1*	1_Active_Promoter	Active	3_Poised_Promoter	Bivalent	*217,670*	6,680	85.45040551
EED-sh2	*HOXA13*	3_Poised_Promoter	Bivalent	3_Poised_Promoter	Bivalent	*289,058*	6,951	81.07729812
EED-sh2	*NEUROD1*	3_Poised_Promoter	Bivalent	3_Poised_Promoter	Bivalent	*569,455*	14,162	88.89976962
EED-sh2	*NEUROG2*	3_Poised_Promoter	Bivalent	3_Poised_Promoter	Bivalent	*651,423*	14,499	90.36403637
EED-sh2	*GSC*	12_Repressed	Repressive	3_Poised_Promoter	Bivalent	*500,495*	7,190	88.21220003
EED-sh2	*PAX6*	12_Repressed	Repressive	3_Poised_Promoter	Bivalent	*494,706*	9,701	84.6709085
EED-sh2	*SOX17*	12_Repressed	Repressive	3_Poised_Promoter	Bivalent	*825,543*	8,293	94.90404312
EED-sh2	*NKX2-5*	14_Repetitive/CNV	Other	3_Poised_Promoter	Bivalent	*461,339*	9,717	88.90373108
RING1B-sh1	*TWIST1*	1_Active_Promoter	Active	3_Poised_Promoter	Bivalent	*422,626*	7,461	90.1304663
RING1B-sh1	*HOXA13*	3_Poised_Promoter	Bivalent	3_Poised_Promoter	Bivalent	*384,041*	7,983	87.80536093
RING1B-sh1	*NEUROD1*	3_Poised_Promoter	Bivalent	3_Poised_Promoter	Bivalent	*632,045*	13,467	90.43231846
RING1B-sh1	*NEUROG2*	3_Poised_Promoter	Bivalent	3_Poised_Promoter	Bivalent	*716,996*	14,421	93.31987343
RING1B-sh1	*GSC*	12_Repressed	Repressive	3_Poised_Promoter	Bivalent	*468,919*	6,930	92.66688062
RING1B-sh1	*PAX6*	12_Repressed	Repressive	3_Poised_Promoter	Bivalent	*729,006*	10,054	91.5348058
RING1B-sh1	*SOX17*	12_Repressed	Repressive	3_Poised_Promoter	Bivalent	*906,769*	8,110	95.49602591
RING1B-sh1	*NKX2-5*	14_Repetitive/CNV	Other	3_Poised_Promoter	Bivalent	*600,484*	10,074	91.09899352
RING1B-sh2	*TWIST1*	1_Active_Promoter	Active	3_Poised_Promoter	Bivalent	*563,682*	6,660	89.76830074
RING1B-sh2	*HOXA13*	3_Poised_Promoter	Bivalent	3_Poised_Promoter	Bivalent	*734,762*	7,010	93.23048278
RING1B-sh2	*NEUROD1*	3_Poised_Promoter	Bivalent	3_Poised_Promoter	Bivalent	*901,090*	12,289	93.56916611
RING1B-sh2	*NEUROG2*	3_Poised_Promoter	Bivalent	3_Poised_Promoter	Bivalent	*967,034*	12,613	93.6064418

RING1B-sh2	*GSC*	12_Repressed	Repressive	3_Poised_Promoter	Bivalent	*625,269*	6,294	93.61752763
RING1B-sh2	*PAX6*	12_Repressed	Repressive	3_Poised_Promoter	Bivalent	*1,001,566*	9,316	91.37723024
RING1B-sh2	*SOX17*	12_Repressed	Repressive	3_Poised_Promoter	Bivalent	*1,074,198*	7,597	94.23570711
RING1B-sh2	*NKX2-5*	14_Repetitive/CNV	Other	3_Poised_Promoter	Bivalent	*951,039*	8,849	94.0952543
shNega	*TWIST1*	1_Active_Promoter	Active	3_Poised_Promoter	Bivalent	*589,571*	7,465	90.71135198
shNega	*HOXA13*	3_Poised_Promoter	Bivalent	3_Poised_Promoter	Bivalent	*503,685*	7,668	91.56367544
shNega	*NEUROD1*	3_Poised_Promoter	Bivalent	3_Poised_Promoter	Bivalent	*726,236*	13,549	91.2716323
shNega	*NEUROG2*	3_Poised_Promoter	Bivalent	3_Poised_Promoter	Bivalent	*723,357*	13,004	91.86989572
shNega	*GSC*	12_Repressed	Repressive	3_Poised_Promoter	Bivalent	316,160	6,272	85.20002717
shNega	*PAX6*	12_Repressed	Repressive	3_Poised_Promoter	Bivalent	*658,380*	9,198	87.70942795
shNega	*SOX17*	12_Repressed	Repressive	3_Poised_Promoter	Bivalent	*875,539*	7,702	92.33904808
shNega	*NKX2-5*	14_Repetitive/CNV	Other	3_Poised_Promoter	Bivalent	*363,205*	8,398	83.50651804
WDR5-sh1	*TWIST1*	1_Active_Promoter	Active	3_Poised_Promoter	Bivalent	*260,357*	6,099	83.87194367
WDR5-sh1	*HOXA13*	3_Poised_Promoter	Bivalent	3_Poised_Promoter	Bivalent	*298,018*	6,852	87.10021864
WDR5-sh1	*NEUROD1*	3_Poised_Promoter	Bivalent	3_Poised_Promoter	Bivalent	*438,847*	11,259	87.61322252
WDR5-sh1	*NEUROG2*	3_Poised_Promoter	Bivalent	3_Poised_Promoter	Bivalent	*405,701*	11,857	90.62421181
WDR5-sh1	*GSC*	12_Repressed	Repressive	3_Poised_Promoter	Bivalent	*257,122*	5,407	89.18027379
WDR5-sh1	*PAX6*	12_Repressed	Repressive	3_Poised_Promoter	Bivalent	*482,738*	8,499	87.89947254
WDR5-sh1	*SOX17*	12_Repressed	Repressive	3_Poised_Promoter	Bivalent	*636,918*	6,578	94.13279908
WDR5-sh1	*NKX2-5*	14_Repetitive/CNV	Other	3_Poised_Promoter	Bivalent	*445,222*	8,690	89.49720901
WDR5-sh2	*TWIST1*	1_Active_Promoter	Active	3_Poised_Promoter	Bivalent	*548,015*	6,072	92.12422813
WDR5-sh2	*HOXA13*	3_Poised_Promoter	Bivalent	3_Poised_Promoter	Bivalent	*490,114*	6,606	89.50613102

WDR5-sh2	*NEUROD1*	3_Poised_Promoter	Bivalent	3_Poised_Promoter	Bivalent	*603,793*	10,878	90.94101124
WDR5-sh2	*NEUROG2*	3_Poised_Promoter	Bivalent	3_Poised_Promoter	Bivalent	*709,042*	11,615	94.49004661
WDR5-sh2	*GSC*	12_Repressed	Repressive	3_Poised_Promoter	Bivalent	*370,659*	5,530	91.20746813
WDR5-sh2	*PAX6*	12_Repressed	Repressive	3_Poised_Promoter	Bivalent	*774,845*	8,339	90.88035921
WDR5-sh2	*SOX17*	12_Repressed	Repressive	3_Poised_Promoter	Bivalent	*853,763*	6,267	91.86051625
WDR5-sh2	*NKX2-5*	14_Repetitive/CNV	Other	3_Poised_Promoter	Bivalent	*904,421*	8,730	94.56034315

Supplementary Table 3. Sequenced read number and cis read ratio in each bait after removing PCR duplicates

Samples (cell type)	Bait	Total read #	Cis-chromosomal interaction #	Trans-chromosomal interaction#	Cis-chromosomal read % among total read
HF (TIG120) Ex1	*ASCL1*	61,965	17,423	44,542	28.11
HF (TIG120) Ex1	*ASCL2*	76,646	32,382	44,264	42.24
HF (TIG120) Ex1	*COL2A1*	108,383	52,818	55,565	48.73
HF (TIG120) Ex1	*DPPA4*	88,020	45,751	42,269	51.97
HF (TIG120) Ex1	*EOMES*	109,804	60,007	49,797	54.64
HF (TIG120) Ex1	*ERCC3*	22,107	10,730	11,377	48.53
HF (TIG120) Ex1	*ETS2*	10,239	4,311	5,928	42.1
HF (TIG120) Ex1	*EVX1*	23,605	12,524	11,081	53.05
HF (TIG120) Ex1	*FOXA2*	30,789	9,905	20,884	32.17
HF (TIG120) Ex1	*FOXF1*	47,405	19,193	28,212	40.48
HF (TIG120) Ex1	*GAPDH*	94,681	42,361	52,320	44.74
HF (TIG120) Ex1	*GATA4*	78,789	35,277	43,512	44.77
HF (TIG120) Ex1	*GATA5*	102,410	37,961	64,449	37.06
HF (TIG120) Ex1	*GATA6*	52,742	24,273	28,469	46.02
HF (TIG120) Ex1	*GSC*	81,673	34,727	46,946	42.51
HF (TIG120) Ex1	*HNF4A*	50,136	19,730	30,406	39.35
HF (TIG120) Ex1	*HOXA13*	119,539	74,858	44,681	62.62
HF (TIG120) Ex1	*KLF4*	60,760	36,530	24,230	60.12
HF (TIG120) Ex1	*LHX1*	52,588	17,831	34,757	33.9

HF (TIG120) Ex1	*MESP1*	102,836	47,150	55,686	45.84
HF (TIG120) Ex1	*MIXL1*	101,297	60,310	40,987	59.53
HF (TIG120) Ex1	*NANOG*	54,268	13,266	41,002	24.44
HF (TIG120) Ex1	*NANOG_P*	62,101	25,034	37,067	40.31
HF (TIG120) Ex1	*NEUROD1*	95,002	61,605	33,397	64.84
HF (TIG120) Ex1	*NEUROD4*	95,678	36,426	59,252	38.07
HF (TIG120) Ex1	*NEUROG2*	108,223	66,922	41,301	61.83
HF (TIG120) Ex1	*NKX2-5*	111,321	60,778	50,543	54.59
HF (TIG120) Ex1	*NR2F2*	78,617	39,609	39,008	50.38
HF (TIG120) Ex1	*PAX3*	11,540	6,793	4,747	58.86
HF (TIG120) Ex1	*PAX6*	98,726	41,747	56,979	42.28
HF (TIG120) Ex1	*POU5F1*	77,322	35,467	41,855	45.86
HF (TIG120) Ex1	*Intergenic 1*	116,693	57,241	59,452	49.05
HF (TIG120) Ex1	*Intergenic 2*	133,599	58,572	75,027	43.84
HF (TIG120) Ex1	*Intergenic 3*	68,691	39,033	29,658	56.82
HF (TIG120) Ex1	*Intergenic 4*	118,176	47,711	70,465	40.37
HF (TIG120) Ex1	*Intergenic 5*	103,027	62,314	40,713	60.48
HF (TIG120) Ex1	*SNAI1*	138,122	57,287	80,835	41.47
HF (TIG120) Ex1	*SOX1*	88,539	35,452	53,087	40.04
HF (TIG120) Ex1	*SOX17*	70,184	30,473	39,711	43.41
HF (TIG120) Ex1	*SOX2*	119,294	75,014	44,280	62.88
HF (TIG120) Ex1	*SOX7*	52,695	22,037	30,658	41.81

HF (TIG120) Ex1	*SST*	100,199	51,482	48,717	51.37
HF (TIG120) Ex1	*T*	57,231	33,005	24,226	57.66
HF (TIG120) Ex1	*TBX6*	62,192	26,472	35,720	42.56
HF (TIG120) Ex1	*TCF21*	103,835	60,775	43,060	58.53
HF (TIG120) Ex1	*TWIST1*	83,126	49,586	33,540	59.65
HF (TIG120) Ex1	*ZIC1*	19,021	12,216	6,805	64.22
HF (TIG120) Ex2	*ASCL1*	82,224	15,822	66,402	19.24
HF (TIG120) Ex2	*ASCL2*	107,126	41,415	65,711	38.66
HF (TIG120) Ex2	*COL2A1*	133,985	60,131	73,854	44.87
HF (TIG120) Ex2	*DPPA4*	109,917	46,250	63,667	42.07
HF (TIG120) Ex2	*EOMES*	125,220	60,571	64,649	48.37
HF (TIG120) Ex2	*ERCC3*	24,118	10,195	13,923	42.27
HF (TIG120) Ex2	*ETS2*	15,038	5,817	9,221	38.68
HF (TIG120) Ex2	*EVX1*	31,067	15,647	15,420	50.36
HF (TIG120) Ex2	*FOXA2*	48,671	12,218	36,453	25.1
HF (TIG120) Ex2	*FOXF1*	58,202	21,629	36,573	37.16
HF (TIG120) Ex2	*GAPDH*	117,042	46,499	70,543	39.72
HF (TIG120) Ex2	*GATA4*	97,462	39,476	57,986	40.5
HF (TIG120) Ex2	*GATA5*	139,980	50,612	89,368	36.15
HF (TIG120) Ex2	*GATA6*	67,749	28,891	38,858	42.64
HF (TIG120) Ex2	*GSC*	105,208	39,165	66,043	37.22
HF (TIG120) Ex2	*HNF4A*	70,324	25,088	45,236	35.67

HF (TIG120) Ex2	*HOXA13*	138,380	80,838	57,542	58.41
HF (TIG120) Ex2	*KLF4*	73,813	43,609	30,204	59.08
HF (TIG120) Ex2	*LHX1*	69,153	21,601	47,552	31.23
HF (TIG120) Ex2	*MESP1*	126,342	54,577	71,765	43.19
HF (TIG120) Ex2	*MIXL1*	124,107	70,357	53,750	56.69
HF (TIG120) Ex2	*NANOG*	74,062	13,463	60,599	18.17
HF (TIG120) Ex2	*NANOG_P*	79,948	29,640	50,308	37.07
HF (TIG120) Ex2	*NEUROD1*	125,779	77,253	48,526	61.41
HF (TIG120) Ex2	*NEUROD4*	118,535	33,725	84,810	28.45
HF (TIG120) Ex2	*NEUROG2*	130,948	76,209	54,739	58.19
HF (TIG120) Ex2	*NKX2-5*	139,924	75,401	64,523	53.88
HF (TIG120) Ex2	*NR2F2*	95,135	43,522	51,613	45.74
HF (TIG120) Ex2	*PAX3*	15,056	7,939	7,117	52.72
HF (TIG120) Ex2	*PAX6*	118,493	45,019	73,474	37.99
HF (TIG120) Ex2	*POU5F1*	100,909	43,696	57,213	43.3
HF (TIG120) Ex2	*Intergenic 1*	138,159	60,915	77,244	44.09
HF (TIG120) Ex2	*Intergenic 2*	155,346	64,347	90,999	41.42
HF (TIG120) Ex2	*Intergenic 3*	94,557	51,036	43,521	53.97
HF (TIG120) Ex2	*Intergenic 4*	140,116	53,643	86,473	38.28
HF (TIG120) Ex2	*Intergenic 5*	142,623	74,712	67,911	52.38
HF (TIG120) Ex2	*SNAI1*	165,002	65,686	99,316	39.8
HF (TIG120) Ex2	*SOX1*	114,099	39,653	74,446	34.75

HF (TIG120) Ex2	*SOX17*	82,173	30,552	51,621	37.18
HF (TIG120) Ex2	*SOX2*	140,691	81,691	59,000	58.06
HF (TIG120) Ex2	*SOX7*	68,733	25,481	43,252	37.07
HF (TIG120) Ex2	*SST*	123,654	60,295	63,359	48.76
HF (TIG120) Ex2	*T*	84,409	42,883	41,526	50.8
HF (TIG120) Ex2	*TBX6*	84,201	34,331	49,870	40.77
HF (TIG120) Ex2	*TCF21*	117,519	64,599	52,920	54.96
HF (TIG120) Ex2	*TWIST1*	98,707	52,871	45,836	53.56
HF (TIG120) Ex2	*ZIC1*	23,504	14,102	9,402	59.99
iPS (428C2) Ex1	*ASCL1*	45,094	19,145	25,949	42.45
iPS (428C2) Ex1	*ASCL2*	82,295	42,289	40,006	51.38
iPS (428C2) Ex1	*COL2A1*	132,281	56,345	75,936	42.59
iPS (428C2) Ex1	*DPPA4*	121,818	67,399	54,419	55.32
iPS (428C2) Ex1	*EOMES*	87,522	55,898	31,624	63.86
iPS (428C2) Ex1	*ERCC3*	23,610	11,677	11,933	49.45
iPS (428C2) Ex1	*ETS2*	10,593	4,416	6,177	41.68
iPS (428C2) Ex1	*EVX1*	24,397	16,582	7,815	67.96
iPS (428C2) Ex1	*FOXA2*	34,950	22,563	12,387	64.55
iPS (428C2) Ex1	*FOXF1*	43,000	25,370	17,630	59
iPS (428C2) Ex1	*GAPDH*	95,259	49,175	46,084	51.62
iPS (428C2) Ex1	*GATA4*	90,624	48,150	42,474	53.13
iPS (428C2) Ex1	*GATA5*	128,025	57,110	70,915	44.6

iPS (428C2) Ex1	*GATA6*	56,870	29,053	27,817	51.08
iPS (428C2) Ex1	*GSC*	87,829	44,763	43,066	50.96
iPS (428C2) Ex1	*HNF4A*	70,006	27,918	42,088	39.87
iPS (428C2) Ex1	*HOXA13*	118,612	69,823	48,789	58.86
iPS (428C2) Ex1	*KLF4*	61,545	33,040	28,505	53.68
iPS (428C2) Ex1	*LHX1*	56,708	32,001	24,707	56.43
iPS (428C2) Ex1	*MESP1*	118,534	56,557	61,977	65.9
iPS (428C2) Ex1	*MIXL1*	130,956	63,743	67,213	48.67
iPS (428C2) Ex1	*NANOG*	66,375	21,624	44,751	32.57
iPS (428C2) Ex1	*NANOG_P*	80,563	33,193	47,370	41.2
iPS (428C2) Ex1	*NEUROD1*	116,423	76,291	40,132	65.52
iPS (428C2) Ex1	*NEUROD4*	108,876	40,615	68,261	37.3
iPS (428C2) Ex1	*NEUROG2*	135,078	74,376	60,702	55.06
iPS (428C2) Ex1	*NKX2-5*	127,837	70,136	57,701	54.86
iPS (428C2) Ex1	*NR2F2*	76,871	50,125	26,746	65.2
iPS (428C2) Ex1	*PAX3*	11,943	8,717	3,226	72.98
iPS (428C2) Ex1	*PAX6*	120,853	73,113	47,740	60.49
iPS (428C2) Ex1	*POU5F1*	95,638	49,204	46,434	51.44
iPS (428C2) Ex1	*Intergenic 1*	112,557	55,552	57,005	49.35
iPS (428C2) Ex1	*Intergenic 2*	152,879	72,387	80,492	47.34
iPS (428C2) Ex1	*Intergenic 3*	84,578	43,773	40,805	51.75
iPS (428C2) Ex1	*Intergenic 4*	141,812	56,282	85,530	39.68

iPS (428C2) Ex1	*Intergenic 5*	138,607	78,244	60,363	56.45
iPS (428C2) Ex1	*SNAI1*	152,650	64,250	88,400	42.08
iPS (428C2) Ex1	*SOX1*	101,881	57,825	44,056	56.75
iPS (428C2) Ex1	*SOX17*	79,908	45,537	34,371	56.98
iPS (428C2) Ex1	*SOX2*	150,493	83,193	67,300	55.28
iPS (428C2) Ex1	*SOX7*	64,348	36,661	27,687	56.97
iPS (428C2) Ex1	*SST*	113,824	49,666	64,158	43.63
iPS (428C2) Ex1	*T*	75,427	46,195	29,232	61.24
iPS (428C2) Ex1	*TBX6*	82,975	33,808	49,167	40.74
iPS (428C2) Ex1	*TCF21*	111,689	53,136	58,553	47.57
iPS (428C2) Ex1	*TWIST1*	83,407	47,885	35,522	57.41
iPS (428C2) Ex1	*ZIC1*	20,026	14,765	5,261	73.72
iPS (428C2) Ex1	*LIN28B*	66,071	33,101	32,970	50.1
iPS (428C2) Ex1	*YTHDF2*	72,845	31,804	41,041	43.66
iPS (428C2) Ex1	*YBX1*	77,529	33,271	44,258	42.91
iPS (428C2) Ex1	*ZZZ3*	83,648	39,909	43,739	47.71
iPS (428C2) Ex1	*MYCN*	74,581	45,848	28,733	61.47
iPS (428C2) Ex1	*MITD1*	149,571	42,052	107,519	28.12
iPS (428C2) Ex1	*DPH3*	98,359	39,184	59,175	39.84
iPS (428C2) Ex1	*ZFP42*	77,834	38,925	38,909	50.01
iPS (428C2) Ex1	*KIF2A*	65,966	30,205	35,761	45.79
iPS (428C2) Ex1	*PTPRZ1*	58,344	25,360	32,984	43.47

iPS (428C2) Ex1	*PODXL*	76,763	34,575	42,188	45.04
iPS (428C2) Ex1	*TAF2*	36,778	11,997	24,781	32.62
iPS (428C2) Ex1	*FGFR2*	83,530	36,880	46,650	44.15
iPS (428C2) Ex1	*STX5*	68,165	31,055	37,110	45.56
iPS (428C2) Ex1	*BCAT1*	66,583	25,945	40,638	38.97
iPS (428C2) Ex1	*METAP2*	52,783	23,129	29,654	43.82
iPS (428C2) Ex1	*KDM2B*	100,621	39,815	60,806	39.57
iPS (428C2) Ex1	*NCL*	83,869	36,845	47,024	43.93
iPS (428C2) Ex1	*LIG1*	71,571	25,456	46,115	35.57
iPS (428C2) Ex1	*DNMT3B*	28,984	12,246	16,738	42.25
iPS (428C2) Ex1	*SALL4*	108,411	40,444	67,967	37.31
iPS (428C2) Ex1	*ACO2*	63,538	18,184	45,354	28.62
iPS (428C2) Ex1	*ARHGEF38*	58,397	19,816	38,581	33.93
iPS (428C2) Ex1	*SLC34A1*	91,916	37,517	54,399	40.82
iPS (428C2) Ex1	*GIMAP1*	61,182	21,548	39,634	35.22
iPS (428C2) Ex1	*PRSS3*	55,328	14,991	40,337	27.09
iPS (428C2) Ex1	*HEMGN*	76,067	23,365	52,702	30.72
iPS (428C2) Ex1	*TNFSF8*	57,870	17,256	40,614	29.82
iPS (428C2) Ex1	*OBP2B*	59,080	17,553	41,527	29.71
iPS (428C2) Ex1	*ANKRD1*	55,763	23,200	32,563	41.6
iPS (428C2) Ex1	*SOX6*	71,550	28,635	42,915	40.02
iPS (428C2) Ex1	*FLRT1*	78,681	27,604	51,077	35.08

iPS (428C2) Ex1	*KRTAP5-11*	41,672	16,562	25,110	39.74
iPS (428C2) Ex1	*GAST*	73,433	25,528	47,905	34.76
iPS (428C2) Ex1	*DEFB132*	23,044	6,746	16,298	29.27
iPS (428C2) Ex1	*MC3R*	40,778	18,915	21,863	46.39
iPS (428C2) Ex1	*VPREB1*	51,228	14,107	37,121	27.54
iPS (428C2) Ex1	*PRAME*	68,137	28,204	39,933	41.39
iPS (428C2) Ex1	*IGLL5*	60,384	17,559	42,825	29.08
iPS (428C2) Ex1	*COLQ*	82,191	32,388	49,803	39.41
iPS (428C2) Ex1	*CBLN2*	69,218	21,902	47,316	31.64
iPS (428C2) Ex1	*TREM1*	84,098	29,132	54,966	34.64
iPS (428C2) Ex2	*ASCL1*	47,912	19,652	28,260	41.01
iPS (428C2) Ex2	*ASCL2*	91,976	48,086	43,890	52.28
iPS (428C2) Ex2	*COL2A1*	137,159	60,532	76,627	44.13
iPS (428C2) Ex2	*DPPA4*	124,363	70,147	54,216	56.4
iPS (428C2) Ex2	*EOMES*	92,101	60,151	31,950	65.3
iPS (428C2) Ex2	*ERCC3*	21,381	10,570	10,811	49.43
iPS (428C2) Ex2	*ETS2*	14,890	6,192	8,698	41.58
iPS (428C2) Ex2	*EVX1*	32,730	22,195	10,535	67.81
iPS (428C2) Ex2	*FOXA2*	47,011	30,154	16,857	64.14
iPS (428C2) Ex2	*FOXF1*	51,545	30,920	20,625	59.98
iPS (428C2) Ex2	*GAPDH*	97,766	50,686	47,080	51.84
iPS (428C2) Ex2	*GATA4*	98,001	52,853	45,148	53.93

iPS (428C2) Ex2	*GATA5*	134,326	60,140	74,186	44.77
iPS (428C2) Ex2	*GATA6*	63,153	32,736	30,417	51.83
iPS (428C2) Ex2	*GSC*	98,677	51,560	47,117	52.25
iPS (428C2) Ex2	*HNF4A*	68,356	28,545	39,811	41.75
iPS (428C2) Ex2	*HOXA13*	121,511	74,091	47,420	60.97
iPS (428C2) Ex2	*KLF4*	65,041	37,268	27,773	57.29
iPS (428C2) Ex2	*LHX1*	71,414	40,893	30,521	57.26
iPS (428C2) Ex2	*MESP1*	116,665	57,072	59,593	48.91
iPS (428C2) Ex2	*MIXL1*	133,655	66,233	67,422	49.55
iPS (428C2) Ex2	*NANOG*	68,933	22,821	46,112	33.1
iPS (428C2) Ex2	*NANOG_P*	86,404	36,936	49,468	42.74
iPS (428C2) Ex2	*NEUROD1*	120,813	81,222	39,591	67.22
iPS (428C2) Ex2	*NEUROD4*	118,352	45,719	72,633	38.62
iPS (428C2) Ex2	*NEUROG2*	137,897	79,297	58,600	57.5
iPS (428C2) Ex2	*NKX2-5*	131,584	75,155	56,429	57.11
iPS (428C2) Ex2	*NR2F2*	76,561	50,775	25,786	66.31
iPS (428C2) Ex2	*PAX3*	15,562	11,474	4,088	73.73
iPS (428C2) Ex2	*PAX6*	116,248	72,319	43,929	62.21
iPS (428C2) Ex2	*POU5F1*	99,819	51,972	47,847	52.06
iPS (428C2) Ex2	*Intergenic 1*	123,363	60,707	62,656	49.21
iPS (428C2) Ex2	*Intergenic 2*	157,121	74,664	82,457	47.52
iPS (428C2) Ex2	*Intergenic 3*	99,219	52,999	46,220	53.41

iPS (428C2) Ex2	*Intergenic 4*	146,250	59,813	86,437	40.89
iPS (428C2) Ex2	*Intergenic 5*	138,746	83,063	55,683	59.86
iPS (428C2) Ex2	*SNAI1*	153,933	66,731	87,202	43.35
iPS (428C2) Ex2	*SOX1*	108,434	61,865	46,569	57.05
iPS (428C2) Ex2	*SOX17*	82,396	47,336	35,060	57.44
iPS (428C2) Ex2	*SOX2*	152,306	89,643	62,663	58.85
iPS (428C2) Ex2	*SOX7*	65,557	38,631	26,926	58.92
iPS (428C2) Ex2	*SST*	120,878	55,083	65,795	45.56
iPS (428C2) Ex2	*T*	89,024	54,986	34,038	61.76
iPS (428C2) Ex2	*TBX6*	88,009	36,691	51,318	41.69
iPS (428C2) Ex2	*TCF21*	115,275	58,448	56,827	50.7
iPS (428C2) Ex2	*TWIST1*	90,456	53,133	37,323	58.73
iPS (428C2) Ex2	*ZIC1*	20,933	15,517	5,416	74.12
iPS (428C2) Ex2	*LIN28B*	71,346	37,876	33,470	53.09
iPS (428C2) Ex2	*YTHDF2*	70,016	33,842	36,174	48.33
iPS (428C2) Ex2	*YBX1*	76,329	36,734	39,595	48.13
iPS (428C2) Ex2	*ZZZ3*	78,247	43,987	34,260	56.22
iPS (428C2) Ex2	*MYCN*	69,613	47,414	22,199	68.11
iPS (428C2) Ex2	*MITD1*	146,771	44,281	102,490	30.17
iPS (428C2) Ex2	*DPH3*	90,811	42,653	48,158	46.97
iPS (428C2) Ex2	*ZFP42*	74,635	40,607	34,028	54.41
iPS (428C2) Ex2	*KIF2A*	60,293	31,873	28,420	52.86

iPS (428C2) Ex2	*PTPRZ1*	57,857	28,638	29,219	49.5
iPS (428C2) Ex2	*PODXL*	75,730	38,192	37,538	50.43
iPS (428C2) Ex2	*TAF2*	38,405	13,677	24,728	35.61
iPS (428C2) Ex2	*FGFR2*	80,903	40,212	40,691	49.7
iPS (428C2) Ex2	*STX5*	61,883	30,772	31,111	49.73
iPS (428C2) Ex2	*BCAT1*	69,618	30,155	39,463	43.31
iPS (428C2) Ex2	*METAP2*	55,398	26,630	28,768	48.07
iPS (428C2) Ex2	*KDM2B*	92,430	41,694	50,736	45.11
iPS (428C2) Ex2	*NCL*	82,648	40,438	42,210	48.93
iPS (428C2) Ex2	*LIG1*	64,533	26,555	37,978	41.15
iPS (428C2) Ex2	*DNMT3B*	30,117	13,944	16,173	46.3
iPS (428C2) Ex2	*SALL4*	101,945	43,558	58,387	42.73
iPS (428C2) Ex2	*ACO2*	71,622	23,141	48,481	32.31
iPS (428C2) Ex2	*ARHGEF38*	60,052	23,183	36,869	38.6
iPS (428C2) Ex2	*SLC34A1*	84,084	38,849	45,235	46.2
iPS (428C2) Ex2	*GIMAP1*	58,248	23,395	34,853	40.16
iPS (428C2) Ex2	*PRSS3*	57,922	17,955	39,967	31
iPS (428C2) Ex2	*HEMGN*	73,427	25,779	47,648	35.11
iPS (428C2) Ex2	*TNFSF8*	58,816	19,456	39,360	33.08
iPS (428C2) Ex2	*OBP2B*	55,935	18,529	37,406	33.13
iPS (428C2) Ex2	*ANKRD1*	54,425	25,859	28,566	47.51
iPS (428C2) Ex2	*SOX6*	69,297	31,123	38,174	44.91

iPS (428C2) Ex2		*FLRT1*	76,582	30,642	45,940	40.01
iPS (428C2) Ex2		*KRTAP5-11*	39,188	18,219	20,969	46.49
iPS (428C2) Ex2		*GAST*	69,903	28,238	41,665	40.4
iPS (428C2) Ex2		*DEFB132*	22,265	7,231	15,034	32.48
iPS (428C2) Ex2		*MC3R*	37,751	18,779	18,972	49.74
iPS (428C2) Ex2		*VPREB1*	52,224	15,907	36,317	30.46
iPS (428C2) Ex2		*PRAME*	64,501	29,271	35,230	45.38
iPS (428C2) Ex2		*IGLL5*	59,417	19,462	39,955	32.75
iPS (428C2) Ex2		*COLQ*	83,935	37,087	46,848	44.19
iPS (428C2) Ex2		*CBLN2*	69,744	24,769	44,975	35.51
iPS (428C2) Ex2		*TREM1*	80,927	32,120	48,807	39.69
shNega		*TWIST1*	45746	35605	10141	77.83
		GSC	48901	33086	15815	67.65
		SOX17	43630	32580	11050	74.67
		NEUROD1	63714	53015	10699	83.2
		NKX2-5	68496	47009	21487	68.63
		NEUROG2	75258	57589	17669	76.52
		PAX6	64115	48031	16084	74.91
		HOXA13	62343	48299	14044	77.47
EED	sh1	*TWIST1*	29424	23117	6307	78.56
		GSC	37483	25618	11865	68.34
		SOX17	29109	21915	7194	75.28

EED	sh1	*NEUROD1*	45564	39037	6527	85.67
		NKX2-5	57883	40506	17377	69.97
		NEUROG2	51379	40595	10784	79.01
		PAX6	48627	37185	11442	76.46
		HOXA13	44361	35826	8535	80.76
	sh2	*TWIST1*	40194	30600	9594	76.13
		GSC	60946	40607	20339	66.62
		SOX17	47189	34869	12320	73.89
		NEUROD1	68122	56816	11306	83.4
		NKX2-5	81175	55190	25985	67.98
		NEUROG2	84055	63688	20367	75.76
		PAX6	66918	49681	17237	74.24
		HOXA13	60889	47829	13060	78.55
WDR5	sh1	*TWIST1*	39295	21313	17982	54.23
		GSC	48731	22962	25769	47.11
		SOX17	41628	22062	19566	52.99
		NEUROD1	56198	33838	22360	60.21
		NKX2-5	76918	36286	40632	47.17
		NEUROG2	73793	38646	35147	52.37
		PAX6	62908	33237	29671	52.83
		HOXA13	59245	31654	27591	53.42
	sh2	*TWIST1*	38274	23031	15243	60.17

WDR5	sh2	*GSC*	46447	24580	21867	52.92
		SOX17	38211	22074	16137	57.76
		NEUROD1	52315	34316	17999	65.59
		NKX2-5	74784	39682	35102	53.06
		NEUROG2	69504	39957	29547	57.48
		PAX6	59040	34317	24723	58.12
		HOXA13	56112	32918	23194	58.66
RING1B	sh1	*TWIST1*	48326	32393	15933	67.03
		GSC	61136	35946	25190	58.79
		SOX17	48742	31956	16786	65.56
		NEUROD1	67511	49186	18325	72.85
		NKX2-5	86717	50311	36406	58.01
		NEUROG2	85229	55178	30051	64.74
		PAX6	72365	47264	25101	65.31
		HOXA13	67717	45080	22637	66.57
	sh2	*TWIST1*	42211	31842	10369	75.43
		GSC	50579	33173	17406	65.58
		SOX17	42358	30985	11373	73.15
		NEUROD1	59719	48559	11160	81.31
		NKX2-5	74620	48949	25671	65.59
		NEUROG2	72101	53726	18375	74.51
		PAX6	65279	47528	17751	72.8

HOXA13	59442	45014	14428	75.72

Supplementary Table 4. Spearman's correlation coefficients of interaction frequencies (hit%) between biological duplicates or cell types (HDF and iPS)

Cis interaction

Bait	iPS (428C2) Ex.1 vs. Ex. 2	TIG120 Ex.1 vs. Ex. 2	Ex.1 iPS (428C2) vs. Ex.1 HDF (TIG120)	Ex.2 iPS (428C2) vs. Ex.2 HDF (TIG120)
ASCL1	0.772569	0.808567	0.687868	0.675739
ASCL2	0.908834	0.845173	0.681867	0.714829
DPPA4	0.898698	0.886055	0.522457	0.517504
ERCC3	0.553455	0.665026	0.454103	0.433568
ETS2	0.72659	0.876538	0.549591	0.608938
EVX1	0.71608	0.753794	0.582966	0.649208
FOXF1	0.796859	0.81846	0.660651	0.685862
GATA6	0.891303	0.909459	0.571612	0.559717
HNF4A	0.937826	0.913496	0.851767	0.880292
HOXA13	0.889001	0.919806	0.754725	0.763992
MIXL1	0.899496	0.926537	0.758836	0.772816
NANOG	0.720583	0.672871	0.567468	0.544335
NANOG_P	0.862057	0.865239	0.751869	0.742993
NEUROD1	0.860934	0.894531	0.821707	0.828041
NEUROD4	0.747489	0.890442	0.438555	0.478332
POU5F1	0.802559	0.879102	0.715549	0.723304
Intergenic 4	0.941639	0.923671	0.768317	0.773221
Intergenic 5	0.77461	0.827361	0.478508	0.492985
SOX1	0.822855	0.93335	0.472113	0.488847
SOX17	0.90174	0.794176	0.733489	0.752261
SOX2	0.877147	0.908627	0.645948	0.663819
SOX7	0.807695	0.746657	0.639901	0.655964
T	0.853468	0.876578	0.559127	0.555226
TCF21	0.864998	0.907361	0.833115	0.83703
COL2A1	0.866404	0.89035	0.594865	0.579211
EOMES	0.743827	0.82127	0.703268	0.713344
FOXA2	0.78941	0.85149	0.529421	0.544481
GAPDH	0.861951	0.914264	0.672864	0.670391
GATA4	0.881109	0.771912	0.692833	0.694226
GATA5	0.955289	0.954394	0.834064	0.840987

GSC	0.923541	0.903567	0.797043	0.806319
KLF4	0.885263	0.950321	0.70905	0.725528
LHX1	0.904248	0.811504	0.192042	0.203118
MESP1	0.945441	0.945875	0.625648	0.626271
NEUROG2	0.899891	0.936203	0.811235	0.801021
NKX2-5	0.842743	0.916825	0.749377	0.745742
NR2F2	0.889598	0.901352	0.821101	0.830427
PAX3	0.584575	0.646676	0.480375	0.534003
PAX6	0.902329	0.86084	0.8185	0.829571
Intergenic 1	0.822934	0.893322	0.708118	0.706065
Intergenic 2	0.863868	0.904491	0.61683	0.623375
Intergenic 3	0.824658	0.874356	0.677823	0.706988
SNAI1	0.957611	0.938518	0.778115	0.769223
SST	0.734616	0.824002	0.653117	0.682422
TBX6	0.942792	0.90883	0.689991	0.7108
TWIST1	0.795683	0.814517	0.724801	0.719319
ZIC1	0.694096	0.770364	0.691923	0.691856

Trans interaction

Bait	iPS (428C2) Ex.1 vs. Ex. 2	TIG120 Ex.1 vs. Ex. 2	Ex.1 iPS (428C2) vs. Ex.1 HDF (TIG120)	Ex.2 iPS (428C2) vs. Ex.2 HDF (TIG120)
ASCL1	0.007059	0.041234	0.011536	0.013784
ASCL2	0.390434	0.079882	0.121384	0.099337
DPPA4	0.202582	0.101223	-0.03951	-0.04287
ERCC3	0.020515	0.035124	0.026441	0.006837
ETS2	0.031435	0.069926	0.023408	0.039453
EVX1	0.073273	0.06591	0.034059	0.054355
FOXF1	0.258531	0.12493	0.125584	0.098653
GATA6	0.223103	0.237493	0.150946	0.129321
HNF4A	0.227909	0.093473	0.117345	0.114042
HOXA13	0.220735	0.215356	0.131671	0.121403
MIXL1	0.277145	0.152828	0.145235	0.146536
NANOG	0.13654	0.047784	0.015774	0.001821
NANOG_P	0.328964	0.157201	0.166044	0.160985
NEUROD1	0.081756	0.063047	0.043118	0.045717

NEUROD4	0.133474	0.137904	0.010459	-0.00229
POU5F1	0.445565	0.205351	0.228508	0.218927
Intergenic 4	0.276282	0.189079	0.169337	0.140599
Intergenic 5	0.108128	0.138783	0.026856	0.019182
SOX1	0.456733	0.15103	0.029726	0.022792
SOX17	0.159301	0.06513	0.055337	0.032729
SOX2	0.150953	0.150374	0.053317	0.059746
SOX7	0.257129	0.055782	0.027246	0.017499
T	0.21629	0.144051	0.065275	0.058196
TCF21	0.066578	0.055582	0.034211	0.053541
COL2A1	0.221902	0.154545	0.132439	0.114018
EOMES	0.039328	0.03132	0.010112	0.01293
FOXA2	0.161573	0.055772	0.040817	0.041478
GAPDH	0.392923	0.377449	0.286904	0.27553
GATA4	0.371711	0.058153	0.067052	0.043456
GATA5	0.486393	0.491211	0.327071	0.346671
GSC	0.307858	0.089159	0.05687	0.03378
KLF4	0.052661	0.247278	0.05892	0.039981
LHX1	0.333818	0.061474	0.057953	0.045542
MESP1	0.365542	0.372095	0.248492	0.241829
NEUROG2	0.148083	0.090609	0.071916	0.060861
NKX2-5	0.435849	0.238319	0.207023	0.218663
NR2F2	0.116074	0.085382	0.070125	0.054729
PAX3	0.022021	0.025368	0.01491	0.019375
PAX6	0.225059	0.076548	0.0564	0.041321
Intergenic 1	0.165129	0.072472	0.073151	0.07299
Intergenic 2	0.513089	0.344947	0.278557	0.271427
Intergenic 3	0.115721	0.087777	0.072557	0.056525
SNAI1	0.533467	0.594063	0.374487	0.354629
SST	0.083872	0.04465	0.049581	0.051783
TBX6	0.359307	0.422943	0.262079	0.263366
TWIST1	0.063345	0.051169	0.022156	0.027533
ZIC1	0.031034	0.01523	0.011822	0.019244
